# Promising anticancer agents based on 8-hydroxyquinoline hydrazone copper(II) complexes

**DOI:** 10.3389/fchem.2023.1106349

**Published:** 2023-03-21

**Authors:** Nádia Ribeiro, Ipek Bulut, Baris Sergi, Vivien Pósa, Gabriella Spengler, Giuseppe Sciortino, Vânia André, Liliana P. Ferreira, Tarita Biver, Valeria Ugone, Eugenio Garribba, João Costa-Pessoa, Éva A. Enyedy, Ceyda Acilan, Isabel Correia

**Affiliations:** ^1^ Centro de Química Estrutural, Institute of Molecular Sciences, and Departamento de Engenharia Química, Instituto Superior Técnico, Universidade de Lisboa, Lisbon, Portugal; ^2^ Graduate School of Health Sciences, Koc University, Istanbul, Türkiye; ^3^ MTA-SZTE Lendület Functional Metal Complexes Research Group, Department of Inorganic and Analytical Chemistry, University of Szeged, Szeged, Hungary; ^4^ Department of Medical Microbiology, Albert Szent-Györgyi Health Center and Faculty of Medicine, University of Szeged, Szeged, Hungary; ^5^ Institute of Chemical Research of Catalonia (ICIQ), The Barcelona Institute of Science and Technology, Tarragona, Spain; ^6^ Department of Physics, University of Coimbra, Coimbra, Portugal; ^7^ Biosystems and Integrative Sciences Institute (BioISI), Faculdade de Ciências, Universidade de Lisboa, Lisbon, Portugal; ^8^ Department of Chemistry and Industrial Chemistry, University of Pisa, Pisa, Italy; ^9^ Istituto di Chimica Biomolecolare, Consiglio Nazionale delle Ricerche, Sassari, Italy; ^10^ Dipartimento di Medicina, Chirurgia e Farmacia, Università di Sassari, Sassari, Italy; ^11^ School of Medicine, Koc University, Istanbul, Türkiye; ^12^ Research Center for Translational Medicine (KUTTAM), Koc University, Istanbul, Türkiye

**Keywords:** Schiff bases, anticancer, speciation, antibacterial, 8-hydoxyquinoline, copper complexes

## Abstract

We report the synthesis and characterization of a group of benzoylhydrazones (L^n^) derived from 2-carbaldehyde-8-hydroxyquinoline and benzylhydrazides containing distinct *para* substituents (R = H, Cl, F, CH_3_, OCH_3_, OH and NH_2_, for L^1-7^, respectively; in L^8^ isonicotinohydrazide was used instead of benzylhydrazide). Cu(II) complexes were prepared by reaction of each benzoylhydrazone with Cu(II) acetate. All compounds were characterized by elemental analysis and mass spectrometry as well as by FTIR, UV-visible absorption, NMR or electron paramagnetic resonance spectroscopies. Complexes isolated in the solid state (**1–8**) are either formulated as [Cu(HL)acetate] (with L^1^ and L^4^) or as [Cu(L^n^)]_3_ (*n* = 2, 3, 5, 6, 7 and 8). Single crystal X-ray diffraction studies were done for L^5^ and [Cu(L^5^)]_3_, confirming the trinuclear formulation of several complexes. Proton dissociation constants, lipophilicity and solubility were determined for all free ligands by UV-Vis spectrophotometry in 30% (v/v) DMSO/H_2_O. Formation constants were determined for [Cu(LH)], [Cu(L)] and [Cu(LH_−1_)] for L = L^1^, L^5^ and L^6^, and also [Cu(LH_−2_)] for L = L^6^, and binding modes are proposed, [Cu(L)] predominating at physiological pH. The redox properties of complexes formed with L^1^, L^5^ and L^6^ are investigated by cyclic voltammetry; the formal redox potentials fall in the range of +377 to +395 mV vs. NHE. The binding of the Cu(II)-complexes to bovine serum albumin was evaluated by fluorescence spectroscopy, showing moderate-to-strong interaction and suggesting formation of a ground state complex. The interaction of L^1^, L^3^, L^5^ and L^7^, and of the corresponding complexes with *calf thymus* DNA was evaluated by thermal denaturation. The antiproliferative activity of all compounds was evaluated in malignant melanoma (A-375) and lung (A-549) cancer cells. The complexes show higher activity than the corresponding free ligand, and most complexes are more active than cisplatin. Compounds **1, 3, 5**, and **8** were selected for additional studies: while these complexes induce reactive oxygen species and double-strand breaks in both cancer cells, their ability to induce cell-death by apoptosis varies. Within the set of compounds tested, **8** emerges as the most promising one, presenting low IC_50_ values, and high induction of oxidative stress and DNA damage, which eventually lead to high rates of apoptosis.

## 1 Introduction

Cancer is a leading cause of death in developed countries, with a significant expense to human lives. Despite the success of platinum metallodrugs, drawbacks such as intrinsic and acquired chemoresistance, as well as unspecific action leading to toxic side effects, drive researchers towards the search for alternative and safer chemotherapeutics, which may also take advantage of their different mechanisms of action. One alternative is the use of metallodrugs in which distinct metal ions may be explored to overcome these drawbacks and take advantage of different reactivity, electronic features and redox chemistry.

Copper is a signalling metal ion involved in cell growth and proliferation. The field of cuproplasia (regulated copper-dependent cell proliferation) has just begun to be explored, but has already given proof of its importance in fighting cancer (Ge et al., 2022). It is known that during tumour growth and metastasis cancer cells have an increased need for copper since this metal ion is involved in cellular processes such as signalling, oxidative phosphorylation, cell growth, proliferation, angiogenesis and autophagy (Lelièvre et al., 2020). This may be used as a strategy to target cancer cells, and develop anticancer treatments to interrupt, deplete, or increase copper concentration in tumours.

Despite being an essential nutrient to humans, copper homeostasis must be strictly maintained. Under biological conditions, copper is mostly bound to peptides and proteins, preventing uncontrolled redox activity. Its deficiency is detrimental, but its excess results in increased cellular oxidative stress. Organic molecules which act as chelating moieties can bind and sequester metal ions, or can act as ionophores that cross the cell membrane when still bound to the metal ion and release it inside the cell (Denoyer et al., 2015). In the intracellular environment, the Cu(II) complexes can be reduced by cellular reductants such as glutathione (GSH); the formed Cu(I) complex can be re-oxidized producing reactive oxygen species (ROS), or as Cu(I) has high affinity for GSH, the release of the original ligand is also possible.

8-Hydroxyquinoline (8HQ) and its derivatives are versatile compounds and have been explored in the design of various classes of anticancer compounds, acting through different mechanisms of action (Song et al., 2015). Numerous studies provided proof of the direct relationship between the ability of these molecules to act as metal chelators and their antiproliferative activity (Prachayasittikul et al., 2013). Binding of biologically active molecules to metal ions may alter their pharmacokinetic characteristics and improve their therapeutic potential, thus the prospective synergistic effect of having the 8HQ scaffold, with known therapeutic activity (Zhai et al., 2010; Jiang et al., 2011; Oliveri et al., 2012; Prachayasittikul et al., 2013; Song et al., 2015; Oliveri and Vecchio, 2016; Gupta et al., 2021), chelated to a metal ion, with its potential for involvement in redox processes and its diversity in structure and electronic features, has been considered an attractive approach to develop therapeutic drugs (Correia et al., 2014; Ndagi et al., 2017; Pape et al., 2018; Matos et al., 2019; Ribeiro et al., 2022). For example, zinc and copper complexes of 5,7-dihalo-substituted-8-hydroxyquinolines were reported as highly cytotoxic agents against hepatoma, ovarian and non-small-cell lung human tumour cells, with IC_50_ values from 1.4 nM to 32.13 μM (Liu et al., 2013).

8-Hydroxy-2-quinolinecarbaldehyde showed promising antiproliferative results both *in vitro* and *in vivo* (Chan et al., 2013; Lam et al., 2016). Moreover, it can be used as starting material for the synthesis of new 8HQ-based ligands, which allow expanding the coordination ability of the parent 8HQ (Albrecht et al., 2005). Also, its coordination to metal ions further fosters the design and development of new anticancer agents since metal ions chelation by 8HQs has been highlighted as a key factor in their ability to induce cell death (Zhang et al., 2008; Hickey et al., 2011; Xie and Peng, 2017; Zhu et al., 2019). Barilli *et al.* reported on a family of 2-substituted-8HQs, that showed promising antiproliferative results on HeLa cervical cancer cells in the presence of iron and copper (Barilli et al., 2014). Chen and co-workers showed that Cu(II) and Ni(II) complexes with 2-((2-(pyridin-2-yl)hydrazono)methyl)quinoline-8-ol present high cytotoxic activity against seven different cancer cells. The Cu(II) compound induced caspase-dependent apoptosis and caused cancer cell cycle arrest in the S phase (Chang et al., 2016). Rigolino and co-workers (Rogolino et al., 2017) reported on a series of 8-hydroxyquinoline thiosemicarbazones and their Cu(II) and Zn(II) complexes which displayed cytostatic activity in different cancer cells, the most active Cu(II) complex showing an IC_50_ value lower than 1 μM. Ali *et al.* studied mixed ligand Cu(II) complexes with 8HQ derivatives and their antiproliferative effect in breast cancer cells (Ali et al., 2021).

Recently, we reported on a series of six 8HQ-benzohydrazones and their coordination to V(IV)O ions (Ribeiro et al., 2022). Cytotoxicity on a melanoma cell line occurred in the low micromolar range and increased generation of ROS-induced DNA damage was observed. Annexin V and Caspase 3/7 flow cytometry assays indicated cell death by apoptosis. The fluoro-substituted metal complex was identified as the one showing the highest apoptosis and induction of DNA double-strand breaks. In this report, we synthesized two new ligands and Cu(II) complexes with the full series. The Cu(II) complexes show high potential as anticancer agents. The assays performed *in vitro* regarding their chemical and biological behaviour indicate that their redox properties are, at least partly, responsible for their cytotoxic action. They can interact with GSH and ascorbic acid (AA), increasing the ROS concentration in the cells, and triggering cell defences that lead to cell death.

## 2 Experimental

Details on the experimental part are included in Supplementary Material, section S1, and only the synthetic procedure/characterization aspects are included here.

### 2.1 Chemicals and apparatuses

Benzoic hydrazide (98%), 4-chlorobenzhydrazide (98%), 4-fluorobenzoic hydrazide (96%), 4-methoxybenzhydrazide (97%), *p*-toluic hydrazide (99%), and 4-aminobenzoic hydrazide (95%) were all from Aldrich, while 4-hydroxybenzhydrazide (puriss) was from Merck and isoniazid (≥99%) was from Fluka. [Cu(AcO)_2_]·H_2_O from Panreac was used as received. Dioxane (Fluka), dimethyl sulfoxide (DMSO, Riedel-de-Haën), methanol (MeOH, Riedel-de-Haën) and ethanol (EtOH, Fluka) were all analytical grade and used without further purification. 4-(2-Hydroxyethyl)-1-piperazineethanesulfonic acid (HEPES), tetrabutylammonium nitrate (TBAN), glutathione and ascorbic acid were purchased from Sigma-Aldrich in puriss quality. KCl, HCl, KOH, DMSO, EDTA and potassium hydrogenphtalate were obtained from Molar Chemicals (Hungary) and used without further purification. Milli-Q ultra-pure water was used for the preparation of all solutions. For the reactions with AA and GSH, the pH of the solutions was adjusted to 7.4 with HEPES (50 mM). CuCl_2_ stock solution was prepared by the dissolution of CuCl_2_ in water and the concentration was determined by complexometry. When necessary, the pH was set to the appropriate value by the addition of HCl or KOH solutions. Elemental analysis for C, H and N, were carried out on a FISONS EA 1108 CHNS-O apparatus at *Laboratório de Análises of Instituto Superior Técnico*, while ESI-MS spectra of methanolic solutions of the compounds in both positive and negative modes were measured in a 500-M Varian Ion Trap Mass Spectrometer. Proton, carbon and correlation NMR spectra were obtained on a Bruker Avance II + 300 (UltraShieldTM Magnet) spectrometer operating at 300 MHz for proton and at 75.4 MHz for carbon, at room temperature; the chemical shifts are reported in ppm using tetramethylsilane as the internal reference. A JASCO FT/IR 4100 spectrophotometer was used for recording the infra-red spectra and electronic absorption spectra (UV-Vis) were recorded ether with a Perkin Elmer Lambda 35 (in the characterization studies) or an Agilent Cary 8,454 diode array spectrophotometer (in the spectrophotometric speciation studies). UV-vis spectrophotometers were thermostated to within ±0.1°C by Haake water baths. EPR spectra were recorded at 120 K with an X-band (9.4 GHz) Bruker EMX spectrometer equipped with an HP 53150 A microwave frequency counter with the following instrumental settings: microwave frequency, 9.40–9.41 GHz; microwave power, 20 mW; time constant, 163.8 m; modulation frequency, 100 kHz; modulation amplitude, 4 G; sweep time, 335.5 s; resolution 4,096 points. Magnetization measurements as a function of temperature were performed using a SQUID magnetometer (Quantum Design MPMS). The molar magnetic susceptibility values were subtracted for diamagnetism of the constituent atoms, estimated from Pascal constants, to isolate the copper spin contribution; the corrected molar susceptibility is designated by *χ*_m_. Software PHI (Chilton et al., 2013) was used to fit an isotropic spin Hamiltonian (exchange coupling and Zeeman effect components) to the *χ*_m_, *χ*_m_^−1^, and χ_m_T vs. temperature curves.

### 2.2 Synthesis of the ligand precursors

**SCHEME 1:**
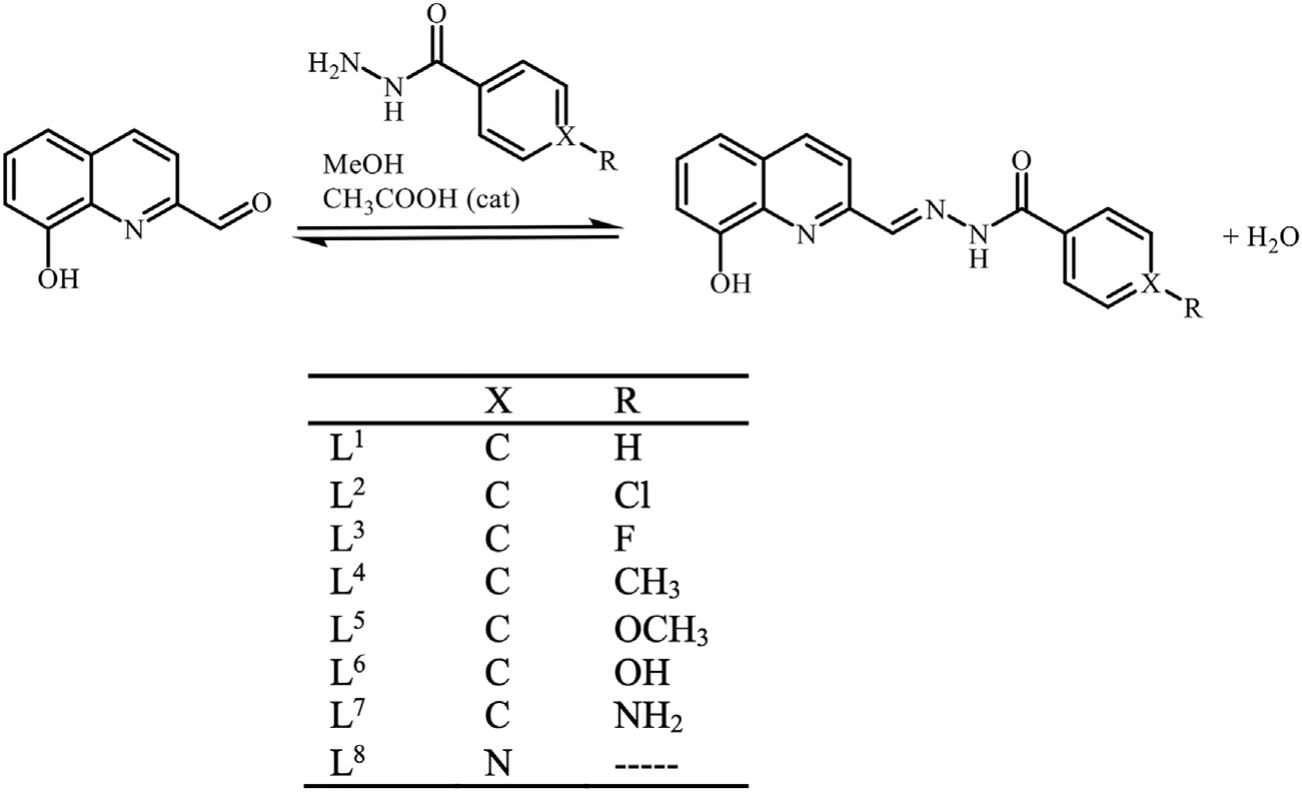
General scheme for the ligand precursors’ syntheses. Abbreviations L^1^ – L^8^ represent globally all possible protonation forms of the compounds. Only when specifying a particular protonation form, the protons will be included in the abbreviation (LH_n_ or H_n_L).

The synthesis of the ligand precursors was done by adapting a published procedure (Liu and Yang, 2009a) – Scheme 1. The synthesis and characterization of L^1^-L^6^ were described in a previous publication (Ribeiro et al., 2022). Briefly, the carbaldehyde was dissolved in *ca.* 10 mL of MeOH and stirred with a few drops of glacial acetic acid. The corresponding benzohydrazide was added as a solid to the previous solution and left to reflux for 6 h. When a solid started to separate from the reaction mixture, it was allowed to cool to room temperature and then in the freezer for further precipitation. The solid product was finally collected by filtration, washed with ice-cold MeOH and dried under vacuum in a desiccator over silica-gel.

Crystals of L^5^ suitable for single crystal X-ray diffraction (SCXRD) were obtained from an acetone solution of the compound kept at room temperature for a few weeks.

L^7^: Bright yellow solid. Yield: 62.3%. Elem. analysis for C_17_H_14_N_4_O_2_·0.25H_2_O [found (calcd.)]: C, 65.5% (65.69%); H, 4.5% (4.70%); N, 18.0% (18.03%). ESI-MS *m/z* (−) 305.40 (calcd. for [C_17_H_14_N_4_O_2_−H]^−^ 305.31) (+) 306.99 (calcd. for [C_17_H_14_N_4_O_2_+H]^+^ 307.31). FTIR (KBr pellet, cm^−1^) 3,441–3,318 (OH and primary amine), 3,212 (br), 3,109–3,046 (aromatic CH), 1,645 (carbonyl), 1,625 (CN_quinoline_), 1,548 (imine). UV-Vis [DMSO, λ, nm (ε, M^−1^cm^−1^)] 265 (2.2 × 10^4^), 284 (2.8 × 10^4^), 295 (2.9 × 10^4^), 350 (2.4 × 10^4^).

L^8^: Bright yellow solid. Yield: 71.1%. Elem. analysis for C_16_H_12_N_4_O_2_·0.25H_2_O [found (calcd.)]: C, 64.8% (64.75%); H, 4.2% (4.24%); N, 18.8% (18.88%). ESI-MS *m/z* (−) 291.21 (calcd. for [C_16_H_12_N_4_O_2_−H]^−^ 291.28) (+) 293.13 (calcd. for [C_16_H_12_N_4_O_2_+H]^+^ 293.30). FTIR (KBr pellet, cm^−1^) 3,445, 3,326, 3,046 (aromatic CH), 2,860 (azomethine CH), 1,679 (carbonyl), 1,549 (imine). UV-Vis [DMSO, λ, nm (ε, M^−1^cm^−1^)] 260 (2.0 × 10^4^), 295 (2.35 × 10^4^), 344 (1.3 × 10^4^), 356 (1.37 × 10^4^), 400 (1.5 × 10^3^).

### 2.3 Synthesis of the copper(II) complexes

In a round-bottom flask, 0.25 mmol of the corresponding ligand precursor were suspended in *ca.* 10 ml of MeOH. A few drops of 0.1 M KOH solution in MeOH were added till pH ≈ 9. In a vial, 0.25 mmol of [Cu(AcO)_2_]**·**H_2_O were dissolved in MeOH and added to the ligand precursors’ solution. The mixture was stirred for *ca.* 4 h at room temperature. In general, the ligand’s suspensions presented a yellow-milky aspect and, upon the addition of the blue copper solution, the reaction mixture gained a darker colour with the formation of a reddish-brown solid. The solids were collected by filtration, washed with ice-cold MeOH and dried under vacuum in a desiccator over silica-gel.

**[Cu**(**HL** ^**1**^ **)(AcO)]** (**1**): Red solid. Yield: 85.7%. Elem. analysis for C_19_H_15_N_3_O_4_Cu∙H_2_O [found (calcd.)]: C, 52.8% (52.96%); H, 3.5% (3.98%); N, 9.7% (9.75%). ESI-MS *m/z* (−) 410.96 (calcd. for [C_19_H_15_N_3_O_4_Cu−H]^−^ 411.03). FTIR (KBr pellet, cm^−1^) 3,415 (br), 3,054, 1,664 (br), 1,586, 1,512, 1,491, 1,454. UV-Vis [DMSO, λ, nm (ε, M^−1^cm^−1^)] 277 (2.18 × 10^4^), 331 (2.3 × 10^4^), 390 (1.2 × 10^4^), 478 (2.4 × 10^3^).
**[Cu**(**L** ^**2**^ **)]**_**3**_ (**2**): Red solid. Yield: 95.3%. Elem. analysis for 3×{C_17_H_10_N_3_O_2_ClCu∙0.5H_2_O∙0.25MeOH} [found (calcd.)]: C, 51.0% (51.25%); H, 2.7% (2.99%); N, 10.1% (10.39%). ESI-MS *m/z* (−) 385.23 (calcd. for [C_17_H_10_N_3_O_2_ClCu−H]^−^ 384.97). FTIR (KBr pellet, cm^−1^) 3,428 (br), 3,021, 1,662 (br), 1,593, 1,506, 1,487, 1,446. UV-Vis [DMSO, λ, nm (ε, M^−1^cm^−1^)] 282 (sh, 9.0 × 10^3^), 332 (8.0 × 10^3^), 405 (6.0 × 10^3^), 428 (5.5 × 10^3^), 556 (3.8 × 10^3^).
**[Cu**(**L** ^**3**^ **)]**_**3**_ (**3**): Dark red solid. Yield: 66.2%. Elem. analysis for 3×{C_17_H_10_N_3_O_2_FCu∙1.25H_2_O} [found (calcd.)]: C, 52.1% (51.91%); H, 2.8% (3.20%); N, 10.5% (10.68%). ESI-MS *m/z* (−) 369.39 (calcd. for [C_17_H_10_N_3_O_2_FCu−H]^−^ 369.00); *m/z* (+) 371.08 (calcd. for [C_17_H_10_N_3_O_2_FCu + H]^+^ 371.02). FTIR (KBr pellet, cm^−1^) 3,423 (br), 3,020, 1,662 (br), 1,601, 1,509, 1,493, 1,446. UV-Vis [DMSO, λ, nm (ε, M^−1^cm^−1^)] 280 (sh, 1.3 × 10^4^), 327 (1.25 × 10^4^), 394 (7.0 × 10^3^), 425 (4.5 × 10^3^), 535 (1.8 × 10^3^).
**[Cu**(**HL** ^**4**^ **)(AcO)]** (**4**): Reddish-brown solid. Yield: 74.9%. Elem. analysis for C_20_H_17_N_3_O_4_Cu∙0.5H_2_O [found (calcd.)]: C, 54.9% (55.11%); H, 4.0% (4.16%); N, 9.6% (9.64%). ESI-MS *m/z* (+) 367.10 (calcd. for [C_20_H_17_N_3_O_4_Cu−AcO]^+^ 367.04). FTIR (KBr pellet, cm^−1^) 3,420 (br), 3,052, 1,662 (br), 1,608, 1,518, 1,496, 1,452. UV-Vis [DMSO, λ, nm (ε, M^−1^cm^−1^)] 278 (2.35 × 10^4^), 327 (2.0 × 10^4^), 402 (1.25 × 10^4^), 426 (sh, 7.0 × 10^3^), 540 (br, 1.0 × 10^3^).
**[Cu**(**L** ^**5**^ **)]**_**3**_ (**5**): Reddish-brown solid. Yield: 39.9%. Elem. analysis for 3×{C_18_H_13_N_3_O_3_Cu∙1.75H_2_O} [found (calcd.)]: C, 52.1% (52.17%); H, 3.6% (4.01%); N, 9.7% (10.14%). ESI-MS *m/z* (+) 383.08 (calcd. for [C_18_H_15_N_3_O_4_Cu + H]^+^ 383.03). FTIR (KBr pellet, cm^−1^) 3,386 (br), 3,046, 1,657 (br), 1,603, 1,506, 1,485, 1,449. UV-Vis [DMSO, λ, nm (ε, M^−1^cm^−1^)] 281 (1.45 × 10^4^), 335 (1.5 × 10^4^), 399 (9.0 × 10^3^), 429 (sh, 6.0 × 10^3^), 560 (br, 9.0 × 10^2^). Crystals suitable for SCXRD were obtained after a few weeks from the mother liquor (methanol).
**[Cu**(**L** ^**6**^ **)]**_**3**_ (**6**)**:** Brown solid. Yield: 62.0%. Elem. analysis for 3×{C_17_H_11_N_3_O_3_Cu∙1.5H_2_O} [found (calcd.)]: C, 51.7% (51.58%); H, 3.2% (3.56%); N, 10.5% (10.61%). ESI-MS *m/z* (+) 367.23 (calcd. for [C_17_H_13_N_3_O_4_Cu−H]^−^ 367.00). FTIR (KBr pellet, cm^−1^) 3,414 (br), 3,060, 1,648 (br), 1,605, 1,524, 1,496, 1,454. UV-Vis [DMSO, λ, nm (ε, M^−1^cm^−1^)] 281 (2.25 × 10^4^), 332 (1.9 × 10^4^), 407 (1.2 × 10^4^), 478 (br, 3.0 × 10^3^), 549 (br, 1.1 × 10^3^).
**[Cu**(**L** ^**7**^ **)]**_**3**_ (**7**)**:** Brown solid. Yield: 59.0%. Elem. analysis for 3×{C_17_H_12_N_4_O_2_Cu∙2.5MeOH} [found (calcd.)]: C, 50.0% (50.06%); H, 3.9% (4.32%); N, 13.2% (13.34%). ESI-MS *m/z* (+) 399.51 (calcd. for [C_17_H_12_N_4_O_2_Cu + MeOH + H]^+^ 400.06). FTIR (KBr pellet, cm^−1^) 3,440 (br), 3,342, 3,216, 3,036, 1,625, 1,602, 1,523, 1,493, 1,454. UV-Vis [DMSO, λ, nm (ε, M^−1^cm^−1^)] 291 (2.9 × 10^4^), 345 (sh, 1.55 × 10^4^), 417 (1.3 × 10^4^), 535 (br, 1.4 × 10^3^).
**[Cu**(**L** ^**8**^ **)]**_**3**_ (**8**)**:** Reddish-brown solid. Yield: 52.7%. Elem. analysis for 3×{C_16_H_10_N_4_O_2_Cu∙1.75H_2_O∙0.25MeOH} [found (calcd.)]: C, 49.5% (49.62%); H, 3.3% (3.72%); N, 14.0% (14.24%). ESI-MS *m/z* (+) 354.08 (calcd. for [C_16_H_10_N_4_O_2_Cu + H]^+^ 354.02). FTIR (KBr pellet, cm^−1^) 3,435 (br), 3,047, 1,628, 1,595, 1,521, 1,499, 1,460. UV-Vis [DMSO, λ, nm (ε, M^−1^cm^−1^)] 276 (1.1 × 10^4^), 340 (9.0 × 10^3^), 387 (6.0 × 10^3^), 495 (br, 1.6 × 10^3^).

## 3 Results and discussion

### 3.1 Synthesis and characterization

The organic compounds here described are benzohydrazide-hydrazone derivatives of 8-hydroxy-2-quinolinecarbaldehyde (8HQ-2CHO). The synthesis, solid state and solution characterization (including p*K*_a_ values) of L^1^ - L^6^, have already been included in a previous publication in which their binding to V(IV)O was also disclosed (Ribeiro et al., 2022). Compounds labelled L^7^ and L^8^ are related to the previously described compounds, corresponding to the new condensation products of 8HQ-2CHO with 4-aminobenzoic hydrazide and isoniazid, respectively. Their characterization will now be briefly described. The NMR spectra of both compounds were measured in DMSO_*d*_*6*_ and the chemical shifts are in good agreement with the other compounds of this family: the imine proton appears at 7.76 and 7.96 ppm for L^7^ and L^8^, respectively (Supplementary Table S2 in Supplementary Material, SM) and the corresponding carbons at 136.5 and 140.2 ppm. In both compounds, the proton from the hydroxyl group is present at 10.86 ppm, while the NH is at 15.50 ppm for L^7^ and 16.08 ppm for L^8^. The protons from the amino benzyl substituent in L^7^ resonate at 5.93 ppm.

Copper complexes are all new and were synthesized in moderate to good yields from the Cu(II) acetate salt. Elemental analysis and mass spectrometry reveal a 1:1 ligand-to-metal stoichiometric ratio for all. Interestingly, while with L^1^ (no substituent) and L^4^ (methyl substituted) an acetate anion from the original salt seems to be maintained in the solid-state complex, in all other complexes double deprotonation of the ligand is assumed. Regarding the substituent effects, unsubstituted or substituents with inductive charge effect, like CH_3_, do not seem to be enough to doubly deprotonate the ligand. The other substituents, having a resonance effect on the aromatic ring, allow for the double deprotonation of the ligand and the coordination to the Cu(II) centre in a di-anionic form. All complexes were spectroscopically characterized, however, and due to the large number of compounds synthesized, with some techniques only selected compounds will be evaluated.

The infra-red absorption spectra (FTIR, Supplementary Figure S1) of the two new ligand precursors are consistent with those already reported for the related compounds (Ribeiro et al., 2022), presenting narrow, strong bands in the region around 3,400 cm^−1^, assigned to OH and NH stretching vibrations. A set of bands between 1,680 and 1,500 cm^−1^, due to vibrations of double bonds in the carbonyl and imine moieties, as well as in the aromatic rings, is observed. Coordination to copper leads to the disappearance of the sharp OH and NH bands, with a broad band appearing in the same region, probably associated with hydrogen-bonded OH and NH groups and solvent molecules retained in the solid matrixes (Supplementary Figure S2 and Supplementary Table S3). Significant changes are also registered in the region 1,680 – 1,500 cm^−1^ upon metal binding, showing the involvement of both carbonyl and nitrogen atoms of amide, imine and quinoline moieties in the metal coordination.

All compounds were characterized by UV-Vis absorption spectroscopy in DMSO (Supplementary Table S3, Supplementary Figure S3, S4). The organic compounds, L^1^-L^8^, show electronic transitions typical for this type of compounds, namely, π→π* and n→π* transitions (Liu and Yang, 2009b; Ribeiro et al., 2022). Cu(II) complexes spectra show the presence of the same bands, although shifted, and new ones at higher wavelengths with lower molar extinction coefficients, consistent with charge transfer bands between the ligand and the metal centre.

### 3.2 Structural characterization by SCXRD

**FIGURE 1:**
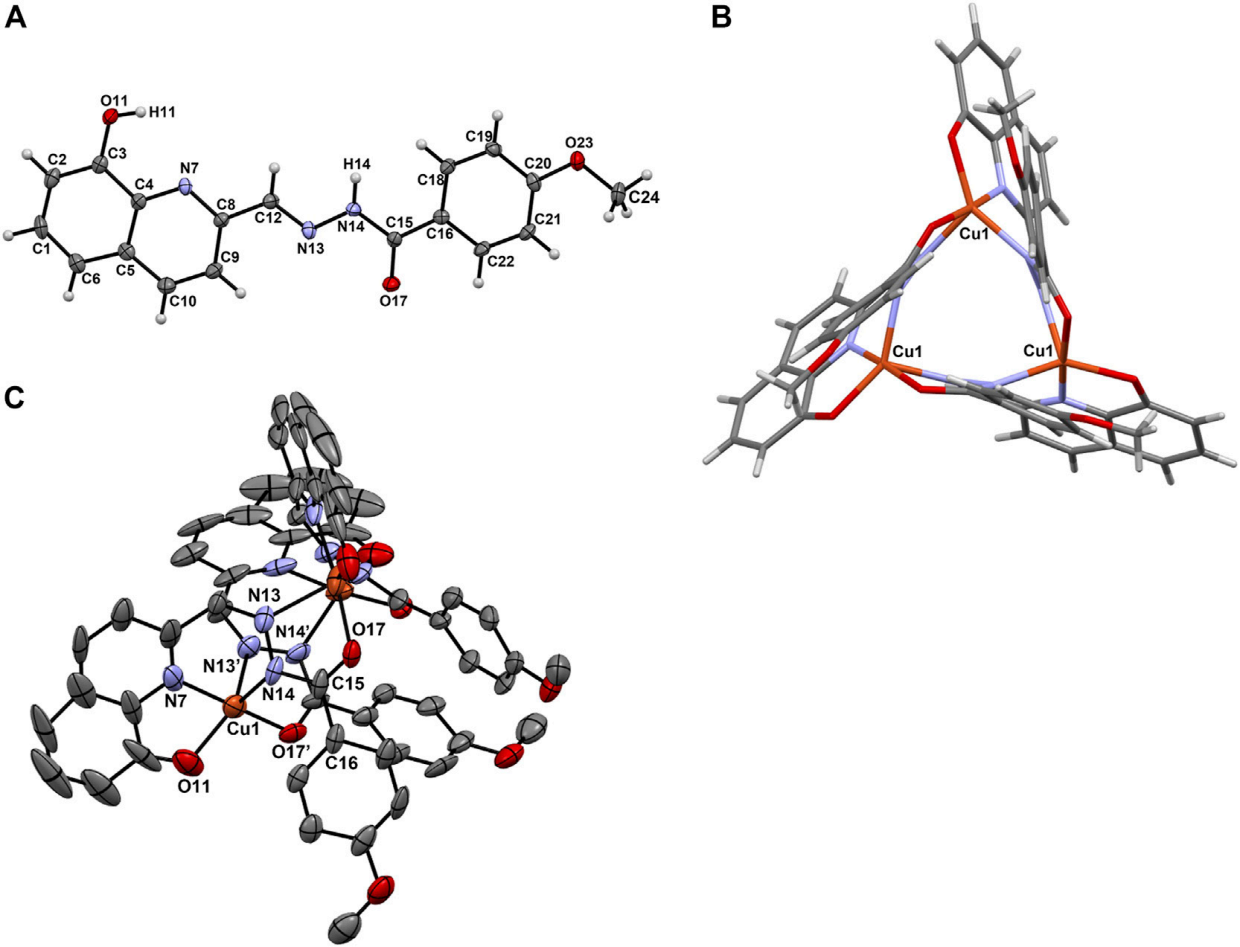
**(A)** ORTEP diagram of L^5^ with thermal ellipsoids at 40% probability level along with the atom numbering scheme; **(B)** View of the binding in the equilateral triangle formed between the Cu(II) centres in [Cu(L^5^)]_3_ (**5**) and **(C)** ORTEP diagram of [Cu(L^5^)]_3_ with thermal ellipsoids at 40% probability level. Hydrogen atoms are omitted for clarity.

Single crystals of L^5^ and its Cu-complex **5** were obtained from acetone and methanol, respectively. L^5^ crystallized in the triclinic system, space group P−1, and the asymmetric unit consists of two molecules of L^5^ and three water molecules engaged in hydrogen bonds with the protonated phenol-O and carbonyl-O atoms (Supplementary Table S4). The compound adopts an *E* configuration relative to the hydrazonic C12=N13 linkage (Figure 1A). The C8-C12-N13-N14 dihedral angle is 179.5(7)° in L^5^, while in the complex, after reorientation of the nitrogen in relation to the quinoline ring, twisting and bending of the molecule, it becomes 2.9(19)°. Complex **5** crystallizes as a trimer in the trigonal system, space group P−3. Each ligand acts as a dibasic pentadentate ligand being shared by two different pentacoordinated Cu(II) metal centres (Figure 1B). Each copper centre is surrounded by the following donors from two ligand molecules: [(O^−^, N, N^2^_im_); (N^1^_im_, CO^−^)]. Both carbonyl oxygen atoms are deprotonated since enolization and deprotonation of the amide group occur upon coordination, decreasing the C15-N14 bond by 0.02 Å and increasing the C15-O17 bond by 0.026 Å (Table 1) (Das et al., 2014). At the same time, the N14-C15-O17 angle increases by 5.3°, while the C16-C15-O17 and C15-N14-N13 angles decrease by 5.5° and 8.1°, respectively, due to the enolization and coordination of this part of the molecule. The three Cu(II) atoms form an equilateral triangle with a distance of 4.780(3) Å between metal centres (Figure 1C).

**TABLE 1:** Selected bond lengths (Å) and angles (°) for L^5^ and **[Cu**(**L** ^**5**^ **)]**_**3**_ (**5**). Atoms labelled with a prime (´) belong to a second ligand molecule coordinated to the same Cu(II) centre.

	L^5^	[Cu(L^5^)]_3_ (**5**)
Bond lengths (Å)		
C3-O11	1.347(10)	1.263(18)
C4-N7	1.359(9)	
C8-N7	1.323(12)	
C12-N13	1.286(13)	1.284(11)
N13-N14	1.381(10)	1.390(10)
C15-N14	1.359(13)	1.339(12)
C15-O17	1.236(10)	1.262(12)
Cu1-O11		1.959(8)
Cu1-N7		1.940(9)
Cu1-N14		2.010(10)
Cu1-N13′		2.204(8)
Cu1-O17′		1.956(6)
Bond angles (°)		
O11-C3-C4	120.5(8)	116.6(8)
C4-N7-C8	118.4(7)	121.9(9)
C12-N13-N14	116.0(7)	119.4(7)
C15-N14-N13	118.3(7)	110.2(8)
O17-C15-N14	120.1(7)	125.4(8)
O17-C15-C16	122.3(8)	116.8(8)
C16-C15-N14	117.6(7)	117.8(9)
O11-Cu1-N7		82.5(4)
N7-Cu1-N14		86.9(4)
N14-Cu1-N13′		95.0(4)
N13′-Cu1-O17′		76.0(3)
O17′-Cu1-O11		95.5(4)
O17′-Cu1-N7		173.1(4)
O17′-Cu1-N14		98.3(4)
O11-Cu1-N14		146.0(4)
O11-Cu1-N13′		118.5(4)

### 3.3 SQUID magnetometry

To gain further insight into the structure of the compounds in the solid-state, magnetization measurements of crystals of complex **5** were performed as a function of temperature (from 10 to 300 K, under an applied magnetic field of 0.1 T). Supplementary Figure S5 displays the thermal variation of the molar magnetic susceptibility, *χ*_m_ (= M/H, where M is the molar magnetization and H is the applied magnetic field), after diamagnetic contribution subtraction, as well as the product χ_m_T vs. temperature. The χ_m_T product is practically constant over all the temperature range, varying between 0.86 and 0.97 cm^3^ K mol^−1^. Assuming the spin-only model with *S* = ½ (*g* = 2) for each of three non-interacting Cu(II) ions, the value expected for χ_m_T is 1.25 cm^3^ K mol^−1^ for all spins aligned with the external applied magnetic field (↑↑↑) and 0.38 cm^3^ K mol^−1^ for a configuration of Cu(II) spins with total spin *S* = ½ (↑↓↑). The experimental result is far from these two values, indicating the need to consider interactions between the copper spins. Different approaches were considered to fit simultaneously *χ*_m_, *χ*_m_^−1^, χ_m_T vs. T curves, and program PHI (Chilton et al., 2013) with the isotropic Spin Hamiltonian:

$$\hat{H}=-2\left[J_{12}\left(\hat{S_{1}}∙\hat{S_{2}}\right)+J_{13}\left(\hat{S_{1}}∙\hat{S_{3}}\right)+J_{23}\left(\hat{S_{2}}∙\hat{S_{3}}\right)\right]+\mu_{B}\left(g_{1}\hat{S_{1}}+g_{2}\hat{S_{2}}+g_{3}\hat{S_{3}}\right)$$ (1)

The results indicate a rather weak interaction between the three *S* = ½ Cu(II) ions, either pointing to a weak isotropic parallel coupling (0<J<1) cm^−1^ (Supplementary Figure S5), or to a weak antiparallel one, which indicates the existence of magnetic frustration among the three Cu ions (−1 < *J* < 0) cm^−1^. The small value obtained for *J* can be explained by the indirect bonding between the Cu(II) ions.

Both spin configurations are consistent with three *S* = ½ Cu(II) ions at the same distance from each other, in equivalent local symmetry sites, in perfect agreement with the Cu equilateral triangle configuration obtained from crystallographic data.

### 3.4 EPR characterization

**FIGURE 2:**
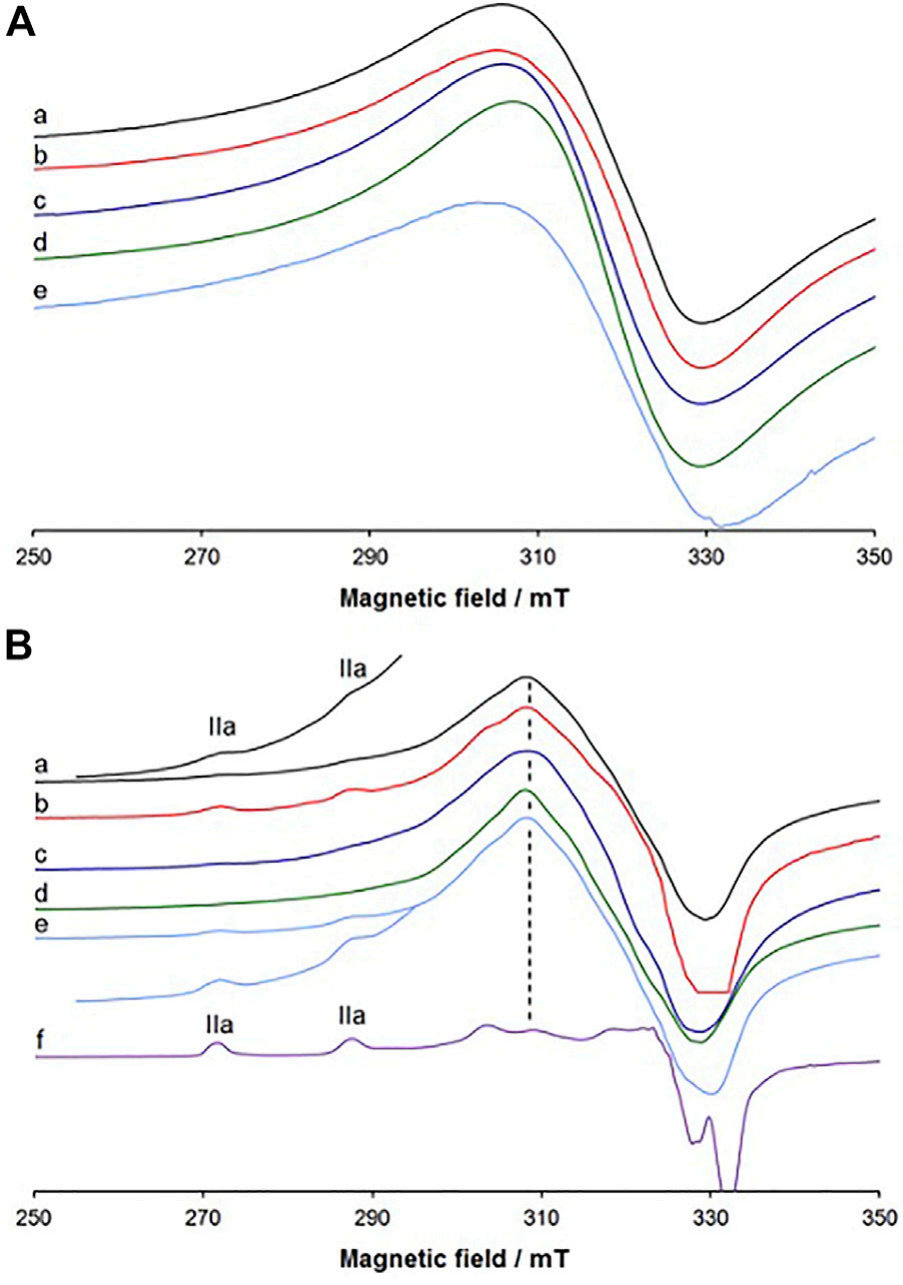
**(A)** X-band EPR spectra recorded at 120 K on: **(A)** polycrystalline samples of **1** (a), crystalline **5** (b), polycrystalline **5** (c), **6** (d) and **7** (e); **(B)** polycrystalline samples of **1** (a), **4** (b), polycrystalline **5** (c), crystalline **5** (d), and **6** (e) dissolved in DMSO. The region between 255.0 and 295.0 mT of the traces (a) and (e) is amplified by four and three times, respectively. In (f) the spectrum recorded in the system Cu(II)/H_2_L^4^ 1:1 at pH 6.50 in the mixture of 80% (v/v) DMSO/H_2_O is also shown. **IIa** indicates the first two parallel resonances of the complex [Cu(L)] with [(O^−^, N, N^2^_im_); DMSO] binding set, and the dashed line indicates the distinctive resonance of the trinuclear species [Cu(L)]_3_.

For selected complexes (**1**, **5**, **6** and **7**) electron paramagnetic resonance (EPR) spectra were recorded at 120 K on amorphous (polycrystalline) powder samples, and these are reported in Figure 2A. They exhibit a single isotropic feature with *g* values of 2.114, 2.118, 2.115 and 2.110, respectively. The spectrum recorded on the crystals of [Cu(L^5^)]_3_ (**5**) (with *g* = 2.112) is very similar to that collected on the polycrystalline powder of the same compound and this demonstrates that the two structures are comparable. The detected isotropic absorptions are usually the result of the dipolar interaction and intercentre exchange between neighbouring units that broaden the hyperfine lines (Rizzi et al., 2016) and support the trinuclear nature observed by SCXRD analysis. Notably, the same features have also been revealed for other trimeric Cu(II) complexes (Thakurta et al., 2008; Saha et al., 2014).

When the complexes are dissolved in DMSO, the spectral pattern does not change significantly and the signal is dominated by an almost and little resolved isotropic band (Figure 2B and Supplementary Figure S6). This suggests that the complexes partly keep their structure in the organic solution. However, in the parallel region, the hyperfine coupling between an unpaired electron and the Cu(II) nucleus is observed. The resonances of this mononuclear complex are indicated with **IIa** and coincide with those of the species that the spectrophotometric titrations interpreted as [Cu(L)] (see *infra*). Considering that in the X-ray structure of complex **5**, the L^5^ ligand adopts a trinuclear coordination with the donor set (O^−^, N, N^2^_im_), the resonances **IIa** could be assigned to a complex with equatorial coordination of [(O^−^, N, N^2^_im_); DMSO]. In other words, in DMSO and H_2_O the equilibrium (Eq. 2) is established:

$${\left[Cu\left(L\right)\right]}_{3}+3\;DMSO/H_{2}O\;⇄\;3\;\left[Cu\left(L\right)\left(DMSO/H_{2}O\right)\right]$$ (2)

In DMSO the two species [Cu(L)]_3_ and [Cu(L)(DMSO)] coexist (see Figure 2B), while in water the formation of [Cu(L)(H_2_O)] is favoured (see i*nfra*, section 3.7). The resonances at 309.4 mT, denoted by the dashed line in Figure 2B, can be considered distinctive of the presence in solution of the trinuclear complex [Cu(L)]_3_. The comparison between the behaviour of the crystalline and polycrystalline samples is highlighted in Supplementary Figure S7, which demonstrates that the two forms behave similarly according to reaction in Eq. 2.

It is worth noting that, in contrast with other reported polynuclear Cu(II) complexes, which in organic solvents give the mononuclear units (Alves et al., 2004; Ali et al., 2005; Koval et al., 2006; Pradeep and Das, 2009; Ray et al., 2010; Thakurta et al., 2010; Shit et al., 2013), in our [Cu(L)]_3_ complexes, the bridges are strong enough to survive to some extent in DMSO solution and are not broken; this is probably due to the nature of the bridge, disclosed by the SCXRD study, which shows a tridentate behaviour of the ligand L on one side, with the set (O^−^, N, N^2^_im_), and bidentate on the other side with the donor set (N^1^_im_, CO^−^). In aqueous solution we expect the predominance of the [Cu(L)(H_2_O)] species, as shown in the next section.

### 3.5 Proton dissociation processes and solubility of the ligand precursors

Investigation of the proton dissociation processes of a bioactive compound is necessary for the interpretation of the results of the biological assays since based on the p*K*_a_ values one can determine the actual chemical form and charge at a given pH. The p*K*_a_ values for the related 8-hydroxyquinoline-hydrazone conjugates (L^1-6^) were already determined in our previous work (Ribeiro et al., 2022) by UV-Vis spectrophotometric titrations in 30% (v/v) DMSO/H_2_O (Supplementary Table S5). For the reference compound L^1^ three p*K*_a_ values could be calculated based on the spectral changes, and p*K*_a1_, p*K*_a2_ and p*K*_a3_ were assigned to the deprotonation of the quinolinium NH^+^, the hydroxyl and the benzohydrazide NH moieties, respectively. The various substituents (Cl, F, CH_3_, OCH_3_) have negligible influence on these p*K*_a_ values. For L^6^ with the additional OH group also three p*K*_a_ values were obtained, although p*K*_a2_ and p*K*_a3_ were attributed to the two hydroxyl moieties, and the p*K*_a_ of the benzohydrazide NH was fairly high (>12) (Ribeiro et al., 2022).

Both L^7^ and L^8^ have one more dissociable proton than L^1^, namely, L^7^ has the ammonium group and L^8^ the pyridinium nitrogen, which may dissociate in the acidic pH range (ChemAxon, 2012). Three p*K*_a_ values could be calculated for L^7^ and L^8^ under the same experimental conditions and are collected in Table 2. Supplementary Figure S8 shows the spectra of L^8^ and the computed molar absorbance spectra of the ligand in the different protonation states. As the deprotonation of the ammonium (L^7^) and the pyridinium nitrogen (L^8^) are assumed to take place in the same pH range as the quinolinium NH^+^, it is not possible to assign the p*K*_a1_ to a particular moiety, while p*K*_a2_ and p*K*_a3_ undoubtedly belong to the hydroxyl and the benzohydrazide NH groups, respectively. Despite the relatively small differences found, L^7^ and L^8^ are also present in solution in their neutral H_2_L form at physiological pH, similarly to ligands L^1^-L^5^ (Ribeiro et al., 2022), as the concentration distribution curves show in Supplementary Figure S9.

**TABLE 2:** p*K*_a_ values of the studied ligand precursors determined by UV-Vis spectrophotometric titrations in 30% (v/v) DMSO/H_2_O in addition to their thermodynamic solubility (*S*_7.4_). [t = 25.0°C; I = 0.1 M (KCl)].

	L^7^	L^8^	L^1^ ^a^
R =	**NH**_**2**_	**H, X = N**	**H**
**p*K***_***a*1**_	2.37 ± 0.01	2.61 ± 0.01	2.24
**p*K***_***a*2**_	9.41 ± 0.01	8.95 ± 0.01	9.51
**p*K***_***a*3**_	12.25 ± 0.01	11.14 ± 0.01	11.45
***S***_**7.4**_ (μM)	1.4 ± 0.1	2.1 ± 0.2	3.7

^a^ Data taken from Ref. (Ribeiro et al., 2022).

The thermodynamic solubility (*S*) of both ligand precursors was also determined at pH 7.4 in water at 0.1 M KCl ionic strength (Table 2) revealing low values, as observed for the rest of the ligand series and a minor impact of the derivatization.

### 3.6 Solution stability of Cu(II) complexes formed with selected ligand precursors

**FIGURE 3:**
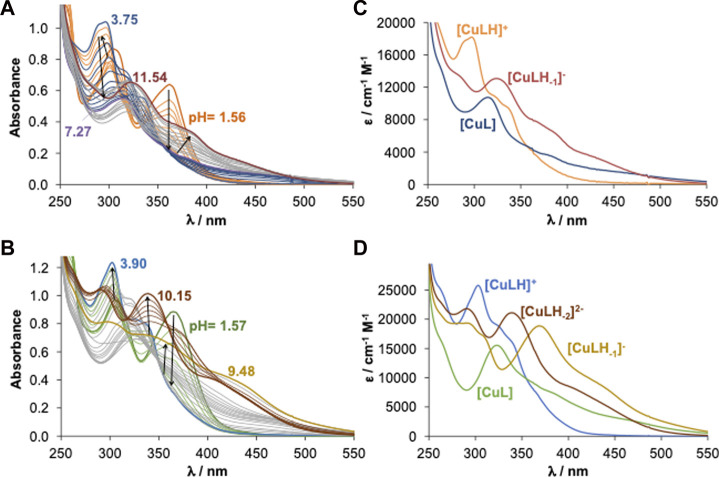
UV-Vis spectra of the **(A)** Cu(II) ‒ L^1^ (1:1) and **(B)** Cu(II) ‒ L^6^ (1:1) systems recorded at various pH values. Calculated spectra (extinction coefficients) of individual species of the **(C)** L^1^ and **(D)** L^6^ complexes. [*c*_Cu(II)_ = *c*_L_ = 50 μM; pH = 1.5–11.0; *t* = 25.0°C; *I* = 0.10 M (KCl); *ℓ* = 1.0 cm; 30% (v/v) DMSO/H_2_O].

The complex formation processes of L^1^, L^5^ and L^6^ with Cu(II) ions were studied by UV-Vis spectrophotometry in 30% (v/v) DMSO/H_2_O using relatively low concentrations (*c*_L_ = 50–60 μM), due to the limited water solubility of the ligand precursors as well as of the Cu(II) complexes (see thermodynamic solubility data in aqueous media in Table 2 and Table 3). L^1^ was selected as the simplest compound of the studied ligand series, whereas L^5^ and L^6^ were selected to compare with the oxidovanadium(IV) system (Ribeiro et al., 2022). The solution stability and stoichiometry of the Cu(II) complexes were determined by the evaluation of the UV-Vis spectra recorded at various pH values (representative spectra are shown for the Cu(II) ‒ L^1^ and Cu(II) ‒ L^6^ systems in Figures 3A,B and for Cu(II) ‒ L^5^ in Supplementary Figure S10A). The computed overall stability constants and p*K*_a_ values of the complexes are collected in Table 3. The formation of only mono-ligand complexes was found with [Cu(LH)]^+^, [Cu(L)], and [Cu(LH_‒1_)]^‒^ (and [Cu(LH_‒2_)]^2‒^ in the case of L^6^) compositions, and their computed molar absorbance spectra are shown in Figures 3C, D, Supplementary Figure S10B. The differences found in the intra-ligand and charge-transfer bands of these species suggest several different coordination modes; however, based on these spectra, it is difficult to judge the actual binding types, thus, EPR spectroscopic measurements were also done (*vide infra*).

**TABLE 3:** Overall (log*β*) stability and proton dissociation (p*K*_a_) constants of the Cu(II) complexes formed with L^1^, L^5^ and L^6^ determined by UV-Vis spectrophotometric titrations in 30% (v/v) DMSO/H_2_O in addition to their thermodynamic solubility (*S*_7.4_) and distribution coefficients (log*D*_7.4_) at pH 7.40 (these determined experimentally *via n*-octanol/water partitioning). [t = 25.0°C; I = 0.10 M (KCl)]. pCu (= −log[Cu(II)]) values were computed with the use of the stability constants at pH 7.4, at 1 μM Cu(II) and i) 1 μM or ii) 10 μM ligand concentrations. Formal potential values (*E*_1/2_) vs NHE and peak separations (Δ*E*) measured for the Cu(II) complexes by cyclic voltammetry [see the conditions in the legend of Supplementary Figure S14].

	L^1^	L^5^	L^6^
R =	H	OCH_3_	OH
log*β* [Cu(LH)]^+^	23.41 ± 0.02	22.75 ± 0.05	16.91 ± 0.03
log*β* [Cu(L)]	17.79 ± 0.02	18.26 ± 0.04	11.67 ± 0.03
log*β* [Cu(LH_‒1_)]^‒^	7.39 ± 0.03	7.18 ± 0.04	2.50 ± 0.03
log*β* [Cu(LH_‒2_)]^2‒^	−	−	−7.40 ± 0.04
p*K*_a_ [Cu(LH)]^+^	5.62	4.49	5.24
p*K*_a_ [Cu(L)]	10.40	11.08	9.17
pCu ^a^	8.96	9.03	7.26
pCu ^b^	12.59	12.69	9.44
*S*_7.4_ (μM) ^c^	5.8 ± 0.3	<1	1.3 ± 0.1
log*D*_7.4_	+1.9 ± 0.1	+2.4 ± 0.1	+2.0 ± 0.1
*E*_1/2_ (mV)	+395	+377	+380
Δ*E* (mV)	166	144	150

^a^ *c*_Cu(II)_ = 1 μM; *c*_L_ = 1 μM.

^b^ *c*_Cu(II)_ = 1 μM; *c*_L_ = 10 μM.

^c^ *S*_7.4_ determined for complex **2**: <1 μM; **3**: <1 μM; **4**: 4.0 ± 0.2; **7**: 4.2 ± 0.1; **8**: 3.3 ± 0.3.

In the [Cu(LH)]^+^ species most probably the ligand coordinates *via* the (O^−^, N) donor set of the 8HQ scaffold, while the benzohydrazide NH moiety is still protonated. Considering the much lower p*K*_a_ value of [Cu(LH)]^+^ (Table 3) than that of the benzohydrazide NH of the ligand precursors (p*K*_a3_ in the case of L^1^, L^5^, and p*K*_a4_ in the case of L^6^), upon the deprotonation of [Cu(LH)]^+^ the binding of the benzohydrazide N^2^_im_ donor atom is highly probable in the species [Cu(L)], adding to (O^−^, N) donor set, also in agreement with the SCXRD structure that showed one of the ligands assuming this binding mode. It is worth noting that an equilibrium between [Cu(L)] and the trinuclear complex [Cu(L)]_3_ (found by X-ray diffraction analysis, SQUID magnetometry and EPR spectroscopy) is possible; however, under the conditions used for the UV-Vis titrations (very low concentration, presence of the weakly coordinating DMSO), the percent amount of polynuclear species is assumed to be rather low. [Cu(LH_‒1_)]^‒^ is most likely a mixed hydroxido complex (=[Cu(L)(OH)]^‒^ with [(O^−^, N, N^2^_im_); OH^−^] binding mode) in the case of L^1^ and L^5^, while it is formed by the deprotonation of the additional OH group of L^6^ based on the much lower p*K*_a_ of [Cu(L)]. Additionally, the development of a new band at 368 nm upon the formation of [Cu(LH_‒1_)]^‒^ from [Cu(L)] also supports this suggestion (Figure 3D), which is not present in the case of L^1^ and L^5^. Formation of [Cu(LH_‒2_)]^2‒^ was only found with L^6^ and this species is suggested to be a mixed hydroxido species.

**FIGURE 4:**
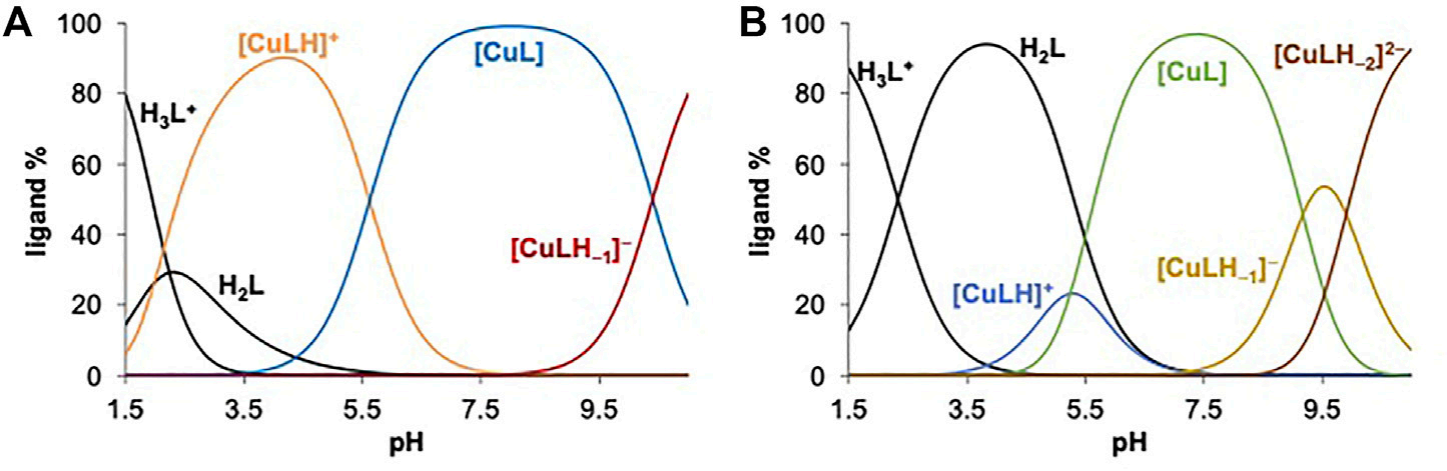
Concentration distribution curves calculated for the **(A)** Cu(II) ‒ L^**1**^ (1:1) and **(B)** Cu(II) ‒ L^6^ (1:1) systems. [*c*_Cu(II)_ = *c*_L_ = 50 μM; *t* = 25.0°C; *I* = 0.10 M (KCl); 30% (v/v) DMSO/H_2_O].

Concentration distribution curves (Figure 4) show that [Cu(L)] species predominate at physiological pH. To compare the Cu(II) binding ability of the studied ligands, the pCu (= ‒log [Cu(II)]) was computed at 1 μM metal ion concentration, and 1 and 10 μM ligand concentrations at pH 7.4, based on the obtained stability constants (Table 3). The higher pCu indicates stronger metal ion binding under the given conditions. These values reflect the similar Cu(II)-binding strength of L^1^ and L^5^, whereas L^6^ forms somewhat lower stability complexes in comparison to the other two ligands, which suggests that the benzohydrazide N^2^_im_ donor is not involved in the coordination in complex **6**. These Cu(II) complexes are the most stable species at physiological pH, with a level of dissociation at 1 μM concentration corresponding to only ca. 5% for complex **6**.

As the complex formation of ligands L^4^ and L^6^ was also studied with oxidovanadium(IV) the speciation data obtained here for the Cu(II) complexes were compared and detailed in the SI (Supplementary Figure S11B and its legend). It was pointed out that the binding of these ligands to Cu(II) at pH > 7 is much stronger than to oxidovanadium(IV).

### 3.7 EPR spectra in solution

The Cu(II) binding modes in solution were studied by EPR spectroscopy at 120 K in a mixture of 80% (v/v) DMSO/H_2_O in the systems containing L^4^ or L^6^ with molar ratio 1:1.

**FIGURE 5:**
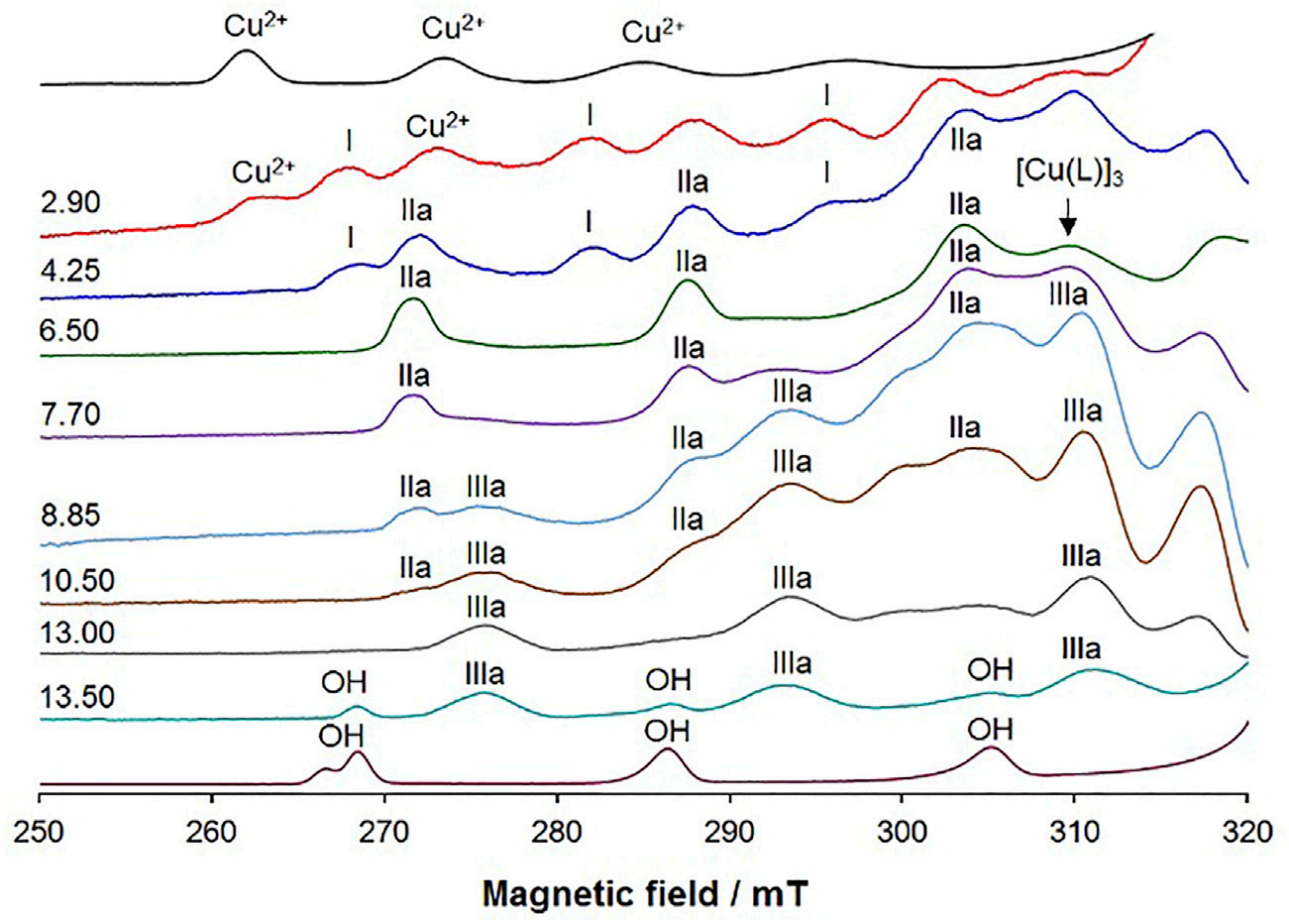
Low field region of the X-band anisotropic EPR spectra recorded as a function of pH at 120 K in a mixture DMSO/H_2_O 80/20 (v/v) containing ^63^Cu(II) and L^6^ (molar ratio 1:1 and Cu(II) concentration 1.0 mM). The first and last spectra were recorded on solutions containing the solvated and hydroxide complexes. With **I** are indicated the first three equatorial resonances of [Cu(LH)]^+^, with **IIa** of [Cu(L)] with the donor set [(O^−^, N, N^2^_im_); H_2_O], and with **IIIa** of [Cu(LH_–1_)]^–^ with the donor set [(O^−^, N, N^2^_im_); OH^−^]. With **Cu** ^**2+**^ and **OH** are denoted the resonances of solvated Cu^2+^ ion and hydroxo [Cu(OH)_4_]^2–^ complexes, taken as references. Moreover, the position of the resonance distinctive of the trinuclear complex [Cu(L)]_3_ is also indicated by the arrow.

The spectra measured with L^6^ are depicted in Figure 5, while the spin Hamiltonian parameters are listed in Table 4. The complexation starts with the formation of [Cu(LH)]^+^ with the coordination of 8-hydroxyquinoline moiety and [(O^−^, N); H_2_O; H_2_O] equatorial donor set. Its first three parallel resonances are indicated with **I** in Figure 5.

**TABLE 4:** Experimental spin Hamiltonian parameters for Cu(II) complexes formed by **L^6^** and **L^4^**.

Ligand	Species	*g*_z_	|*A*_z_| (^63^Cu)^a^	Equatorial donor set
**L** ^**6**^ (R = OH)	[Cu(LH)]^+^ (**I**)	2.325	152.3	[(O^−^, N); H_2_O; H_2_O]
	[Cu(L)] (**IIa**)	2.273	169.5	[(O^−^, N, N^2^_im_); H_2_O]
	[Cu(LH_–1_)]^–^ (**IIIa**)	2.223	182.0	[(O^−^, N, N^2^_im_); OH^−^]
**L** ^**4**^ (R = CH_3_)	[Cu(LH)]^+^ (**I**)	2.326	150.7	[(O^−^, N); H_2_O; H_2_O]
	[Cu(L)] (**IIa**)	2.273	170.0	[(O^−^, N, N^2^_im_); H_2_O]
	[Cu(L)] (**IIb**)	2.217	183.6	[(O^−^, N, N^1^_im_); H_2_O] or [(O^−^, N, N^1^_im_, CO^−^)]
	[Cu(LH_–1_)]^–^ (**IIIa**)	2.224	183.8	[(O^−^, N, N^2^_im_); OH^−^]
	[Cu(LH_–1_)]^–^ (**IIIb**)	2.296	∼174	[(O^−^, N, N^1^_im_); OH^−^]

^a^ Hyperfine coupling constant reported in 10^−4^ cm^–1^ units.

Upon increasing the pH, another complex is observed (**IIa** in Figure 5) with *g*_z_ = 2.273 and |*A*_z_| = 169.5 × 10^−4^ cm^–1^. For this species, whose composition is [Cu(L)] according to the spectrophotometric data, several coordination modes could be possible: [(O^−^, N, N^2^_im_); H_2_O] [(O^−^, N, N^1^_im_); H_2_O] or [(O^−^, N, N^1^_im_, CO^−^)]. The values of Δ*G*_aq_^calcd^ are −9.0 kcal mol^–1^, –2.3 kcal mol^–1^, and –3.4 kcal mol^–1^ (Supplementary Figure S12), respectively, suggesting that the donor set [(O^−^, N, N^2^_im_); H_2_O] is the most stable since it shows the lowest value of Δ*G*_aq_^calcd^. This is in agreement with the SCXRD structure that suggests that each Cu(II) ion is bound to the same donors from one molecule, and with the EPR spectra collected after dissolving the solid compounds in DMSO solution (see Figure 2). Moreover, the tridentate binding (O^−^, N, N^2^_im_) is the only one that makes possible the bidentate coordination (N^1^_im_, CO^−^) to an adjacent [Cu(L)] unit. It must be noted that [Cu(L)] predominates in solution from pH 6 to pH 8, in line with the distribution curves in Figure 4; furthermore, in the pH range of existence of [Cu(L)] the distinctive resonance of [Cu(L)]_3_ appears (Figure 5), indicating that equilibrium in Eq. 2 happens in DMSO/H_2_O mixtures as well (but is shifted towards the right), and that the species [Cu(L)] (with a water molecule that occupies the fourth metal equatorial site) and [Cu(L)]_3_ coexist in solution. As equilibrium in Eq. 2 is not pH dependent and due to the low solubility of the complexes, individual characterization of [Cu(L)] and [Cu(L)]_3_ by spectrophotometry is difficult (see Section 3.6).

The formation of the complex [Cu(L)] with coordination [(O^−^, N, N^2^_im_); H_2_O] allows also accounting for the appearance of another species at pH higher than 8.0 (**III** in Figure 5), to which the UV-Vis spectrophotometric titrations assign the stoichiometry [Cu(LH_–1_)]^–^. It has *g*_z_ = 2.223 and |*A*_z_| = 182.0 × 10^−4^ cm^–1^. It is plausible that this latter species is originated from [Cu(L)] upon the deprotonation of an equatorial water molecule to give the binding mode [(O^−^, N, N^2^_im_); OH^−^]; the coordination of an OH^−^ in the equatorial plane of Cu(II) ion, which replaces a water ligand, agrees well with the decrease of *g*_z_ and the increase of *A*_z_.

Finally, the deprotonation with a p*K*_a_ of 9.90, suggested by spectrophotometry, can be ascribed to the non-coordinating phenolic–OH on the phenyl ring; since this deprotonation does not change the coordination mode, the spin Hamiltonian EPR parameters remain the same up to the formation of the hydroxo complex [Cu(OH)_4_]^2–^, denoted with **OH** in Figure 5, that appears in solution at pH > 13.

The behaviour of the system with L^4^ is shown in Supplementary Figure S13. The first observed resonances, after the solvated Cu^2+^ ion, are those indicated by **I**. The EPR parameters are comparable to those of the similar species detected with L^6^ and the same equatorial coordination mode, [(O^−^, N); H_2_O; H_2_O], is assigned (Table 4). This corresponds to [Cu(LH)]^+^, determined also by spectrophotometric titrations.

Upon increasing the pH to ∼4, two new species appear in solution, indicated by **IIa** and **IIb** in Supplementary Figure S13, which are the major species in solution, from pH 5.5 to 8.5. According to the concentration distribution curves in Figure 4, they should correspond to [Cu(L)]. The fact that the intensity ratio of the two sets of resonances remains unaltered in a wide pH range suggests that they could be isomers with the ligand in the same protonation degree and a different coordination mode around Cu(II) ion. Species **IIa** is characterized by spin Hamiltonian parameters very close to the similar species detected in the system with L^6^ and after the dissolution of the solid complexes: *g*_z_ is 2.273 and |*A*_z_| = 170.0 × 10^−4^ cm^–1^; thus, an identical donor set [(O^−^, N, N^2^_im_); H_2_O], is assigned. The species **IIb** has *g*_z_ = 2.217 and |*A*_z_| = 183.6 × 10^−4^ cm^–1^. For this species, the superhyperfine coupling with ^14^N is detected; the number of lines (five) with a ratio 1:2:3:2:1 and a mean coupling constant *A*_z_ (^14^N) of 14.8 × 10^−4^ cm^–1^ indicate the binding of two nitrogen atoms to copper (Xu and Chen, 1996; Klement et al., 1999; Thakurta et al., 2008). For **IIb** two donor sets are possible: the ligand in a tetradentate mode with [(O^−^, N, N^1^_im_, CO^−^)] or in a tridentate mode with the binding set [(O^−^, N, N^1^_im_); H_2_O] without the coordination of the negative CO^−^ donor group. Moreover, it must be observed that, in the pH range 5.5–8.5, the presence of a minor amount of [Cu(L)]_3_ in equilibrium with [Cu(L)] cannot be excluded (Supplementary Figure S13).

Above pH 10, two new sets of resonances are revealed, indicated by **IIIa** and **IIIb** (Supplementary Figure S13). **IIIa** has *g*_z_ = 2.224 and |*A*_z_| = 183.8 × 10^−4^ cm^–1^ and is assigned to [Cu(LH_–1_)]^–^, found by spectrophotometric titrations. Its coordination set is proposed as [(O^−^, N, N^2^_im_); OH^−^], similarly to what was detected with L^6^. **IIIb** coexists with **IIIa**, and its detection could be explained by the transformation of [Cu(L)] with [(O^−^, N, N^1^_im_); H_2_O] donor set to [Cu(LH_–1_)]^–^ with [(O^−^, N, N^1^_im_); OH^−^]. At pH > 12, the resonances of [Cu(OH)_4_]^2–^ (**OH**) are observed.

Overall, both spectroscopic techniques agree concerning the pH speciation of the system. EPR also evidences the coexistence of mononuclear and trinuclear species, which spectrophotometric techniques are unable to disclose due to the similar stoichiometry of both. At physiological pH the complexes are in the neutral form [Cu(L)] and the coordination probably involves the tridentate ligand with the donor atoms (O^−^, N, N^2^_im_).

### 3.8 Redox properties of selected Cu(II) complexes

Copper is a redox-active metal that can react with intracellular reducing agents and induce the formation of ROS. These processes may change the redox state of the cell and impact several important metabolic and signalling processes, which in the end may lead to cell death. The redox properties of selected compounds (**1**, **5** and **6)** were investigated by cyclic voltammetry in 9:1 (v/v) DMSO:HEPES (0.01 M, pH 7.4) with 0.1 M TBAN background electrolyte. These conditions were chosen to provide sufficient solubility of the complexes (1 mM), even though the pH scaling differs from that used for the UV-Vis titrations. Normalized cyclic voltammograms are shown in Supplementary Figure S14, and the electrochemical data are collected in Table 3. Both cathodic and anodic peaks could be detected; the peak separation was fairly large indicating irreversible electrochemical processes. The formal redox potentials obtained for the complexes fall in the range 377–395 mV vs. NHE which is higher than the reported redox potentials of the GSSG/GSH (−260 mV) (Schafer and Buettner, 2001) and dehydro-L-ascorbic acid/AA (+50 mV) (Hartinger et al., 2006) redox pairs.

**FIGURE 6:**
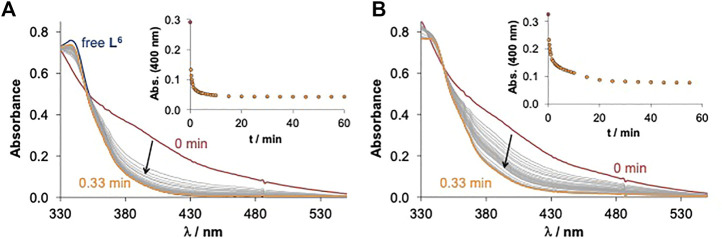
Time-dependent changes of the UV-Vis spectra of the Cu(II) complex **6** (40 µM) in the presence of 100 equiv. **(A)** GSH (4 mM) and **(B)** AA (4 mM) at pH 7.4 in 5% (v/v) DMSO/H_2_O under anaerobic conditions. Inserted figures show the changes in absorbance values plotted against time at 400 nm [t = 25.0°C; I = 0.10 M (KCl)].

The direct redox reaction between the copper complexes and GSH and AA was monitored spectrophotometrically under anaerobic conditions at pH 7.4 in the presence of 5% (v/v) DMSO using a tandem cuvette. After the addition of both reducing agents (100 equiv. since a high excess is feasible under physiologically relevant conditions) to the Cu(II) complexes significant spectral changes were observed, and the redox reactions reached the equilibrium within 20 or 60 min for GSH and AA, respectively (Figure 6). These indicate the liberation of the free ligand during the redox reaction since most probably the generated Cu(I) complex dissociates and a stable Cu(I) complex is formed with GSH (or AA), which is present in a high excess when compared to the 8HQ-based ligand. The recorded absorbance−time curves were further analysed and changes were compared (Supplementary Figure S15). Our description of these kinetic runs is only semi-quantitative; however, it can give information about differences in reaction rates. Observed rate constants (*k*_obs_) were calculated as the slope of the ln (A/A_0_) vs. time plots (see an example in Supplementary Figure S16 for the reaction of complex **5** with GSH) and the calculated values are collected in Supplementary Figure S15. It can be concluded that the redox reaction is faster with GSH in all cases and the reaction rates for the complexes gave the following trend: **1** ∼ **6** > **5**.

### 3.9 Stability in aqueous media

UV-Vis absorption spectra of the compounds were measured in an aqueous buffer (HEPES, 0.01 M, 0.1 M KCl, pH 7.4) by dilution of DMSO stock solutions (Supplementary Figures S17, S18). The complexes showed no decomposition or hydrolysis but simply a decrease in the intensity of the absorption bands, due to precipitation.

### 3.10 Binding to macromolecules

The analysis of the interaction of newly synthesized compounds with bovine serum albumin (BSA) is the first model in the evaluation of their transport in blood, upon administration. It was monitored by studying the quenching of albumin’s fluorescence emission with increasing concentrations of the complexes (Carter and Ho, 1994). The Stern–Volmer analysis (see Supplementary Figure S19 and Supplementary Table S6) confirmed the formation of a ground state metal complex–protein adduct. Overall, data showed moderate to strong interaction between BSA and the synthesized complexes.

The ability to interact with DNA was studied for selected compounds with different techniques, which are detailed in SM (see Supplementary Figures S20–S23 and Supplementary Table S7): DNA thermal denaturation and DNA-ethidium bromide fluorescence competition assays. Overall, the results point to weak interactions of all compounds with DNA and, most likely, occurring at the external surface of the biomolecule.

To further investigate the nature of the interaction between the complexes and DNA, cell-free DNA binding assays followed by gel electrophoresis were performed. The ligand precursors had little to no effect on DNA migration except L^7^ (Supplementary Figure S24A). On the other hand, incubation of plasmid DNA with complexes **1**-**8** confirmed binding to DNA and changed the supercoiling status by increasing the nicked circular form. The most dramatic response was obtained with **1**, with the highest nicking efficiency. To understand whether the induction of DNA nicks requires ROS, the samples were pre-incubated with a singlet oxygen scavenger, NaN_3_. The change in DNA migration was reversed, suggesting that singlet oxygen is involved in the induced DNA nicking by the complexes. Overall, complexes **1-8** appear to be promising species with little activity of their corresponding ligands.

### 3.11 Anticancer activity of the compounds

Copper compounds were tested, along with the free ligands and cisplatin as the positive control, for their cytotoxicity on both melanoma (A-375) and lung adenocarcinoma (A-549) cell lines in the concentration range 0.78–50 μM. Following 72 h of incubation, cell viability was measured *via* sulforhodamine B (SRB) assay (Supplementary Figure S25). Compared to their free ligand counterparts, all complexes proved to be more effective/cytotoxic in both A-375 and A-549 cells, with all complexes, except **6**, exhibiting IC_50_ values <10 μM in both cell lines (Table 5, Supplementary Figure S25). Moreover, all selected Cu(II)-complexes showed higher cytotoxicity than the positive control cisplatin in both cells (except **6**). Therefore, the treatment of cells with copper compounds resulted in cell survival loss, albeit with varying efficacy.

**TABLE 5:** IC_50_ values of selected copper complexes 1–8, free ligands (L) and cisplatin (µM ± SD) on A-375 and A-549 cell lines.

IC_50_ (µM ± SD)							
A-375				A-549			
**1**	2.7 ± 0.03	L^1^	12.3 ± 0.5	**1**	4.2 ± 0.2	L^1^	>50
**2**	6.5 ± 0.8	L^2^	>50	**2**	9.3 ± 0.4	L^2^	>50
**3**	5.1 ± 0.3	L^3^	23.9 ± 1.5	**3**	8.9 ± 0.2	L^3^	>50
**4**	2.0 ± 1.3	L^4^	>50	**4**	3.9 ± 0.6	L^4^	>50
**5**	3.4 ± 0.1	L^5^	30.9 ± 0.3	**5**	5.1 ± 0.01	L^5^	>50
**6**	13.6 ± 0.9	L^6^	21.2 ± 0.2	**6**	16.0 ± 0.3	L^6^	46.9 ± 0.8
**7**	5.1 ± 0.4	L^7^	12.4 ± 0.2	**7**	9.3 ± 0.2	L^7^	23.6 ± 0.2
**8**	2.8 ± 0.4	L^8^	9.1 ± 1.9	**8**	5.1 ± 0.2	L^8^	45.1 ± 2.7
cisplatin	11.2 ± 0.3			cisplatin	16.5 ± 3.4		

We have previously shown that all the oxidovanadium(IV) complexes of the same ligands (L^1^-L^6^) were also cytotoxic at µM doses (Ribeiro et al., 2022). Since the V(IV)O complex of L^4^ was not very effective for the induction of apoptosis in our previous analysis, we did not proceed further with **4**. Additionally, complex **7** does not have a V-counterpart, and its ligand is quite cytotoxic, so we excluded it from further studies and kept complex **8,** which has lower IC_50_ values and a ligand which presents much lower activity in A-549 cells. Complex **1** was the one that showed the highest ability to target DNA (highest ΔT_m_ and nicking efficiency) and **3** was selected to compare the metal effect since the vanadium complex of ligand L^3^ was the most effective in the melanoma cell line (Ribeiro et al., 2022). Additional reasons to choose **5** and **8** are the presence of groups with H-acceptor ability that seem to present better cytotoxic profiles than H-donor groups. Therefore compounds **1, 3, 5** and **8** were selected for additional studies.

### 3.12 Formation of reactive oxygen species and DNA double-strand breaks

**FIGURE 7:**
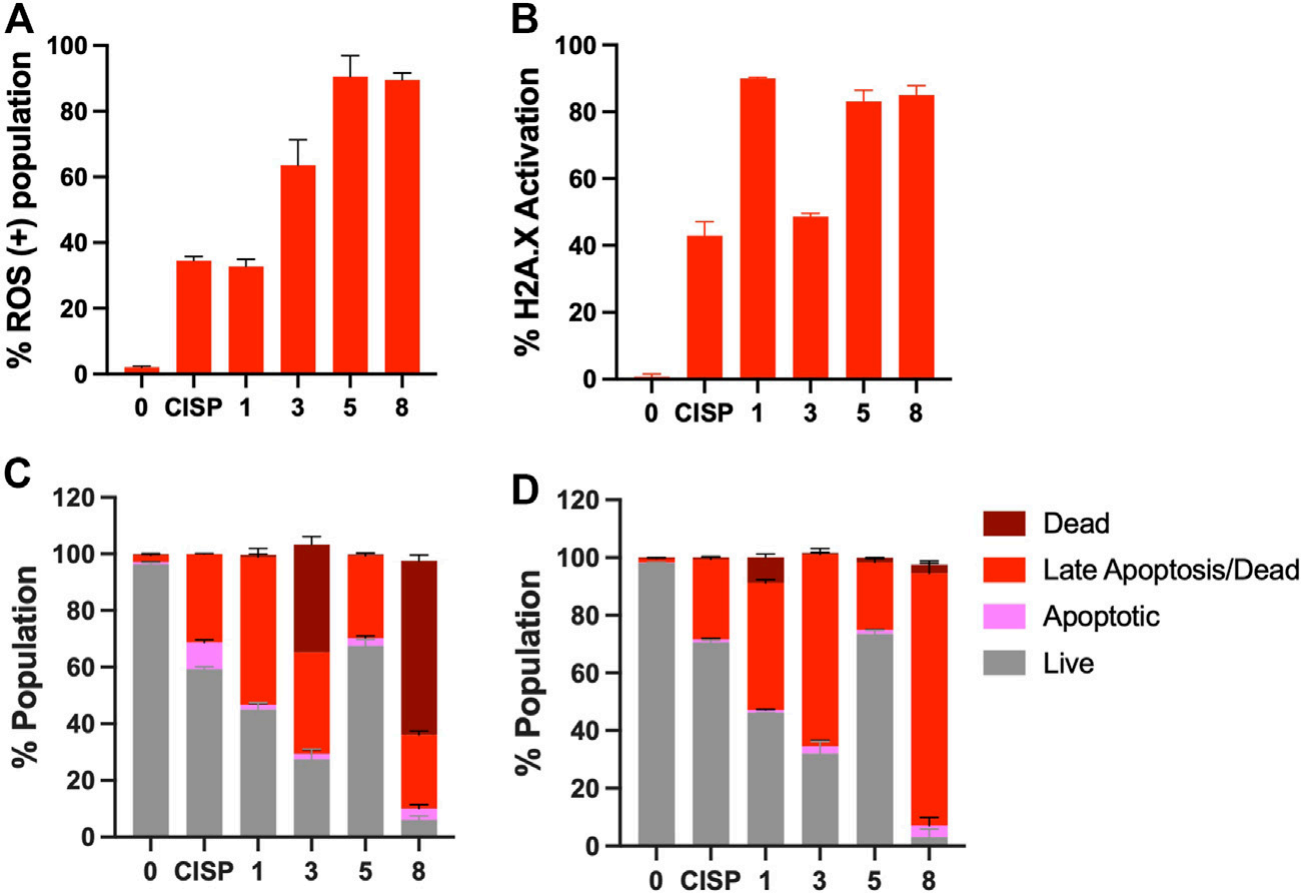
Induction of oxidative stress and DNA double strand breaks upon treatment with Cu(II) complexes. **(A)** A-375 cells were treated with cisplatin (CISP) or **1**, **3**, **5**, **8** and assessed for ROS activation *via* dihydroethidium staining. **(B)** A-375 cells were stained with antiphospho-Histone H2A.X (Ser139), Alexa Fluor^®^ 555 and anti-Histone H2A.X, PECy5 conjugated antibodies, following cisplatin or **1**, **3**, **5**, **8** treatment. The cells were scored using Muse Cell Analyzer. Induction of apoptosis upon treatment with the Cu-complexes. Flow cytometric analyses of **(C)** Annexin V/7-AAD positivity and **(D)** Caspase 3/7 activity on A-375 cells. Cells were exposed to **1, 3, 5** and **8** or cisplatin at half inhibitory concentrations for 48 h and counted with Muse Cell Analyzer.

Since reactive oxygen species appeared to be involved in the nicking activity of the complexes (Supplementary Figure S24), we investigated whether there was an increase in ROS formation in cells upon drug treatment. Dihydroethidium (DHE) is a reduced form of ethidium, after oxidation with superoxide (ROS), it is converted into 2-hydroxy ethidium, which is a chemical that emits red fluorescence and intercalates DNA. Not surprisingly, there was an induction of ROS upon treatment with all complexes under study at levels more than or at least comparable to cisplatin in A-375 cells (Figure 7A). Similar results were obtained using A-549 cells, but with much milder induction of ROS except for **8**, which remained high (Supplementary Figure S26). Complex **5** was the one which produced the milder ROS induction in these cells.

Additionally, to assess whether the ROS induction contributed to the cytotoxicity triggered by these complexes, we used a ROS scavenger, N-acetyl-cysteine (NAC), in combination with **1**, **3**, **5** or **8**. Our data showed that among the tested compounds, the cytotoxicity of **1**, **3**, and **8**, but not **5** was significantly reduced upon addition of NAC to the cell culture (Supplementary Figure S27), in both cell lines. Interestingly, NAC is known to scavenge some types of ROS (it directly interacts with ^•^OH and HOCl), but not others (i.e., H_2_O_2_, and O_2_^•−^). Therefore, this data suggested that while **1**, **3** and **8** potentially act through generation of ^•^OH and HOCl, **5** seems to act through other intermediates. Indeed, it is also possible that the cytotoxicity induced by **5** is not by ROS, despite the induction observed in Figure 7A.

Indeed, we examined whether DNA nicks induced by the complexes on pure plasmid DNA would reflect in cell culture as increased DNA break formation. To test this, A-375 cells were stained using γH2AX, which is a common marker for DSBs (Figure 7B). Mostly in accordance with ROS levels, cells were positive for γH2AX foci and **1**, **5** and **8** exhibited the highest levels of DSB induction. Interestingly, while **3** was able to induce a significant amount of ROS (60% positivity), the amount of DNA breaks did not correlate with the ROS levels, suggesting that the oxidative species generated by **3** may be more efficiently cleared in cells compared to those generated by other complexes. For complex **1** the opposite effect was observed and despite its lower ability to induce ROS formation, the DSB were the highest among the group.

### 3.13 Induction of apoptosis in response to Cu(II) complexes

To determine the mode of cell death, A-375 cells were incubated with the Cu complexes and cisplatin for 48 h and, induction of apoptosis was evaluated using both Annexin-V staining and activation of caspase 3/7 in response to drug treatment. While Annexin-V staining relies on the loss of plasma membrane asymmetry, caspases 3 and 7 are critical proteases, which are responsible for the cleavage of many cellular proteins. Based on the selected assays that rely on different mechanisms, cells exhibited a dramatic increase in both the early and late apoptosis population, and once again **8** appeared as the most effective drug among the complexes tested herein (Figure 7). Complex **3** also performed well. All complexes appeared to be superior to cisplatin in terms of induction of Annexin-V and caspase 3/7 staining except for **5**, which triggered apoptosis only mildly (Figures 7C, D) despite the significant increase in oxidative stress and DNA breaks (∼80%, Figures 7A,B). Similar results were obtained in A-549 cells (Supplementary Figure S28).

### 3.14 Antibacterial activity of the ligand precursors and their Cu(II) complexes

The Cu(II) complexes selected for cell studies (**1**, **3**, **5**, **8**) as well as **6** and **7** and their ligand precursors were further investigated to reveal their antibacterial activity on the Gram-negative *Escherichia coli* and *Klebsiella pneumoniae* strains and the Gram-positive *S. aureus* using the sensitive (ATCC 25928) and the methicillin-resistant (MRSA 272123) subspecies (Supplementary Table S8). Neither the ligand precursors nor the Cu(II) salt had activity on the tested bacteria (MIC >100 μM). While complexes **6** and **8** had no measurable effect, **1** and **3** and **5** exerted effects with 50 and 100 μM MIC values against the sensitive and the methicillin-resistant subspecies, respectively. Complex **7** was found to be the most active with 12.5 μM (ATCC 25928) and 50 μM (MRSA) MIC values. It should be noted that *Staphylococcus aureus* causes a wide variety of clinical diseases, and the resistant MRSA subspecies is responsible for several community and hospital-acquired infections (Bal et al., 2017).

## 4 Conclusion

Several new benzoylhydrazones (L^n^, n = 1–8) were synthesized as well as eight new Cu(II) complexes by reaction of the ligand precursors (L^n^) with Cu(II) acetate. All compounds were characterized and the crystal and molecular structure of both L^5^ and its Cu-complex were obtained by SCXRD. The trinuclear structure was established for [Cu(L^5^)]_3_, and a similar formulation is proposed for [Cu(L^n^)]_3_ (n = 2, 3, 6, 7 and 8) in the solid phase. For **1** and **4** the presence of acetate in the solid-state structural formula of the complexes, precluded the formation of the oligomer.

All compounds present low solubility in water, but they are moderately soluble in DMSO, thus most studies in solution were carried out in media also containing DMSO. Solubility, lipophilicity and proton dissociation constants were determined for all ligand precursors, and for the copper(II) complexes formed by L^1^, L^5^ and L^6^ in 30% (v/v) DMSO/H_2_O. L^1^-L^8^ are present in solution in their neutral H_2_L form, and species [Cu(LH)]^+^, [Cu(L)] and [Cu(LH_−1_)]^−^ form in the Cu(II) − ligand systems (L = L^1^, L^5^ and L^6^), and also [Cu(LH_−2_)]^2−^ in the case of L^6^. Their formation constants were determined, and their binding modes were established by analysis of the spectrophotometric and EPR data, as well as by DFT methodologies. [Cu(L)] predominates at physiological pH and the relative amount of trinuclear species is not expected to be relevant in the low µM range of concentrations.

The direct redox reactions between these copper complexes and the physiological reducing agents GSH and AA were also evaluated; it was found that the reactions producing Cu(I) species proceed faster with GSH than with AA. Our data also suggests the release of the free ligand during the redox reaction, and that probably the generated Cu(I) complex also dissociates; Cu(I) complexes probably form with GSH (or AA), which are present in a high excess when compared to the 8HQ-containing ligand.

Based on the stability constants, for the systems with L^1^, L^5^ and L^6^ pCu values (these reflect the binding strength of the ligands) were computed at 1 μM total metal ion, and two different ligand concentrations at pH 7.4. The pCu values are in the range 7.3–9.0 (for *c*_L_ = 1 μM) and 9.4 – 12.7 (for *c*_L_ = 10 μM). These Cu(II) complexes may be considered as high stability species and the concentration distribution curves show that [Cu(L)] species predominate at physiological pH.

All complexes appeared to be potent on cancer cells, albeit with varying efficiencies in cell lines of different origins. Globally this group of complexes is more active against the melanoma cells (A-375), than in lung (A-549) cancer cells, but in both cases the IC_50_ values are in the low µM range. Moreover, our data show that whilst the Cu complexes under study can induce ROS and DSBs in both melanoma and lung adenocarcinoma cells, their ability to induce an apoptotic form of cell death varies, potentially as a result of different clearance mechanisms or efficiencies within the cells. In terms of induction of apoptosis, **3** was the second most effective complex tested in our analyses, despite its lower efficiency in triggering DSBs. This was consistent with our previous report since the oxidovanadium(IV) complex of L^3^ was the most effective among L^1^-L^6^ complexes. Considering the IC_50_ values, the ability to induce ROS formation, double-strand breaks and cell-death by apoptosis, within the set of compounds tested, **8** seems to be the most promising complex.

Having developed ligands with different substituents we tried to establish a SAR analysis and correlate chemical properties (such as solubility and logD values) with the compounds’ biological activity (IC_50_ values and macromolecules’ binding parameters), but in general no correlations were found. For the complexes there seems to be some correlation between higher aqueous solubility and higher biological activity, but we do not have sufficient data to clearly state this. The forces driving the interactions and biological effects cannot be easily explained by the electronic effects of the different substituents in the ligands. The only effect clearly seen is that the presence of nitrogen atoms impacts positively the biological activity more than the presence of oxygen atoms and, concerning the presence of halogens, that the F-containing compounds are more active than those with Cl. Moreover, groups with H-donor ability seem to perform better, especially in the lung A-549 cancer cells.

Interestingly, complex **7** is the one that shows the lowest MIC values in Gram-positive bacteria *S. Aureus* subspecies ATCC 25928 (12.5 μM) and MRSA (50 μM), with complexes **1** (50 μM) and **3** and **5** (100 μM) also showing an effect in the same bacteria.

## Data Availability

The SCXRD structures presented in this study can be found in online repositories. The names of the repository/repositories and accession number(s) can be found below: https://www.ccdc.cam.ac.uk/structures/- CCDC numbers 2182647-2182648.
