# Supplementary material for: Promising anticancer agents based on 8-hydroxyquinoline hydrazone copper(II) complexes
Source: Front Chem. 2023 Mar 21;11:1106349. doi: 10.3389/fchem.2023.1106349 (PMC10072326; doi:10.3389/fchem.2023.1106349)
Supplement: Supplementary file 1 [file DataSheet1.docx]

Supplementary Material

Promising anticancer agents based on 8-hydroxyquinoline hydrazone copper(II) complexes

Nádia Ribeiro^1^, Ipek Bulut,^2^ Baris Sergi,^2^ Vivien Pósa ^3^, Gabriella Spengler^3,4^, Giuseppe Sciortino,^5^ Vânia André,^1^ Liliana P. Ferreira,^6,7^ Tarita Biver,^8^ Valeria Ugone,^9^ Eugenio Garribba,^10^ João Costa Pessoa^1^, Éva A. Enyedy, ^3,*^ Ceyda Acilan,^2,11,*^, Isabel Correia^1, *^

S1 – Experimental part……………………………………………………….…………………………………..…2

S2 – Compounds’ spectroscopic characterization …………………………………….………………10

S3 – Solution behaviour ………………………………………………………………………………….……….16

S4 – Biological interactions ………………………………………………………………………..…..……....25

S5 – Cell studies …………………………………………………………..………………………….……………...32

References……………………………………………………………………………………………………………….35

## S1 – Experimental part

## S1.1 Single crystal X-ray diffraction (SCXRD)

Single crystals suitable for X-ray diffraction studies were obtained from solutions of L^5^ (in acetone) and complex **5** (in methanol). Crystals were mounted with Fomblin© in a cryoloop. Data were collected on a BRUKER D8 QUEST diffractometer with graphite-monochromated radiation (Mo Kα, λ=0.7107 Å) at 150 K. The X-ray generator was operated at 50 kV and 30 mA and the X-ray data collection was monitored by the APEX3 program (1). Data were corrected for Lorentzian polarization and absorption effects using the SAINT (2) and SADABS (3) programs. SHELXT 2014/4 (4) was used for the structure solution and SHELXL 2014/7 (5) was used for full-matrix least-squares refinement on *F*^2^. These programs are included in the WINGX-Version 2014.1 (6) program package. Non-hydrogen atoms were refined anisotropically. A full-matrix least-squares refinement was used for the non-hydrogen atoms with anisotropic thermal parameters. All the hydrogen atoms bonded were inserted in idealized positions and allowed to refine in the parent atom, except for the hydrogen atoms of the water molecules in L^5^ that were located from the electron density map. SQUEEZE (7) was used in complex **5** due to the impossibility of modelling disordered solvents. Crystal data and details of data collection for L^5^ and complex **5** are reported in **Table S1** (CCDC numbers 2182647-2182648). Illustrations of the molecular structures were made with Mercury (8).

**Table S1.** Crystal data and structure refinement details for L^5^ and complex **5**.

|  | L^5^ | Complex **5** |
| --- | --- | --- |
| Formula | C_36_H_36_N_6_O_9_ | C_54_H_39_Cu_3_N_9_O_9_ |
| M_w_ | 696.71 | 1170.00 |
| Crystal form, colour | Plate, colourless | Needle, dark red |
| Crystal size (mm) | 0.16 × 0.1 × 0.03 | 0.18 × 0.04 × 0.04 |
| Crystal system | Triclinic | Trigonal |
| Space group | *P*-1 | P-3 |
| *a*, Å | 7.0011(18) | 17.0982(10) |
| *b*, Å | 13.850(3) | 17.0982(10) |
| *c*, Å | 18.395(4) | 13.1831(8) |
| *α*, deg | 108.019(9) | 90 |
| *β*, deg | 97.254(10) | 90 |
| *γ*, deg | 95.567(10) | 120 |
| *Z* | 2 | 2 |
| *V*, Å^3^ | 1665.1(7) | 3337.7(4) |
| *T*, K | 150 | 150 |
| *D*_c_, g cm^−3^ | 1.390 | 1.143 |
| *μ*(Mo K*α*), mm^−1^ | 0.102 | 0.998 |
| θ range (°) | 2.244 – 25.499 | 1.375 – 25.498 |
| Refl. collected | 53461 | 49171 |
| Independent refl. | 6186 | 4122 |
| *R*_int_ | 0.1670 | 0.0570 |
| *R*_1_ *^a^*, *wR*_2_ *^b^* [*I* ≥ 2*σ*(*I*)] | 0.1509, 0.3668 | 0.1144, 0.2912 |
| GOF on *F*^2^ | 1.122 | 1.053 |

*^a^* *R*_1_ = Σ||*F*_o_| – |*F*_c_||/Σ|*F*_o_|. *^b^* *wR*_2_ = [Σ[*w*(*F*_o_^2^ – *F*_c_^2^)^2^]/Σ[*w*(*F*_o_^2^)^2^]]^1/2^

## S1.2 Stability under aqueous conditions

The complexes were monitored for their stability in an aqueous buffer (HEPES, 0.01 M, 0.1 M KCl, pH 7.4). Stock solutions of each complex were prepared in DMSO and diluted in the aqueous buffer to obtain final concentrations of 20 to 30 µM; this also ensures that the concentration of organic solvent in the working solutions is less than 1% (v/v). Samples were monitored by UV-Vis absorption spectroscopy for 6 h. A final measurement was taken after 24 h.

## S1.3 Lipophilicity and solubility of the ligands and copper complexes

Thermodynamic solubility (*S*) was measured for the new ligands, L^7^ and L^8^, since data for the rest of the series was previously reported (9), and for selected Cu(II) complexes: 1, 5 and 6. Solubility was measured for the saturated solutions in water at pH 7.40 in 20 mM HEPES at 25.0 ± 0.1 ^o^C. The concentration was determined by UV-Vis spectrophotometry using the stock solutions of the compounds with known concentrations dissolved in 100% DMSO, 50% and 25% (v/v) DMSO/buffered aqueous solutions for the calibration.

Distribution coefficient (*D*_7.40_) values of selected Cu(II) complexes (**1**, **5**, **6**) were determined by the shake-flask method in *n*-octanol/buffered aqueous solution (20 mM HEPES, pH 7.40, 25.0 ± 0.2 °C). The ionic strength was 0.10 M (KCl) in the two parallel measurements. The compounds were dissolved at 400 µM concentrations in *n*-octanol pre-saturated with buffer. The stock solution in *n*-octanol:buffer (1:10) was mixed with 360° vertical rotation for 3 h. After mixing the samples, they were centrifuged at 5000 rpm for 5 min. The two phases were separated and their UV-Vis spectra were recorded, after the dilution of the octanolic phase. The *D*_7.40_ values were calculated as follows:

$D_{7.40}= \frac{\mathrm{Abs}_{oct. phase after separation} /\mathrm{Abs}_{oct. stock solution}}{1- \mathrm{Abs}_{oct. phase after separation} /\mathrm{Abs}_{oct. stock solution}}\cdot\frac{V_{aq. phase}}{V_{oct. p hase}}$ (S1)

## S1.4 UV-Vis spectrophotometry

An Agilent Cary 8454 diode array spectrophotometer was used to record the UV-Vis spectra in the range of 200–800 nm. Titrations were carried out with a Metrohm 665 Dosimat burette and an Orion 710A pH-meter equipped with a Metrohm combined electrode. A ionic strength of 0.10 M (KCl) was used to keep the activity coefficients constant. The titrations were performed with 0.10 M carbonate-free KOH solution in a 30% (v/v) DMSO/H_2_O solvent mixture. The electrode system was calibrated to the pH = ‒log[H^+^] scale by means of blank titrations (strong acid *vs.* strong base: HCl *vs*. KOH) according to the method suggested by Irving *et al*. (10). The average water ionization constant (p*K*_w_) is 14.53 ± 0.05 in the 30% (v/v) DMSO/H_2_O solvent mixture. The p*K*_a_ values of the ligand precursors and the log*β* values for the Cu(II) complexes were determined in 30% (v/v) DMSO/H_2_O in the same way as it was done in our previous publications (9, 11).

Samples were deoxygenated by bubbling argon through the system for 10 min, and the argon flow was maintained during the titrations. Spectrophotometric titrations were carried out on samples containing the ligands at 50 μM concentration, in the pH range from 2.0 to 11.5 in the absence or the presence of 1, 0.5 or 0.25 equiv. Cu(II) ions, using 10 cm^3^ sample volumes. Proton dissociation constants (p*K*_a_) of the ligand precursors as well as stoichiometry and stability constants of the Cu(II) complexes (*β*) were calculated by the computer program PSEQUAD (12). The usual definition of overall (*β)* and stepwise (*K*) formation constants is used:

$\beta_{pqr}: p\mathrm{Cu}^{\mathrm{II}}+qL+rH^{+} \leftrightarrow[{{(Cu}^{\mathrm{II}})}_{p}\left( L \right)_{q}\left( H \right)_{r}]$ (S2)

$\beta_{pqr}= \frac{[{{(Cu}^{II})}_{p}\left( L \right)_{q}\left( H \right)_{r}]}{{[\left( {Cu}^{\mathrm{II}} \right)]}^{p}{[L]}^{q}{[H]}^{r}}$ (S3)

$\beta_{n}=K_{1}\times K_{2}\times\ldots K_{n}$ (S4)

## S1.5 Spectrophotometric kinetic measurements

For the redox reactions a special, tightly closed tandem cuvette (Hellma Tandem Cell, 238-QS) was used. The reduction was recorded at physiological pH (pH=7.4) in 50 mM HEPES buffer (with 0.1 M KCl) and the Cu(II) complexes: GSH or AA ratio was 1:100 (*c*_complex_ = 40 μM, *c*_GSH_ or *c*_AA_ = 4 mM). The stock solutions of the complexes and of GSH and AA were freshly prepared before every measurement. Argon was bubbled through the solutions for 10 min before measurements and the samples were kept under argon during spectrophotometric measurements. The reduction was usually followed for 2 h. In one half of the cuvette the complex was dissolved in HEPES buffer, and the other half contained GSH or AA in HEPES buffer. The first spectrum was recorded before mixing. This was followed by the mixing of the two parts of the cuvette, and changes were followed till no further absorbance changes were observed.

During the calculations, the absorbance (*A*) ‒ time (*t*) curves were fitted and analysed at 400 nm:

A = (A_0_‒A_final_) × e^(‒^*^a^*^×^*^t^*^)^ + A_final_ (S5)

where A_0_, A_final_ and *a* parameters were refined and accepted at the minimal value of the weighted sum of squared residuals (difference between the measured and calculated absorbance values) at the given wavelength. Then observed rate constants (*k*_obs_) of the redox reaction were obtained from the data points of the simulated absorbance-time curves as the slope of the ln(A/A^0^) *vs.* time plots.

## S1.6 Cyclic voltammetry

Cyclic voltammograms of the Cu(II) complexes were measured in 90% (v/v) DMSO/water (HEPES, 0.01 M, pH 7.4) solution containing 1 mM of ligand precursor and CuCl_2_. The ionic strength was 0.10 M, adjusted by TBAN. Samples were deoxygenated by bubbling argon through the system for 10 min before recording the cyclic voltammograms. Glassy carbon, platinum and Ag/AgCl (3 M KCl) electrodes were used as the working, auxiliary and reference electrodes, respectively. Electrochemical potentials were converted into the normal hydrogen electrode (NHE) scale by adding 0.222 V. The electrochemical system was calibrated with an aqueous solution of K_3_[Fe(CN)_6_]. Redox potentials were obtained at different scan rates (5-25 mV/s) in the range of −0.3 to +0.7 V.

**S1.7 EPR measurements**

EPR spectra of the solid complexes [Cu(HL^1^)(AcO)] (**1**), [Cu(HL^4^)(AcO)] (**4**), [Cu(L^5^)]_3_ (**5**) (polycrystalline solid and crystals), [Cu(L^6^)]_3_ (**6**) and [Cu(L^7^)]_3_ (**7**) were recorded at 120 K both on the powder samples and after dissolving the solids in DMSO.

The spectra in 80% (v/v) DMSO/H_2_O were recorded weighting ^63^CuSO_4_·5H_2_O and ligands L^4^ or L^6^ to obtain a copper to ligand molar ratio of 1:1 and a Cu(II) concentration of 1.0 mM. The pH values in the solvent mixtures were measured after calibrating the electrode in 80% (v/v) DMSO/H_2_O buffer.

**S1.8 DFT calculations**

The geometry of the Cu(II) complexes formed by L^6^ was optimized with Gaussian 16 software, rev B.01 (13) at the DFT theory level using the hybrid B3LYP functional combined with Grimme’s D3 correction (14) for dispersion and the split-valence plus polarization function 6-31g(d,p) basis set for the main group elements, SDD plus *f*-functions (BS1) (15) and pseudopotential for copper. The effect of solvation was taken into account using the SMD continuum model of Marenich *et al.* (16). Frequency calculations were carried out for all the optimized geometries in order to characterize the energy minima (17). A correction of 1.9 kcal·mol^−1^ was applied to all Gibbs values to change the standard state from the gas phase (1 atm) to solution (1 M) at 298.15 K (18).

## S1.9 Binding to bovine serum albumin (BSA)

To evaluate the interaction of the compounds, both organic and Cu(II) complexes, with bovine serum albumin (BSA) fluorescence spectra were measured. In a typical fluorescence experiment, to a solution of 1.5 μM of BSA in the same buffer, successive aliquots of a stock solution of each compound in DMSO were added directly to the cuvette (optical path 1.0 cm). The fluorescence emission was recorded between 310 and 600 nm with excitation at 295 nm. The final DMSO % was kept below 2% (v/v). The Stern-Volmer plots were built using the emission at 340 nm and blank samples (in the absence of BSA) with the same concentration of the complexes were recorded and subsequently subtracted from the corresponding emission spectra containing the fluorophore. Moreover, the fluorescence emission intensity was corrected for reabsorption and inner filter effects using UV-Vis absorption data recorded for each sample, since all complexes absorb in the measured wavelength range (19, 20).

## S1.10 Interaction with *calf thymus* DNA

The thermal denaturation of calf thymus DNA (*ct*DNA) (in the absence and the presence of selected compounds) was monitored by UV-Vis absorption using a Shimadzu UV-2450 spectrophotometer connected with a Peltier thermostat. In a typical experiment, the working solution was heated from 25.0 to 95.0 °C. After the measurement of the initial spectrum at 25.0 °C, the temperature was raised by 5.0 °C in 1 min. After keeping the temperature constant for 6.0 min to allow the system to reach equilibrium, an absorption spectrum was recorded. This procedure was repeated till reaching the final temperature. Thermal denaturation plots were obtained as the variation of absorbance at 260 nm with temperature; the melting temperature (T_m_) was determined as the inflection point of the sigmoidal fit of the experimental points.

The fluorescence emission experiments were carried out in a Perkin Elmer LS 55 spectrofluorimeter. A pulsed Xenon lamp (50 Hz) provided the excitation light. A thermostated water bath recycler linked to the cell holder provided temperature control within ± 0.1 °C. The measurements were done in a 1000 µL quartz cell with an optical path length of 1.0 cm with the complexes solution added directly to the DNA-EB (ethidium bromide) solution in the cell. A glass syringe connected to a Mitutoyo micrometric screw added accurate volumes each time (one complete turn of the screw is 8.2 µL, 1/50 of a turn is the minimum possible addition).

**S1.11 Agarose gel electrophoresis for complex/DNA interactions**

The nature of the interaction between the complexes and pure plasmid DNA was evaluated by agarose gel electrophoresis as described previously with minor modifications (21). Briefly, free ligands, copper complexes, and cisplatin (200 and 400 μM; in 20 μL total volume) were incubated overnight at room temperature with plasmid DNA (200 ng, H2B-GFP, Addgene #11680). NaN_3_ (final: 37.5 mM in ddH_2_O) was used as a ROS scavenger. Samples were run on a 1% agarose gel (100 V, 60 min). The experiments were carried out with two biological replicates, and ImageJ software was used to quantify and analyse the intensity of the bands.

**S1.12 Cell Culture conditions**

A-375 cells (ATCC, CRL-3222) were grown in Dulbecco’s Modified Eagle Medium with high glucose (Gibco #11965118) and A-549 (ATCC, CCL-185) cells were grown in Dulbecco’s Modified Eagle Medium F-12 (Gibco #11320033), supplemented with 10% FBS (Gibco, #10500064) and Penicillin-Streptomycin (10,000 U/mL, Gibco, #15140122). Cells were incubated at 37 °C, 5% CO_2_ incubator and subcultured every 3 days.

**S1.13 Sulforhodamine B (SRB) assay**

All the tested complexes were dissolved in DMSO (10 mM stock solution), and aliquots were stored at -20 °C, thawed on the experiment day and used only once. For SRB cell viability assay, A-375 and A-549 cells were seeded on 96-well plates (4×10^3^ cells/well) and treated with serial dilutions of indicated compounds (with a concentration range of 0-50 μM, 72 h). Trichloroacetic acid (TCA) was added to wells for fixation at a final concentration of 10%, for 1 h at 4 °C. Each well was washed five times with ddH_2_O, dried, and incubated with 0.4% (w/v) SRB dye (50 µL/well) for 30 min at room temperature. The wells were washed with 1% acetic acid (v/v) five times, and 150 µL of 10 mM Tris solution was added to dissolve the stain. Colorimetric measurements were taken at λ = 564 nm. Viability percentage was calculated as follows: % Cell Viability = [100 × (sample Abs.-blank) / (non-treated control Abs.-blank)].

**S1.14 Flow cytometry analyses**

5 × 10^4^ number of A-375 and A-549 cells were seeded on 12-well plates, and the next day cells were treated with either copper complexes (calculated IC_70_ values) or cisplatin (15 μM). Following 48 h of treatment, cells were trypsinized, centrifuged and resuspended in PBS solution supplemented with 1% fetal bovine serum (FBS) (10^6^ cells/mL).

**S1.15 Annexin V staining**

Annexin V & Dead Cell Kit (Luminex, MCH100105) protocols were followed for Annexin V activation. Briefly, 1 × 10^5^ cells were stained with Annexin V and 7-AAD solution in a 1:1 ratio and incubated at room temperature for 20 min. The apoptotic cell population was evaluated with Muse Cell Analyzer (Merck Millipore).

**S1.16 Caspase 3/7 staining**

Caspase-3/7 Kit (Luminex, MCH100108) protocols were followed for Caspase 3/7 staining. 2 × 10^5^ cells were stained with 5 μL of Caspase 3/7 antibody (1:8 dilution in PBS) and incubated at room temperature for 30 min. Then, the cells were stained with 100 μL of 7-AAD antibody mixture (1:75 dilution with 1 × Assay Buffer) for 5 min. Caspase 3/7 and 7-AAD positivity were assessed with Muse Cell Analyzer (Merck Millipore).

**S1.17 ɣH2AX staining**

The Muse ɣH2A.X Activation Dual Detection Kit (Luminex, MCH200101) protocols were followed with minor changes. 1 × 10^5^ cells were mixed with ice-cold 1X Fixation Buffer at the same volume for 5 min. Cells were centrifuged (600 g, 5 min), and the pellet was suspended with 100 μL of ice-cold 1X Permeabilization Buffer (10 min, on ice), and centrifuged again. The cell pellet was resuspended with 50 μL of antibody mixture of (20X) Anti-phospho-Histone H2A.X (Ser139), Alexa Fluor 555 and (20X) Anti-H2A.X, PECy5 antibodies. Following 30 min of antibody staining, cells were centrifuged and resuspended with 1X Assay Buffer. DNA breaks were analysed with Muse Cell Analyzer (Merck Millipore).

**S1.18 ROS activation**

Oxidative stress levels were assessed by Muse Oxidative Stress Kit (Luminex, MCH100111) using the same protocol as previously described (21).

## S1.19 Bacterial cell culture and MIC determination

Wild-type *Escherichia coli* K-12 AG100 strain [argE3 thi-1 rpsL xyl mtl Δ(gal-uvrB) supE44] expressing the AcrAB TolC efflux pump at its basal level and *Klebsiella pneumoniae* ATCC 49619 Gram-negative strains were studied in the experiments. Sensitive (ATCC 25928) and the methicillin-resistant (MRSA 272123) subspecies of *Staphylococcus aureus* were used as Gram-positive strains in the assays.

Minimum inhibitory concentration (MIC) values of selected compounds were determined in 96-well plates based on the Clinical and Laboratory Standard Institute guidelines (CLSI guidelines) [50]. The stock solutions of the compounds were dissolved in DMSO using 1.3 μL of Mueller Hinton Broth, and then two-fold serial dilutions were performed. The starting concentration of the compounds was 100 μM. Then 10^‒4^ dilution of an overnight bacterial culture in 100 μL of medium was added to each well, except for the medium control wells. The plates were further incubated at 37 °C for 18 h; at the end of the incubation period, the MIC values of tested compounds were determined by visual inspection.

S2 – Compounds’ spectroscopic characterization

Table S2 – ^1^H and ^13^C NMR chemical shifts (in DMSO_*d_6_* at 300 MHz) for the synthesized ligand precursors.

|  | \|  \| position 20 \| position 23 \| \| --- \| --- \| --- \| \| **L^1^** \| C \| H \| \| **L^2^** \| C \| Cl \| \| **L^3^** \| C \| F \| \| **L^4^** \| C \| CH_3_ \| \| **L^5^** \| C \| OCH_3_ \| \| **L^6^** \| C \| OH \| \| **L^7^** \| C \| NH_2_ \| \| **L^8^** \| N \| na \| |
| --- | --- | --- | --- | --- | --- | --- | --- | --- | --- | --- | --- | --- | --- | --- | --- | --- | --- | --- | --- | --- | --- | --- | --- | --- | --- | --- | --- | --- |

|  | **L^1^** | | **L^2^** | | **L^3^*** | | **L^4^** | | **L^5^** | | | **L^6^** | | **L^7^** | | **L^8^** | |
| --- | --- | --- | --- | --- | --- | --- | --- | --- | --- | --- | --- | --- | --- | --- | --- | --- | --- |
|  | H | C | H | C | H | C | H | C | H | C | H | | C | H | C | H | C |
| 1 | 7.47 | 128 | 7.47 | 128.2 | 7.47 | 128.4 | 7.45 | 128.3 | 7.46 | 128.2 | 7.55 | | 128.7 | 7.53 | 128.7 | 7.56 | 128.7 |
| 2 | 7.13 | 111.9 | 7.14 | 112.1 | 7.13 | 112.2 | 7.14 | 112.1 | 7.13 | 112.3 | 7.29 | | 112.8 | 7.28 | 112.7 | 7.29 | 112.8 |
| 3 | q | 152.9 | q | 154.2 | q | 153.5 | q | 153.5 | q | 153.3 | q | | 153 | q | 153.3 | q | 153 |
| 4 | q | 138.1 | q | 137.8 | q | 138.1 | q | 137.8 | q | 138.5 | q | | 136.9 | q | 137.4 | q | 136.5 |
| 5 | q | 128.4 | q | 128.8 | q | 128.9 | q | 128.5 | q | 128.8 | q | | 128.3 | q | 128.8 | q | 128.8 |
| 6 | 7.43 | 117.7 | 7.43 | 118.6 | 7.43 | 118.0 | 7.42 | 117.7 | 7.41 | 117.7 | 7.44 | | 118 | 7.47 | 118.1 | 7.5 | 118.1 |
| 7 | N | N | N | N | N | N | N | N | N | N | N | | N | N | N | N | N |
| 8 | q | 151.5 | q | 153.4 | q | 151.7 | q | 151.4 | q | 152.3 | q | | 150.7 | q | 150.8 | q | 150.3 |
| 9 | 8.12 | 117.4 | 8.1 | 117.7 | 8.10 | 117.9 | 8.11 | 117.7 | 8.1 | 117.9 | 7.84 | | 123 | 7.82 | 122.8 | 7.89 | 123.1 |
| 10 | 8.36 | 136.5 | 8.36 | 136.7 | 8.36 | 136.7 | 8.34 | 136.5 | 8.34 | 136.6 | 8.54 | | 137.9 | 8.52 | 137.8 | 8.57 | 138.4 |
| 11 | 9.83 | OH | 9.86 | OH | 9.85 | OH | 9.85 | OH | 9.83 | OH | 10.85 | | OH | 10.86 | OH | 10.86 | OH |
| 12 | 8.66 | 147.7 | 8.64 | 148.2 | 8.64 | 148.0 | 8.65 | 147.6 | 8.64 | 147.2 | 7.83 | | 137.2 | 7.76 | 136.5 | 7.96 | 140.2 |
| 13 | N | N | N | N | N | N | N | N | N | N | N | | N | N | N | N | N |
| 14 | 12.22 | NH | 12.3 | NH | 12.25 | NH | 12.17 | NH | 12.12 | NH | 15.67 | | NH | 15.5 | NH | 16.08 | NH |
| 15 | q | 163.5 | q | 161.8 | q | 162.5 | q | 163.1 | q | 163 | q | | 164 | q | 163.7 | q | 161.9 |
| 16 | q | 133.9 | q | 132.5 | q | 148.9 | q | 130.6 | q | 125.5 | q | | 122.7 | q | 118.7 | q | 141.6 |
| 17 | O | O | O | O | O | O | O | O | O | O | O | | O | O | O | O | O |
| 18 | 7.95 | 127.8 | 7.99 | 129.7 | 8.03 | 130.6 | 7.87 | 127.6 | 7.95 | 129.8 | 8.23 | | 130.3 | 8.07 | 129.9 | 8.19 | 121.7 |
| 19 | 7.57 | 128.2 | 7.66 | 128.6 | 7.43 | 115.9 | 7.37 | 129.1 | 7.1 | 113.8 | 6.96 | | 115.1 | 6.67 | 112.7 | 8.86 | 150.6 |
| 20 | 7.62 | 131.5 | q | 137.2 | q | 133.3 | q | 142.1 | q | 162.5 | q | | 161.2 | q | 153 | N | N |
| 21 | 7.57 | 128.2 | 7.66 | 128.6 | 7.43 | 115.9 | 7.37 | 129.1 | 7.1 | 113.8 | 6.96 | | 115.1 | 6.67 | 112.7 | 8.86 | 150.6 |
| 22 | 7.95 | 127.8 | 7.99 | 129.7 | 8.03 | 130.6 | 7.87 | 127.6 | 7.95 | 129.8 | 8.23 | | 130.3 | 8.07 | 129.9 | 8.19 | 121.7 |
| 23 | na | na | Cl | Cl | F | F | 2.4 | 20.9 | 3.85 | 55.51 | 10.26 | | OH | 5.93 | NH_2_ | na | na |

* Results obtained at 400 MHz spectrometer; q – quaternary carbon; na – not applied.

**Table S3** – Selected FTIR and UV-Vis bands for ligand precursors and Cu(II)-complexes

|  | FTIR (cm^−1^) | | | | | | UV-Vis [DMSO, λ (nm)/ (ε (M^−1^ cm^−1^))] | | | | |
| --- | --- | --- | --- | --- | --- | --- | --- | --- | --- | --- | --- |
|  | ν(OH) | ν(NH) | ν(CH) | ν(C=O) | ν(CN)_qui_ | ν(C=N)_im_ | λ_1_ (ε_1_) | λ_2_ (ε_2_) | λ_3_ (ε_3_) | λ_4_ (ε_4_) | λ_5_ (ε_5_) |
| L^1^ | 3359 | 3319 | 3051 | 1687 | 1678 | 1547 | 264 (2.1 x 10^4^) | 303 (5.1 x 10^4^) | 328 (3.1 x 10^4^) | 337 (2.9 x 10^4^) |  |
| L^2^ | 3411 | 3242 | 3077 | 1658 | 1651 | 1562 | 266 (1.8 x 10^4^) | 296 (4.1 x 10^4^) | 336 (2.6 x 10^4^) | 375 (5.0 x 10^3^) |  |
| L^3^ | 3400 | 3209 | 3040 | 1653 | 1645 | 1558 | 267 (1.5 x 10^4^) | 297 (3.7 x 10^4^) | 330 (2.2 x 10^4^) | 375 (4.0 x 10^3^) |  |
| L^4^ | 3386 | 3284 | 3044 | 1668 | 1634 | 1553 | 260 (2.0 x 10^4^) | 300 (4.7 x 10^4^) | 350 (3.0 x 10^4^) | 375 (5.0 x 10^3^) |  |
| L^5^ | 3452 | 3284 | 3047 | 1656 | 1645 | 1555 | 260 (1.7 x 10^4^) | 305 (4.5 x 10^4^) | 350 (4.5 x 10^4^) | 370 (5.0 x 10^3^) |  |
| L^6^ | 3359 | 3303 | 3047 | 1668 | 1657 | 1541 | 263 (1.7 x 10^4^) | 306 (3.0 x 10^4^) | 340 (1.9 x 10^4^) | 364 (9.5 x 10^3^) |  |
| L^7^ | 3441 | 3318 | 3046 | 1645 | 1625 | 1548 | 265 (2.2 x 10^4^) | 284 (2.8 x 10^4^) | 295 (2.9 x 10^4^) | 350 (2.4 x 10^4^) |  |
| L^8^ | 3451 | 3326 | 3046 | 1679 | 1661 | 1549 | 260 (2.0 x 10^4^) | 295 (2.4 x 10^4^) | 344 (1.3 x 10^4^) | 356 (1.4 x10^4^) | 400 (1.5 x 10^3^) |
| **1** | 3415 (br) | | 3054 | 1664 | 1586 | 1512 | 277 (2.2 x 10^4^) | 331 (2.3 x 10^4^) | 390 (1.2 x 10^4^) | 478 (2.4 x 10^3^) |  |
| **2** | 3428 (br) | | 3021 | 1662 | 1593 | 1506 | 282 (9.0 x 10^3^) | 332 (8.0 x 10^3^) | 405 (6.0 x 10^3^) | 428 (5.5 x 10^3^) | 556 (3.8 x 10^3^) |
| **3** | 3423 (br) | | 3020 | 1662 | 1601 | 1509 | 280 (1.3 x 10^4^) | 327 (1.3 x 10^4^) | 394 (7.0 x 10^3^) | 425 (4.5 x 10^3^) | 535 (1.8 x 10^3^) |
| **4** | 3420 (br) | | 3052 | 1662 | 1608 | 1518 | 278 (1.3 x 10^4^) | 327 (2.0 x 10^4^) | 402 (1.3 x 10^4^) | 426 (7.0 x 10^3^) | 540 (1.0 x 10^3^) |
| **5** | 3386 (br) | | 3046 | 1657 | 1603 | 1506 | 281 (1.5 x 10^4^) | 335 (1.5 x 10^4^) | 399 (9.0 x 10^3^) | 429 (6.0 x 10^3^) | 560 (9.0 x 10^2^) |
| **6** | 3414 (br) | | 3060 | 1648 | 1605 | 1524 | 281 (2.3 x 10^4^) | 332 (1.9 x 10^4^) | 407 (1.2 x 10^4^) | 478 (3.0 x 10^3^) | 549 (1.1 x 10^3^) |
| **7** | 3440 (br) | | 3036 | 1625 | 1602 | 1523 | 291 (2.9 x 10^4^) | 345 (1.6 x 10^4^) | 417 (1.3 x 10^4^) |  | 535 (1.4 x 10^3^) |
| **8** | 3435 (br) | | 3047 | 1628 | 1595 | 1521 | 276 (1.1 x 10^4^) | 340 (9.0 x 10^3^) | 387 (6.0 x 10^3^) | 495 (1.6 x 10^3^) |  |

| 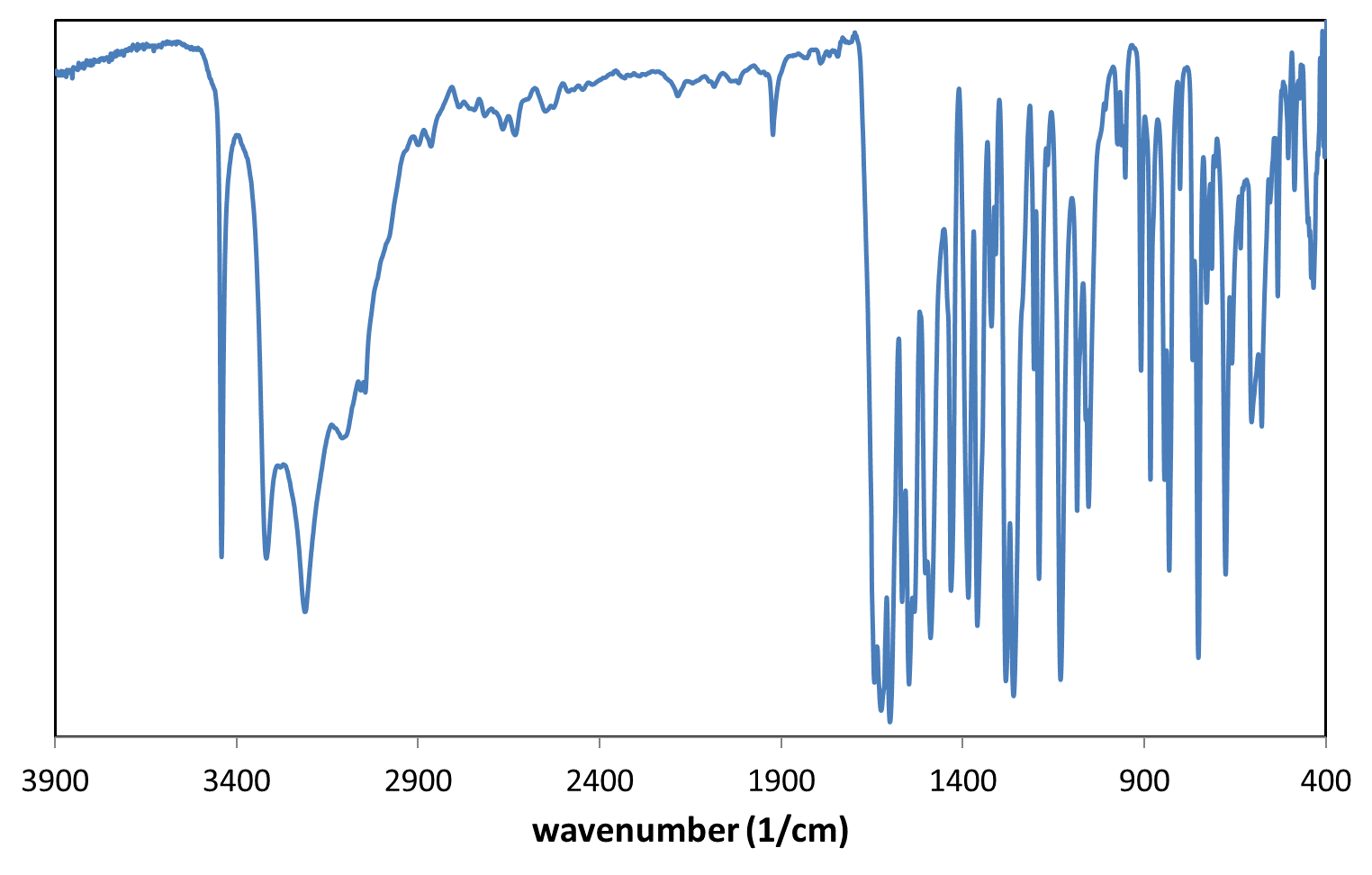  A | 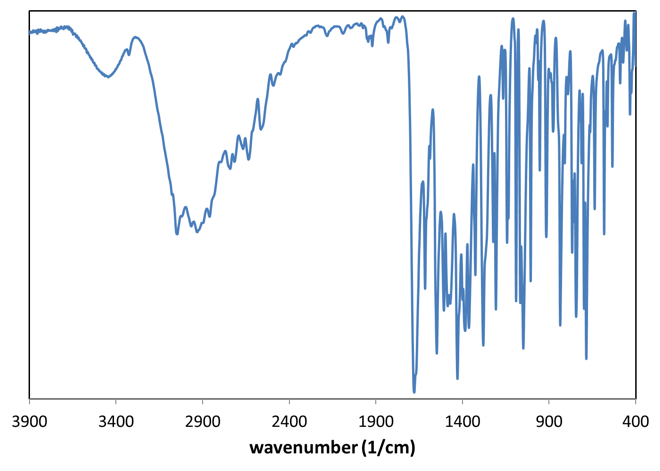  B |
| --- | --- |

**Figure S1** – FTIR spectrum of L^7^ (A) and L^8^ (B) as KBr pellets.


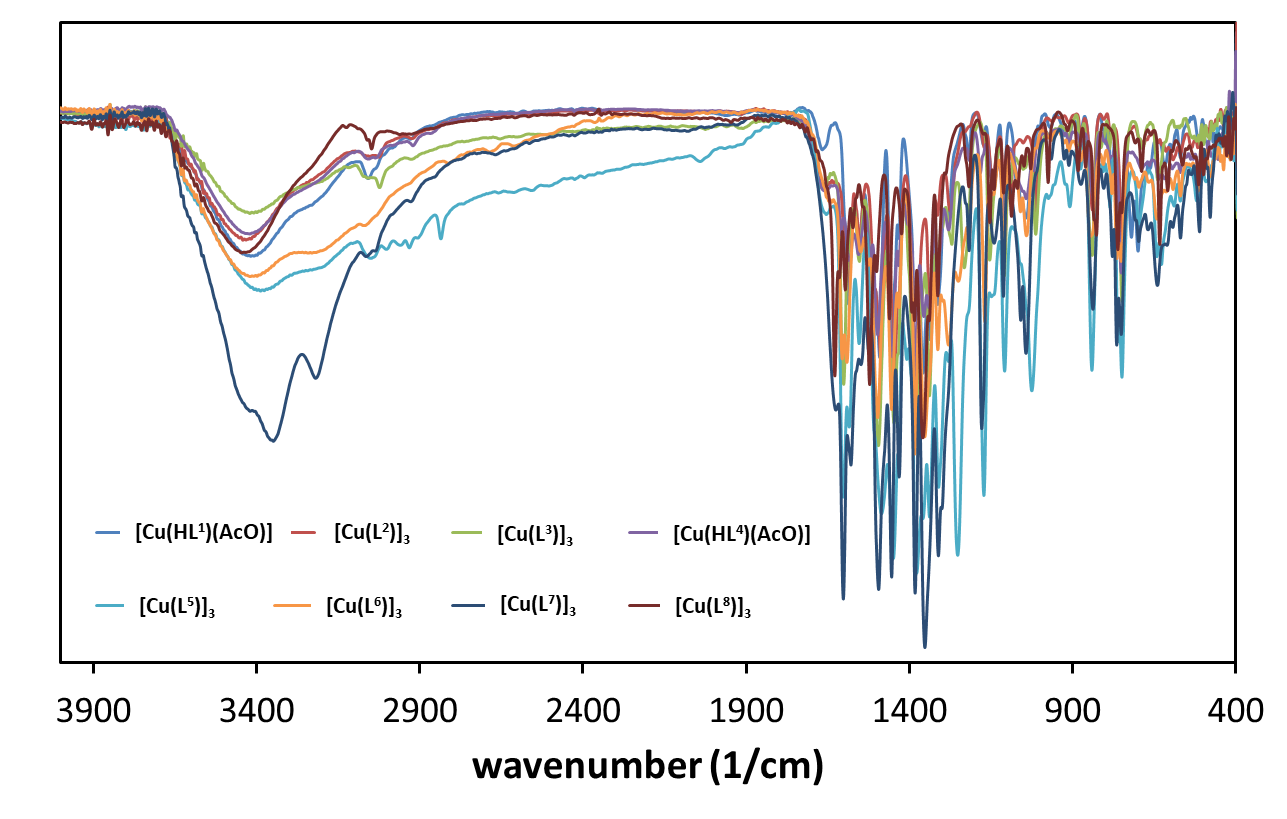


**Figure S2** – FTIR spectra of the copper complexes obtained as KBr pellets.

| 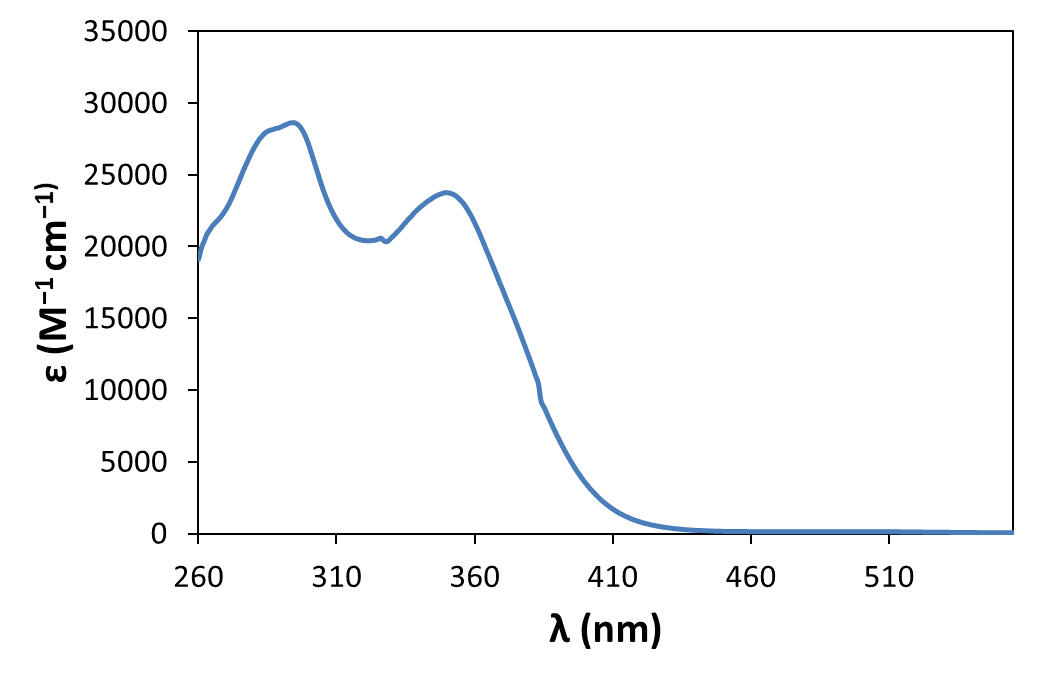 | 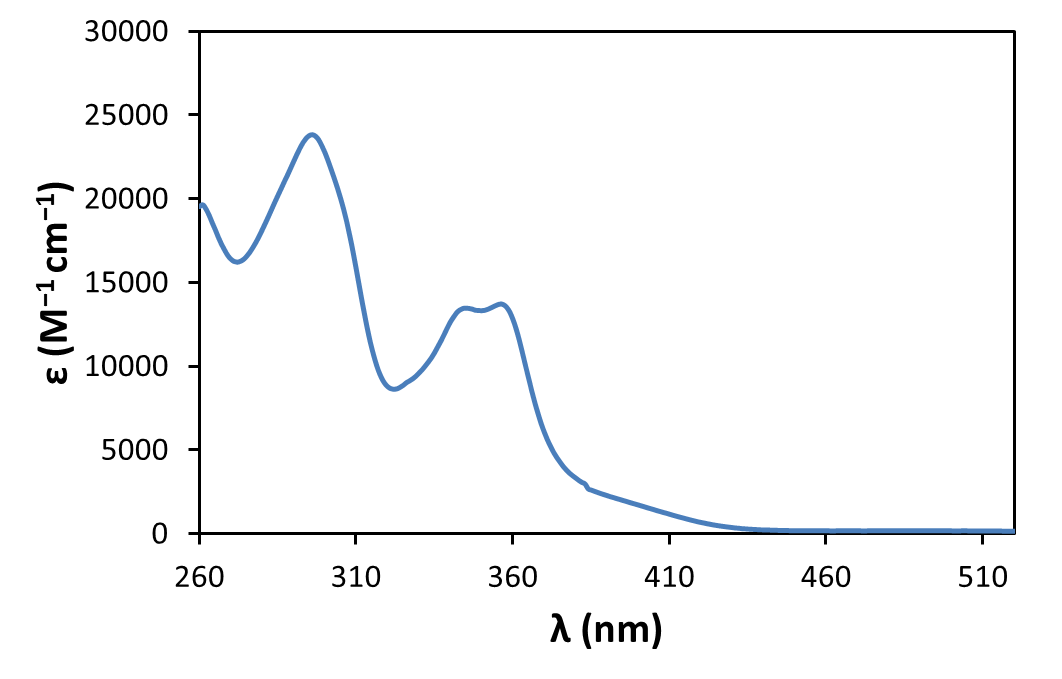 |
| --- | --- |

**Figure S3** – UV-Vis absorption spectra of L^7^ (left) and L^8^ (right) in DMSO.

| 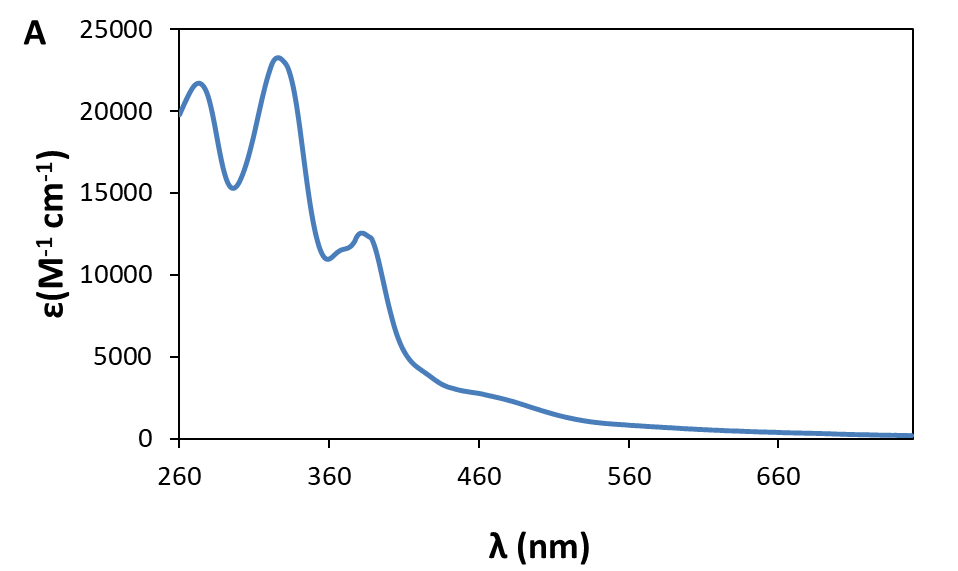 | 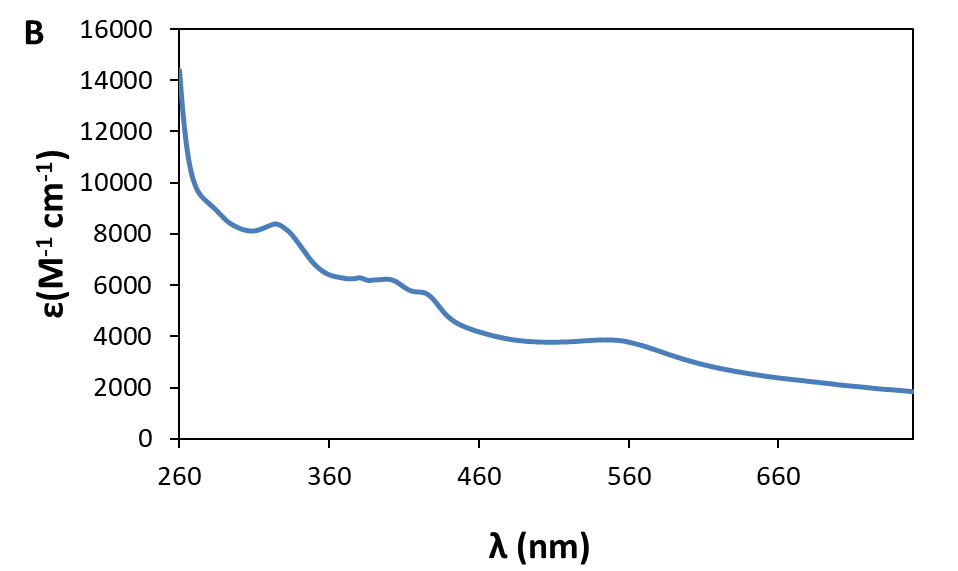 |
| --- | --- |
| 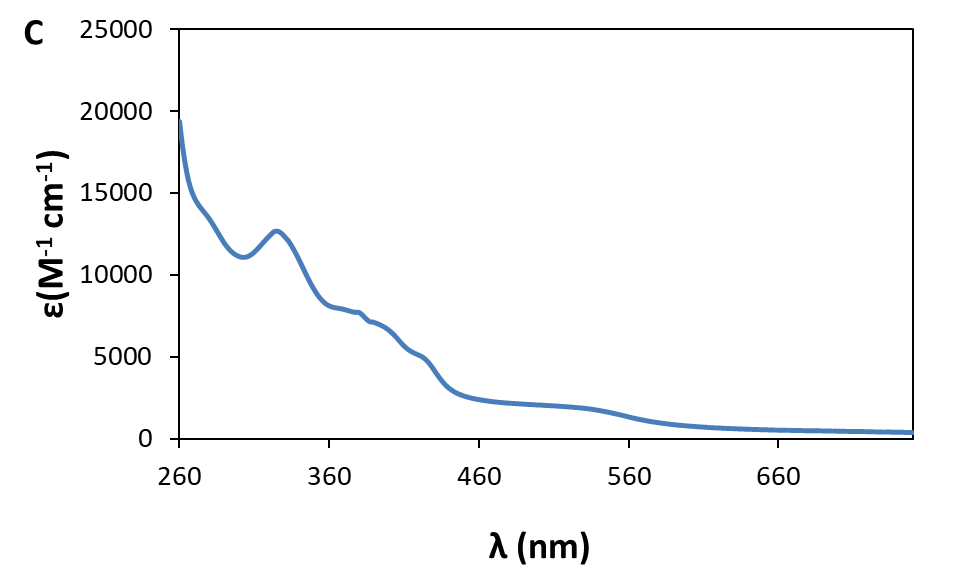 | 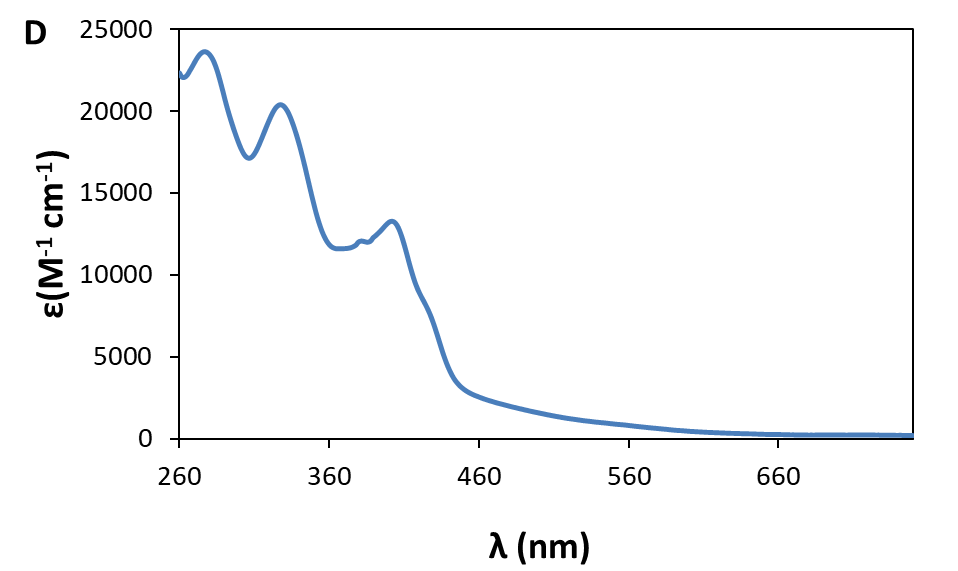 |
| 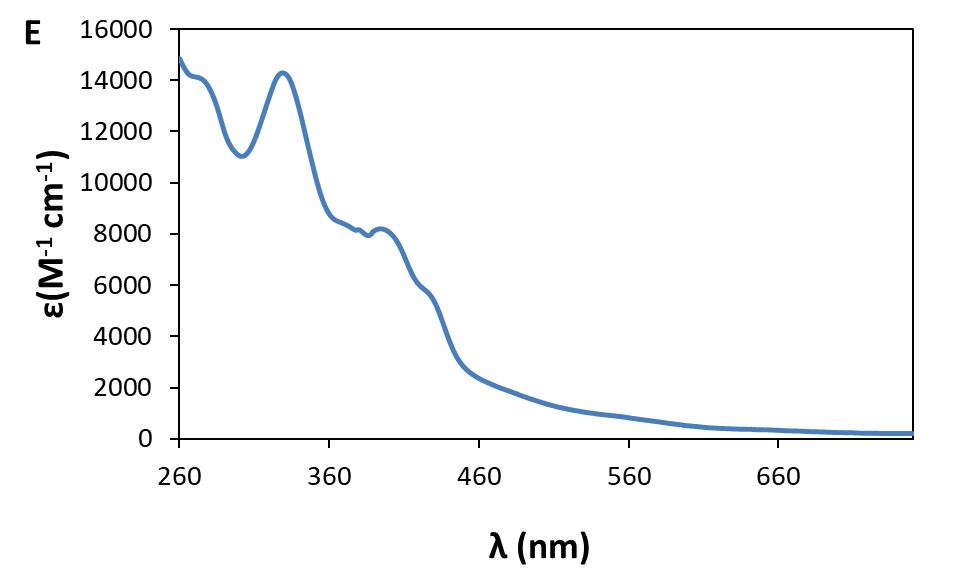 | 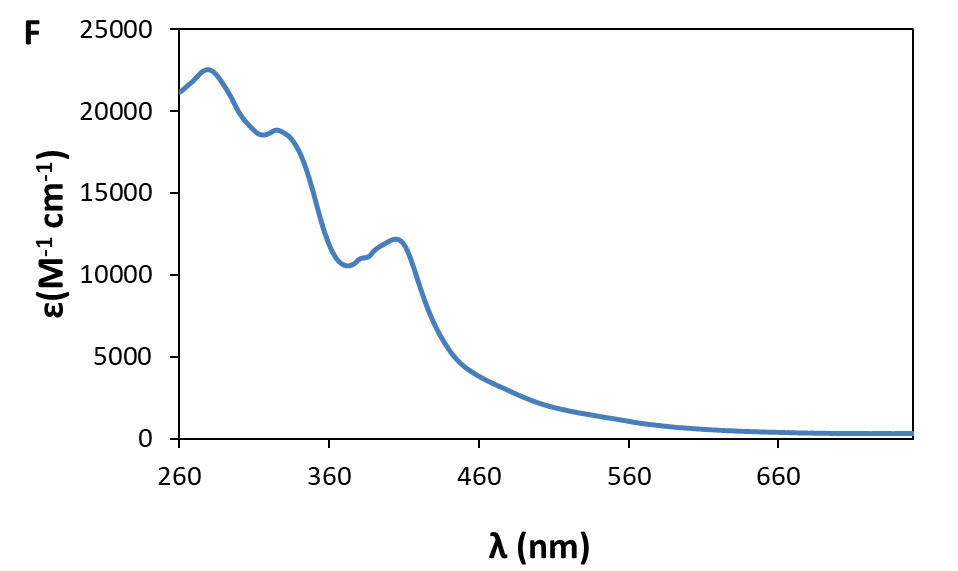 |
| 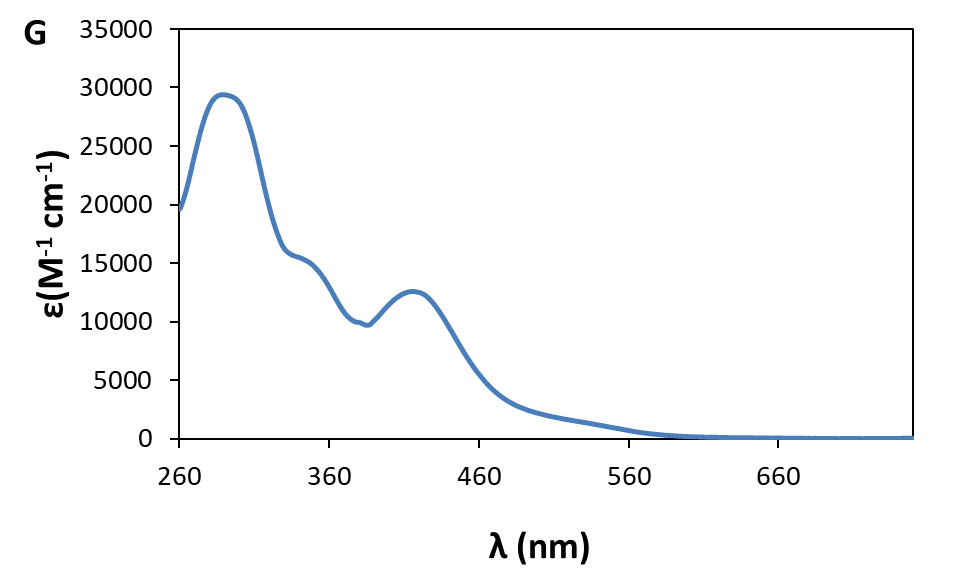 | 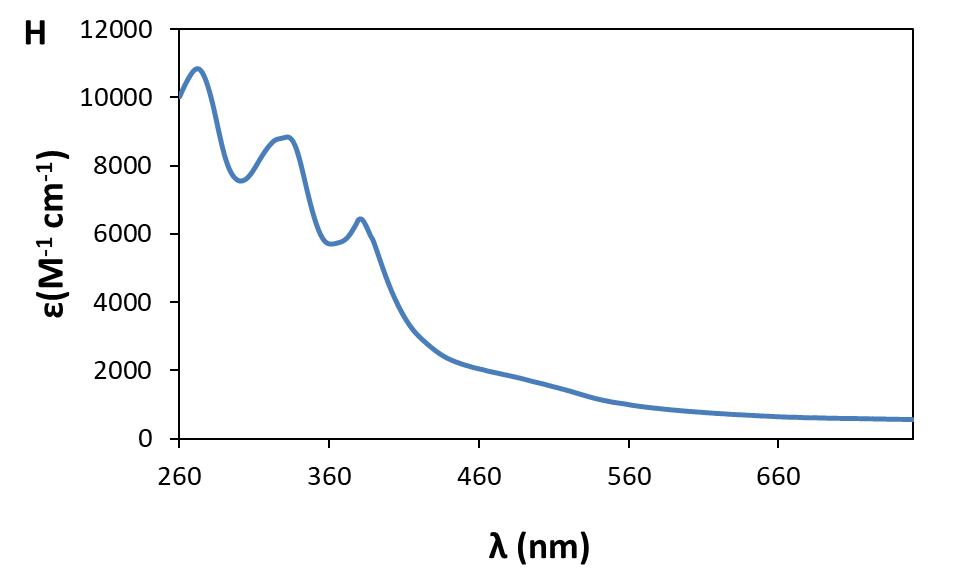 |

**Figure S4** – UV-Vis absorption of the complexes in DMSO. A – [Cu(HL^1^)(AcO)] (**1**), B – [Cu(L^2^)]_3_ (**2**), C – [Cu(L^3^)]_3_ (**3**), D – [Cu(HL^4^)(AcO)] (**4**), E – [Cu(L^5^)]_3_ (**5**), F – [Cu(L^6^)]_3_ (**6**), G – [Cu(L^7^)]_3_ (**7**), H – [Cu(L^8^)]_3_ (**8**).

**Table S4** – Hydrogen bond distances (Å) and angles (°) of L^5^.

| D-H^...^A | d(D-H) | d(H^...^A) | d(D^...^A) | (DĤA) | Symmetry operation |
| --- | --- | --- | --- | --- | --- |
| O3W-H1W^…^O17A | 0.86 | 1.96 | 2.804(8) | 170 | 1-x, -y, 1-z |
| O3W-H2W^…^O17A | 0.86 | 2.10 | 2.899(8) | 155 | 1+x, y, z |
| O1W-H4W^…^O2W | 0.74 | 2.09 | 2.780(9) | 154 | 1-x, 1-y, 1-z |
| O2W-H5W^…^O17 | 0.86 | 2.07 | 2.750(8) | 136 | 1-x, 1-y, 1-z |
| O2W-H6W^…^O17 | 0.86 | 2.24 | 2.880(8) | 132 | -1+x, y, z |
| O11-H11^…^O3W | 0.82 | 2.06 | 2.791(8) | 148 | 2-x, -y, 1-z |
| O11A-H11A^…^O1W | 0.82 | 2.08 | 2.750(7) | 139 |  |
| N14-H14^…^O3W | 0.86 | 2.15 | 2.931(9) | 150 |  |
| N14A-H14A^…^O1W | 0.86 | 2.14 | 2.931(9) | 153 | 1-x, 1-y, 1-z |

**Figure S5 -** Temperature dependence of the molar magnetic susceptibility (χ_m_, open symbols) and of the χ_m_T product (solid symbols), obtained with 0.1 T applied magnetic field. The solid lines represent the fit of the isotropic spin Hamiltonian to the results obtained with complex **5** in the crystalline form.

**S3 – Solution behaviour**


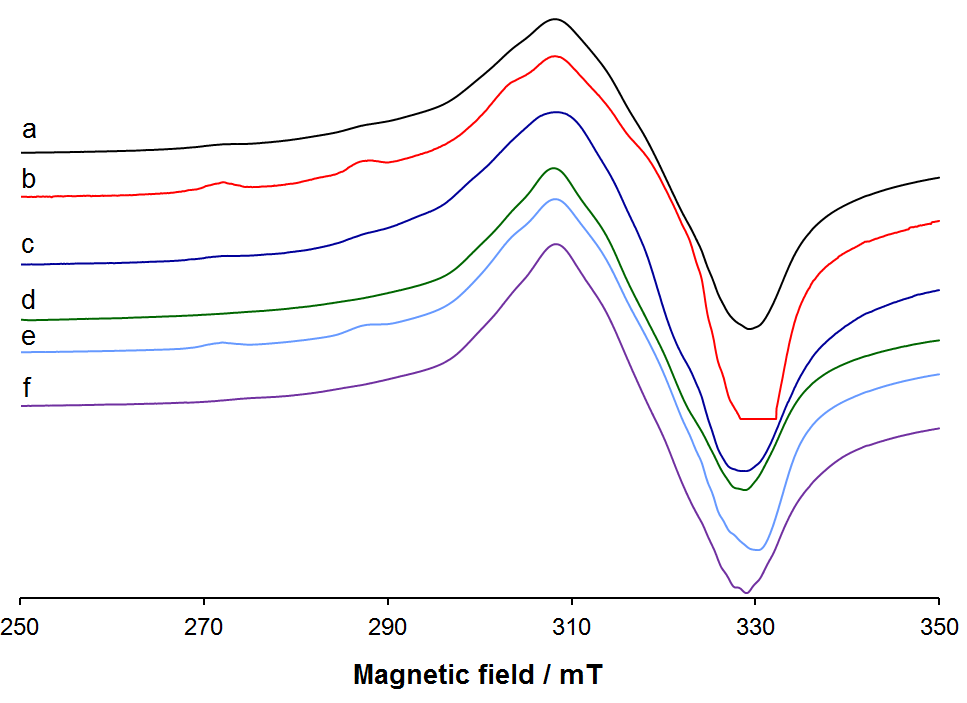


**Figure S6 -** X-band EPR spectra recorded at 120 K on samples of polycrystalline **1** (a), polycrystalline **4** (b), polycrystalline **5** (c), crystalline **5** (d), polycrystalline **6** (e) and polycrystalline **7** (f), all dissolved in DMSO.


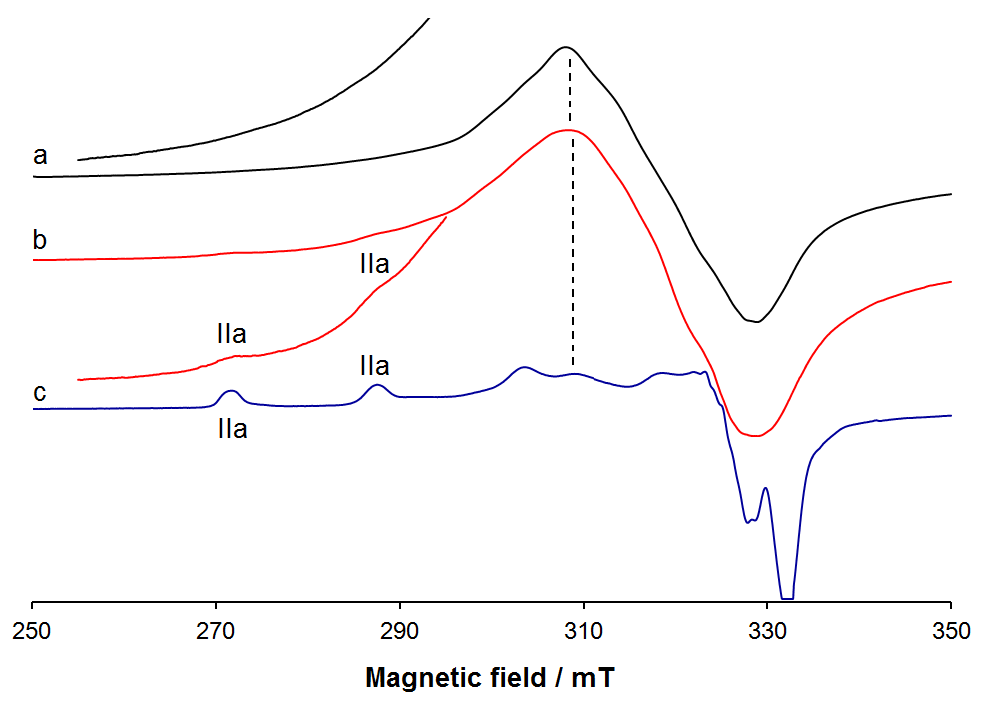


**Figure S7** – X-band EPR spectra recorded at 120 K on the crystalline complex **5** (a) and polycrystalline complex **5** (b) dissolved in DMSO. The region between 255.0 and 295.0 mT of the traces (a) and (b) is amplified by four and three times, respectively. In (c) the spectrum recorded in the system Cu(II)/H_2_L^4^ 1/1 at pH 6.50 in the mixture 80% (v/v) DMSO/H_2_O is also shown. **IIa** indicates the first two parallel resonances of the complex [Cu(L)] with [(O^–^, N, N^2^_im_); DMSO] binding mode and the dashed line indicates the distinctive resonance of the trinuclear species [Cu(L)]_3_.

**Table S5.** p*K*_a_ values of the studied ligand precursors determined by UV-Vis spectrophotometric titrations in 30% (v/v) DMSO/H_2_O in addition to their thermodynamic solubility (*S*_7.4_) and distribution coefficients (log*D*_7.4_) at pH 7.40 determined experimentally via *n*-octanol/water partitioning. [t = 25.0 °C; I = 0.1 M (KCl)].

|  | **L^2^** | **L^3^** | **L^4^** | **L^5^** | **L^6^** |
| --- | --- | --- | --- | --- | --- |
| **R =** | **Cl** | **F** | **CH_3_** | **OCH_3_** | **OH** |
| **p*K_a_*_1_** | 2.11 | 2.10 | 2.18 | 2.34 | 2.33 |
| **p*K_a_*_2_** | 9.39 | 9.45 | 9.61 | 9.61 | 8.29 |
| **p*K_a_*_3_** | 11.01 | 11.18 | 11.67 | 11.71 | 9.65 |
| **p*K_a_*_4_** | − | − | − | − | > 12 |
| ***S*_7.4_** (μM)**_._** | < 1 | < 1 | 1.7 | 2.0 | 2.1 |

**Figure** **S8.** a) UV-Vis spectra of L^8^ recorded at various pH values and b) calculated individual absorption spectra of species in the different protonation states. [c_L_ = 60 µM; pH = 1.5 ‒ 12.5; t = 25.0 °C; I = 0.10 M (KCl); ℓ = 1.0 cm; 30% (v/v) DMSO/H_2_O].

**Figure** **S9.** Concentration distribution curves for a) L^7^ and b) L^8^ together with the measured (●) and the fitted absorbance values at 325 nm (L^7^) and 289 nm (L^8^) at the different pH values. [c_L_ = 60 µM; t = 25.0 °C; I = 0.10 M (KCl); ℓ = 1.0 cm; 30% (v/v) DMSO/H_2_O].

**Figure** **S10.** a) UV-Vis spectra of the Cu(II) ‒ L^5^ (1:1) system recorded at various pH values. b) Calculated individual molar absorption spectra of the complexes. [c_Cu(II)_ = c_L_ = 50 µM; pH = 1.5 ‒ 11.6; t = 25 °C; I = 0.10 M (KCl); ℓ = 1.0 cm; 30% (v/v) DMSO/H_2_O].

**Figure** **S11.** (a) Concentration distribution curves for the hypothetic Cu(II) − VO(IV) − L^6^ (1:1:1) chemical system. [c_L_ = 50 µM; c_Cu(II)_ = 50 µM; c_V(IV)O_ = 50 µM; t = 25.0 °C; I = 0.10 M (KCl); ℓ = 1.0 cm; 30% (v/v) DMSO/H_2_O]*.* (b) pM values plotted against the pH calculated with the indicated concentration of the metal ions and the ligand precursor. Formation of mostly mono-ligand complexes of V(IV)O was found in the acidic pH range and additional bis-ligand complexes appeared at pH > 7.5 (15). At physiological pH, [VO(L)] is the predominating complex similarly to Cu(II). In the case of L^6^ the formation constants of the mono complexes with Cu(II) and V(IV)O ions can be directly compared showing lower values for the Cu(II) complexes. At the same time, oxidovanadium(IV) tends to hydrolyse stronger than Cu(II), thus, for the proper comparison of the affinities shown towards the two metal ions predominance curves were computed for the hypothetic system containing Cu(II), V(IV)O and L^6^ at equimolar concentrations (Fig. S11a). These concentration distribution curves reveal that the ligand has a stronger binding ability towards V(IV)O in the acidic pH range, whilst it binds Cu(II) stronger at pH > 7. The same finding is reflected in the pM values plotted against the pH (in b).

**Figure S12**. Optimized structures (B3LYP-D3/BS2 in SMD continuum model for water) for the possible Cu(II) complexes of L^6^. The values of Δ*G*_aq_ are reported in kcal mol^–1^.


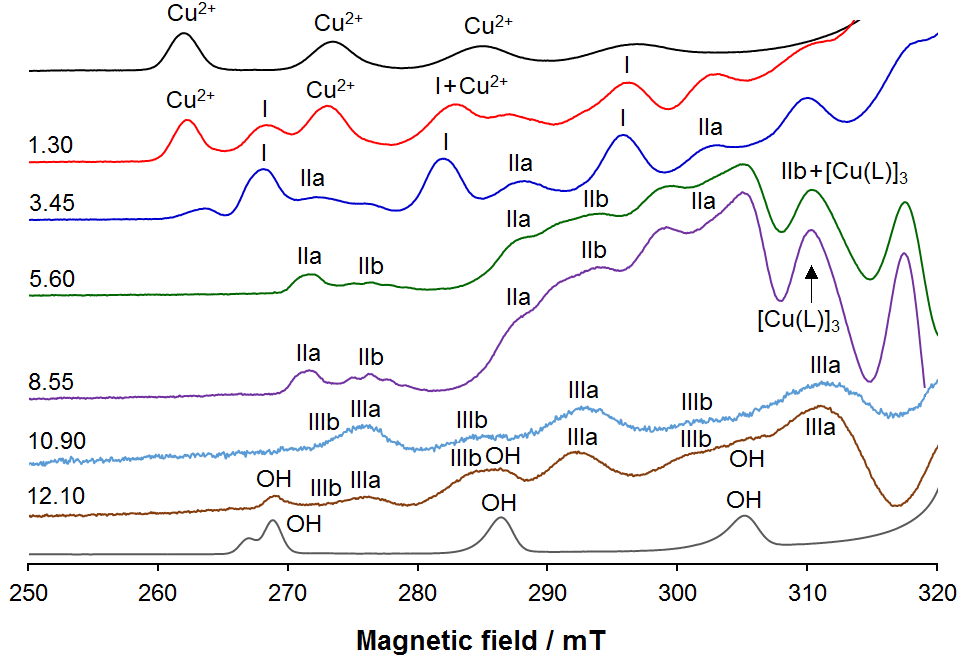


**Figure S13**. Low field region of the X-band anisotropic EPR spectra recorded as a function of pH at 120 K in a mixture DMSO/H_2_O 80/20 (v/v) containing ^63^Cu(II) and L^4^ (molar ratio 1:1 and Cu(II) concentration 1.0 mM). The first and last spectra were recorded on solutions containing the solvated and hydroxide complexes. With **I** are indicated the first three equatorial resonances of [Cu(LH)]^+^, with **IIa** and **IIb** of [Cu(L)] with the donor set [(O^–^, N, N^2^_im_); H_2_O] and [(O^–^, N, N^1^_im_); H_2_O], and with **IIIa** and **IIIb** of [Cu(LH_–1_)]^–^ with the binding sets [(O^–^, N, N^2^_im_); OH^–^] and [(O^–^, N, N^1^_im_); OH^–^]. With **Cu^2+^** and **OH** are denoted the resonances of solvated Cu^2+^ ion and hydroxido [Cu(OH)_4_]^2–^ complex, taken as references. Moreover, the position of the resonance distinctive of the trinuclear complex [Cu(L)]_3_ is also indicated by the arrow.

**Figure S14.** Normalized cyclic voltammograms recorded for the Cu(II) complexes **1**, **5** and **6**, against Ag/AgCl/3 M KCl reference electrode. [solvent: 90% DMSO-10% buffered aqueous solution: pH = 7.4 (10 mM HEPES); t = 25.0 °C; I = 0.10 M (TBAN); c_complex_ = 1 mM; scan rate: 15 mV/s; glassy carbon working and Pt counter electrodes].

**Figure S15.** Time-dependent changes of the absorbance values at 400 nm recorded for the Cu(II) complexes (**1**, **5**, **6**) in the presence of 100 equiv. GSH (filled symbols) or AA (unfilled symbols) at pH 7.4 in 5% (v/v) DMSO/H_2_O under anaerobic conditions. Inserted figures show the calculated observed rate constants (*k*_obs_). [c_complex_ = 40 µM; c_reducing agent_ = 4 mM; t = 25.0 °C; I = 0.10 M (KCl)].

**Figure S16.** Time-dependent changes of the ln(A/A_0_) at 400 nm recorded for the complex **5** in the presence of 100 equiv. GSH (●) at pH 7.4 in 5% (v/v) DMSO/H_2_O under anaerobic conditions, together with the fitted curve (solid line). [c_complex_ = 40 µM; c_reducing agent_ = 4 mM; t = 25.0 °C; I = 0.10 M (KCl)].


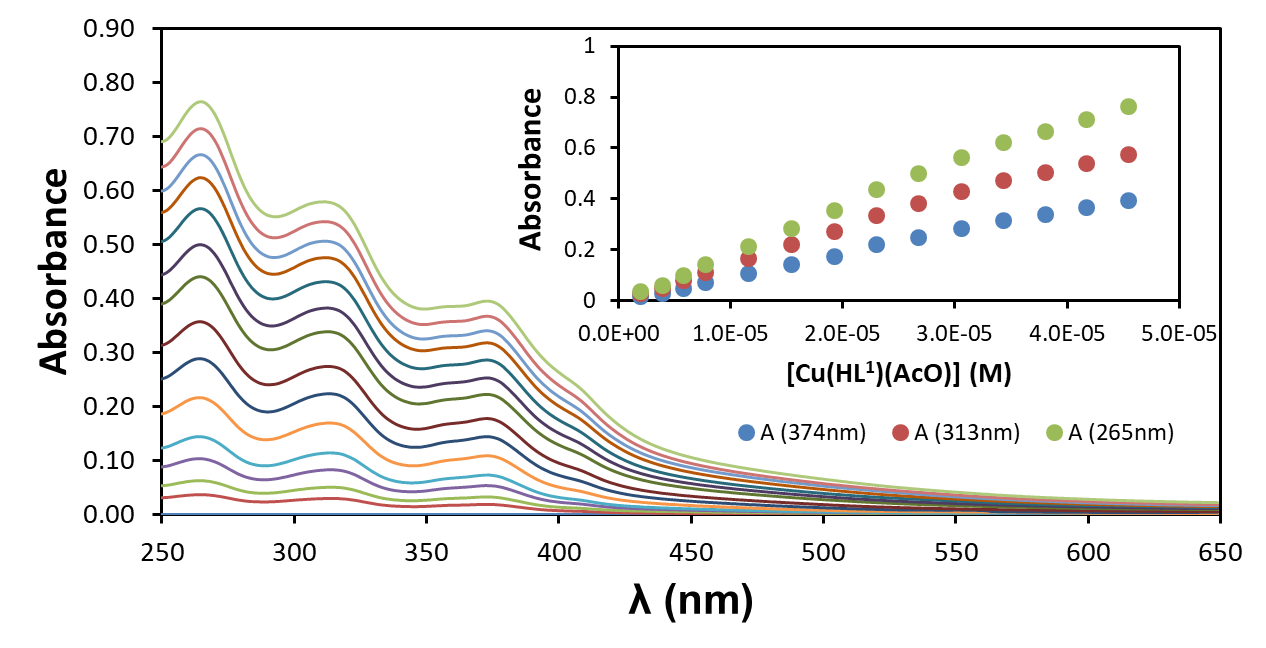


**Figure S17** – UV-Vis spectra of [Cu(HL^1^)(AcO)] (**1**) with increasing concentration in HEPES buffer (pH 7.4). The inset shows the variation of A *vs.* c at three different wavelengths.

| 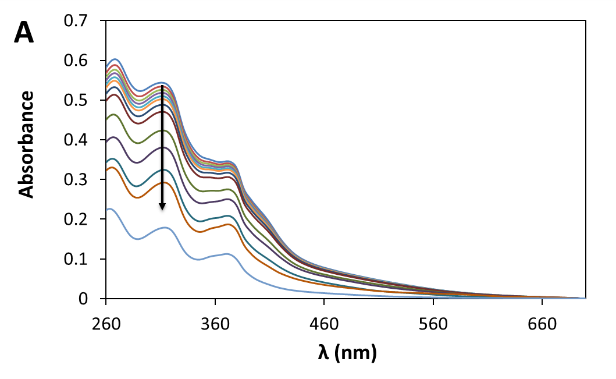 | 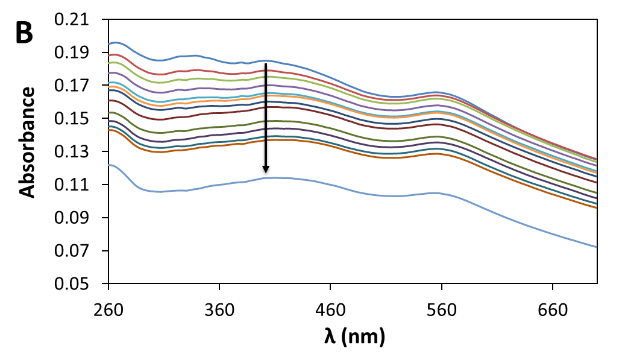 |
| --- | --- |
| 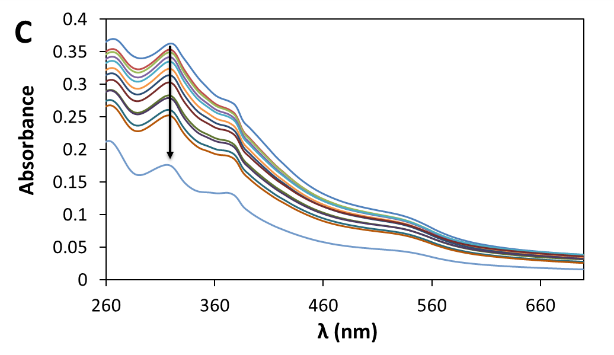 | 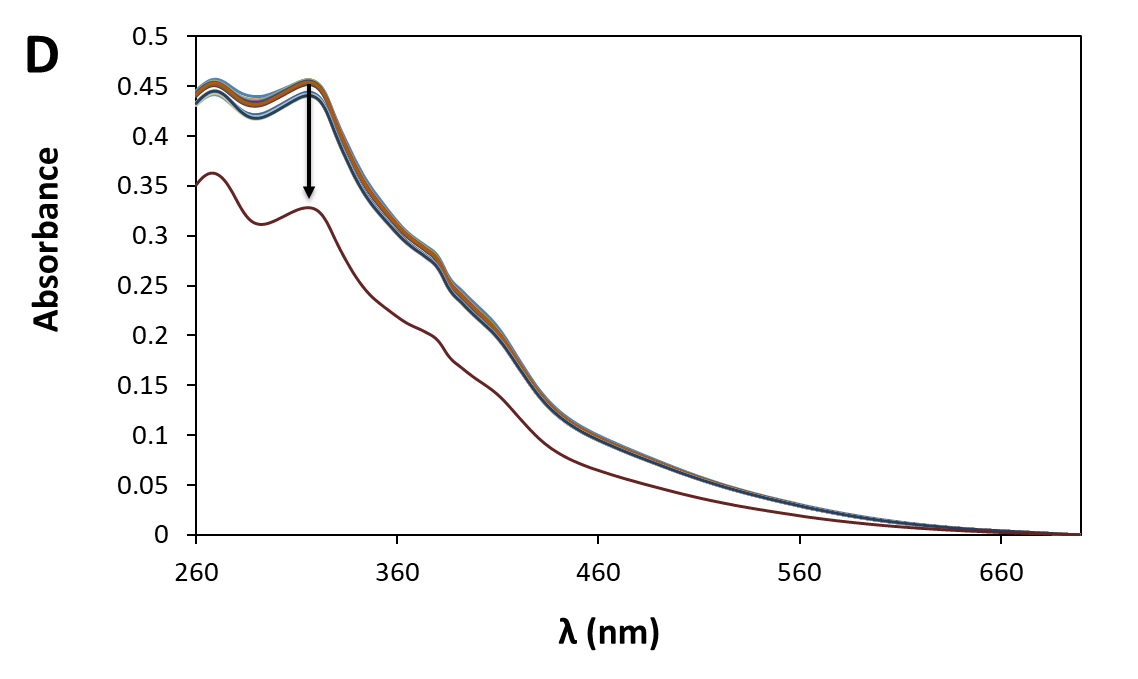 |
| 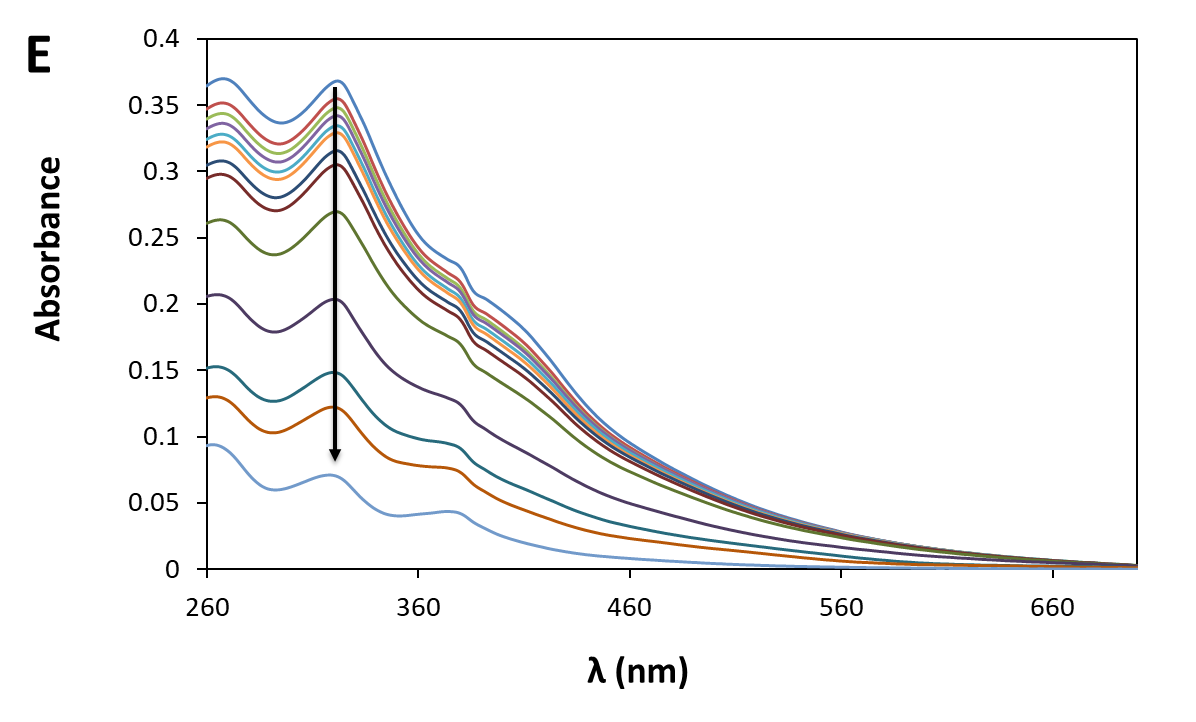 | 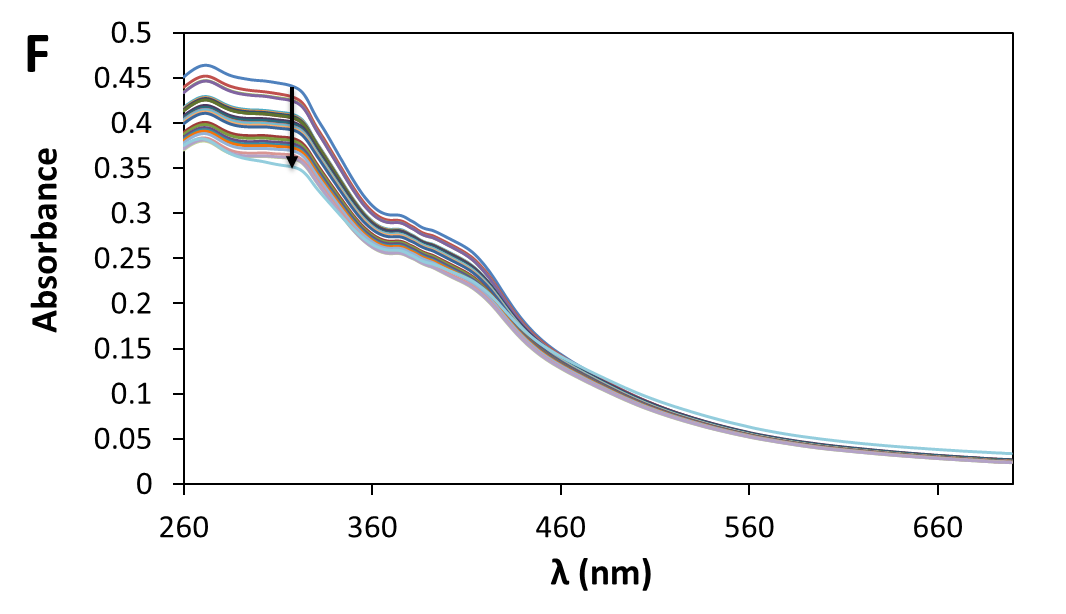 |
| 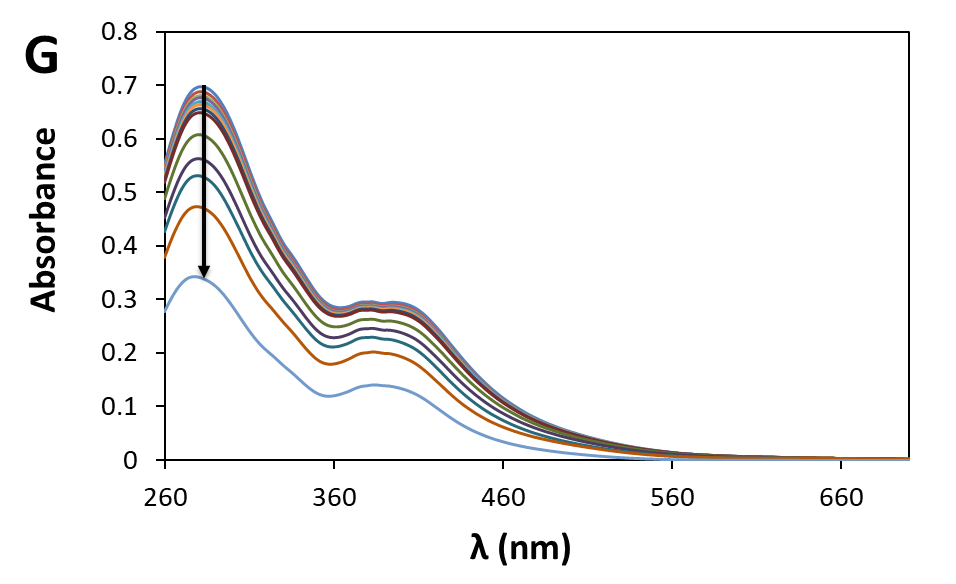 | 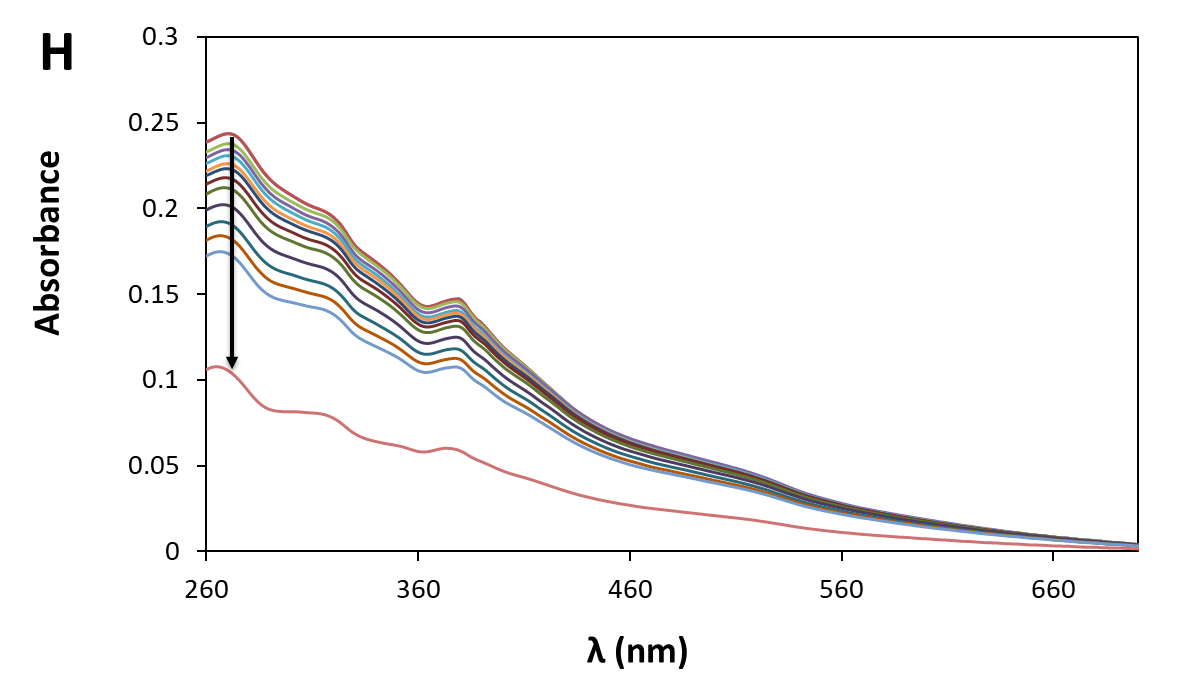 |
| 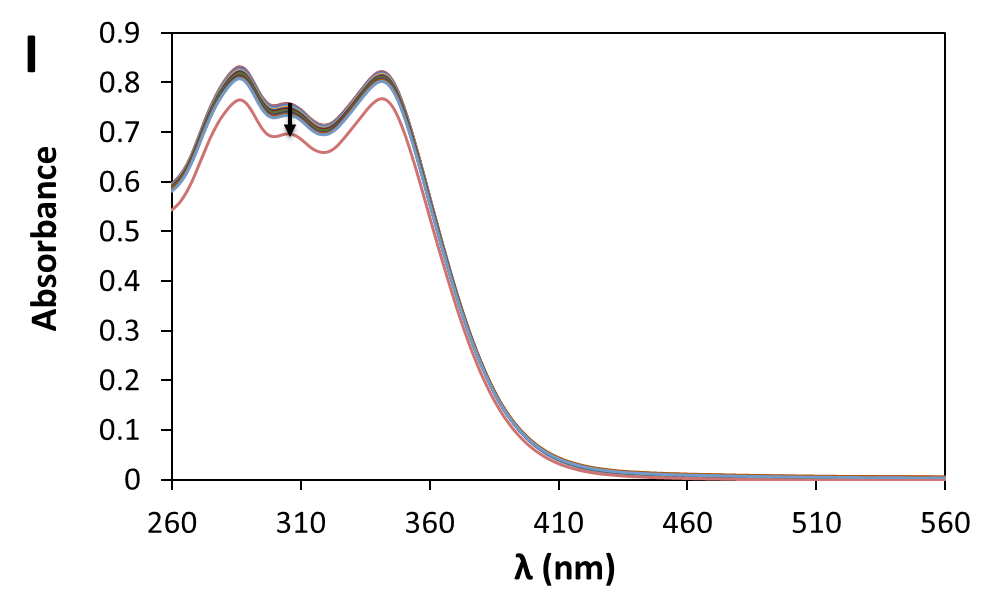 | 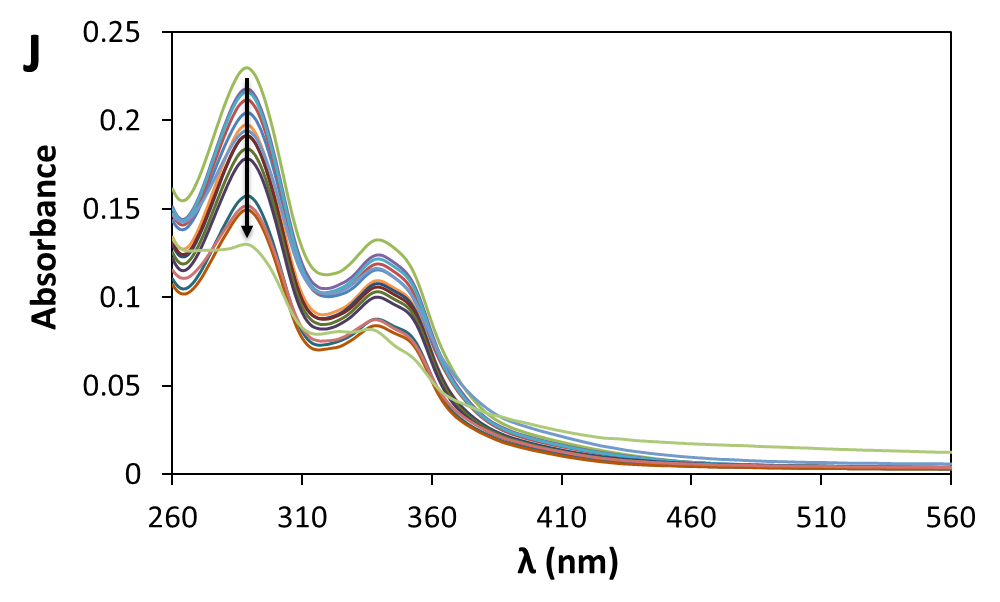 |

**Figure S18** – Stability of the compounds in aqueous HEPES buffer during 24 h (c ≈ 30 µM) followed by UV-Vis absorption spectroscopy, within 24 h. A – [Cu(HL^1^)(AcO)] (**1**), B – [Cu(L^2^)]_3_ (**2**), C – [Cu(L^3^)]_3_ (**3**), D – [Cu(HL^4^)(AcO)] (**4**), E – [Cu(L^5^)]_3_ (**5**), F – [Cu(L^6^)]_3_ (**6**), G – [Cu(L^7^)]_3_ (**7**), H – [Cu(L^8^)]_3_ (**8**), I – L^7^ (*ca.* 20 µM) and J – L^8^ (*ca.* 20 µM).

**S4 – Biological Interactions**

**S4.1. Binding to bovine serum albumin (BSA)**

Serum albumins are highly abundant in blood plasma and act as transporters of endogenous and exogenous compounds. The analysis of the interaction of newly synthesized compounds with bovine serum albumin (BSA) is the first model in the evaluation of their transport in blood, upon administration. BSA is commonly used in these studies due to its low cost and high homology to the human protein. Additionally, it is present in relatively high concentration in mammalian cells *in vitro* media (22-24) and allows increasing the drug solubility due to binding to the protein. Furthermore, the BSA drug conjugate may also be uptaken *p.e.* by endocytosis.

The interaction of the organic compounds and Cu(II) complexes with BSA was monitored by studying the quenching of albumin’s fluorescence emission with increasing concentrations of the complexes (25). Overall, the intensity of the Trp emission decreases with the addition of the complexes, except for **8** (see **Figs. S19**). The Stern-Volmer relationship was used to analyse the fluorescence quenching process:

${I_{0}}/I=1+ k_{q}\tau_{0}\left[ Q \right]$ (S5)

where *I_0_* and *I* are the fluorescence intensities in the absence and in the presence of quencher, *k_q_* is the bimolecular quenching rate, τ_0_ is the fluorophore lifetime in the absence of the quencher (6.22 ns) (26) and [*Q*] is the quencher concentration. In these studies it is assumed that the complexes do not dissociate during the time frame of measurements or upon binding, so that this methodology may be applied (22). This linear relationship is obeyed for all complexes except **5**, where a downward curvature is observed. This can be an indication of two distinct binding sites, corresponding to two populations of fluorophores, one being accessible and other inaccessible or buried inside the protein. In such cases, the Stern-Volmer relation can be modified to (27):

${I_{0}}/{\Delta I}= 1/{\left( f_{a}K_{a}\left[ Q \right] \right)+1/{f_{a}}}$ (S6)

where Δ*I* = *I_0_* – *I*, *K_a_* is the Stern-Volmer constant for the accessible fraction and *f_a_* is the fraction of accessible fluorophore. For the system BSA-**5**, *f_a_* is only 0.24 but still the quenching efficiency is quite high, with *K_a_* = 2.1 × 10^6^ M^−1^. As first approximation, the total concentration of quencher was used to check the quenching mechanism. All systems presented values for *k_q_* (or *K_a_* in the case of BSA-**5**) above the limit for diffusional quenching (27), suggesting the formation of a new species upon binding of the quencher to the protein. As all systems presented high absorbance values in the measured range of wavelengths, no excess of quencher was used and, therefore, 1:1 species will be assumed for these interactions. HypSpec2014 (28) was used to determine the affinity constants for the different compounds and the results are presented in **Table S6**.

| 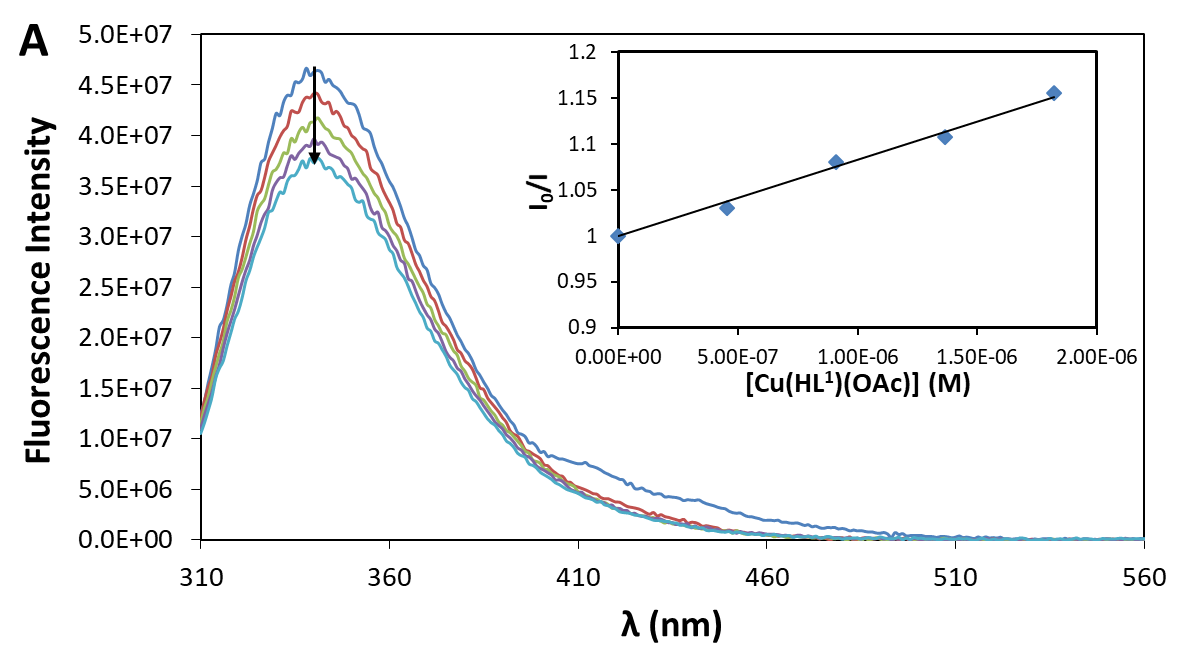 | 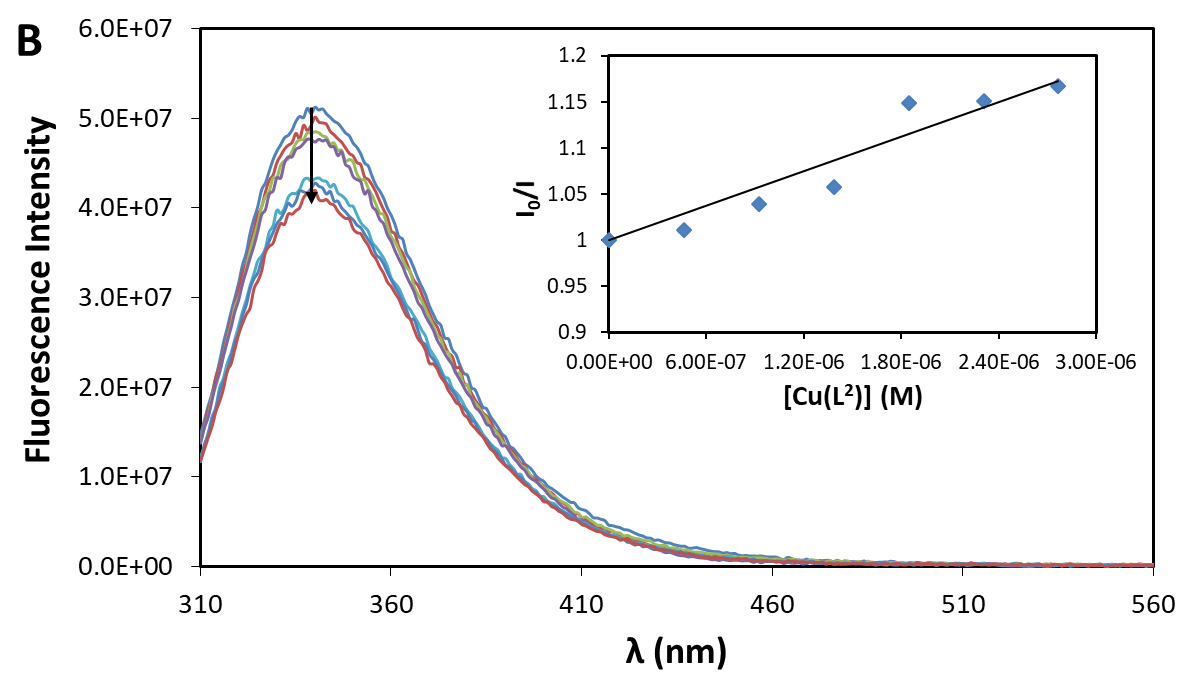 |
| --- | --- |
| 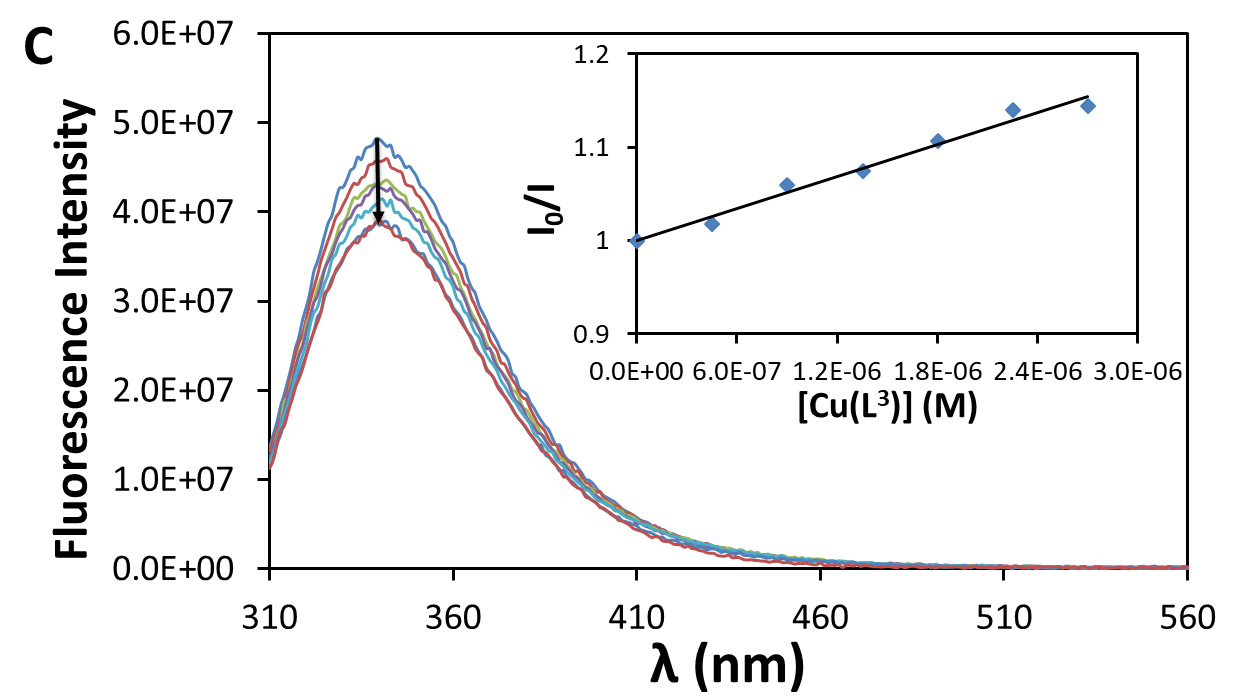 | 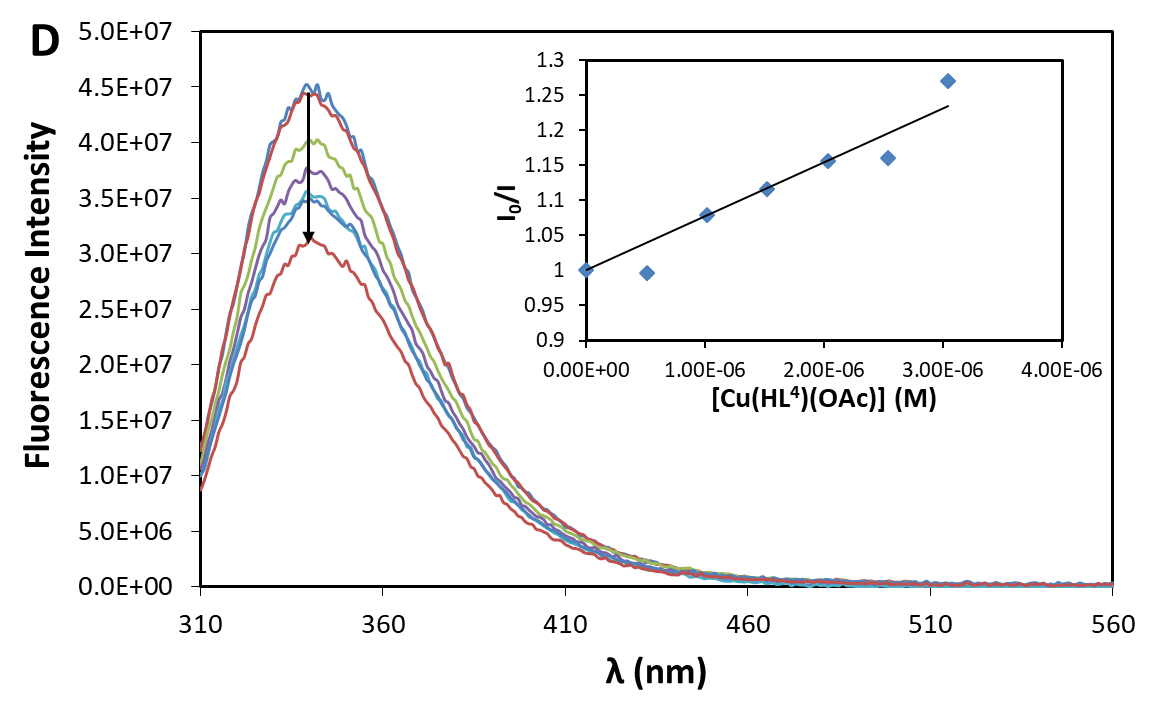 |
| 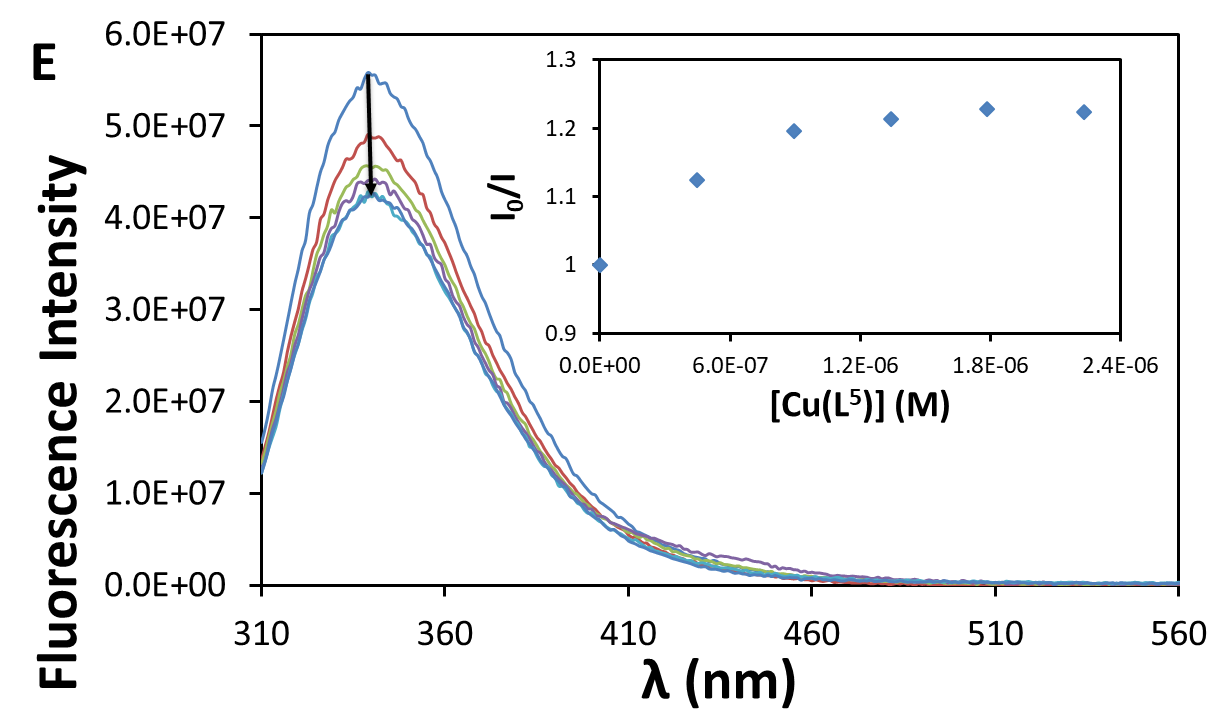 | 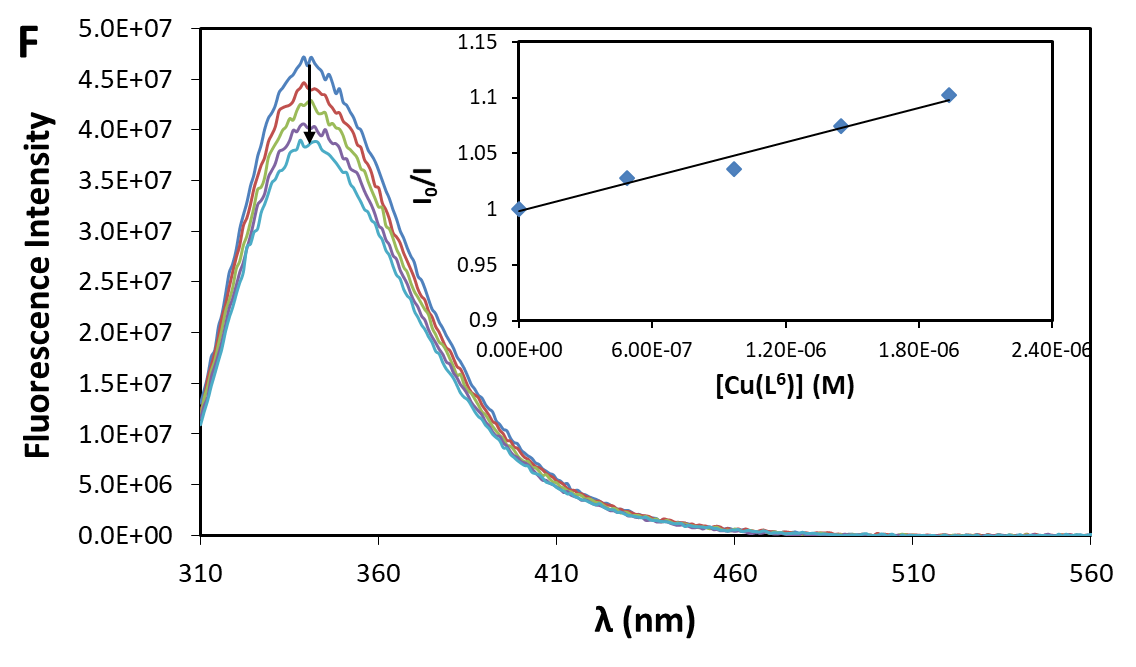 |
| 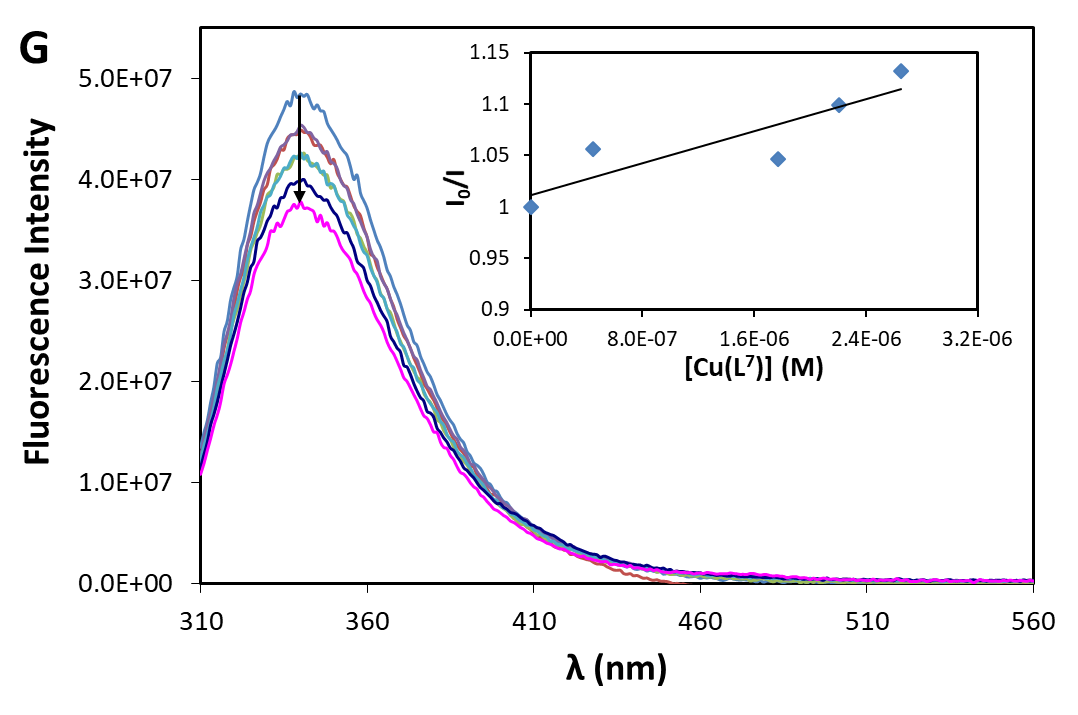 | 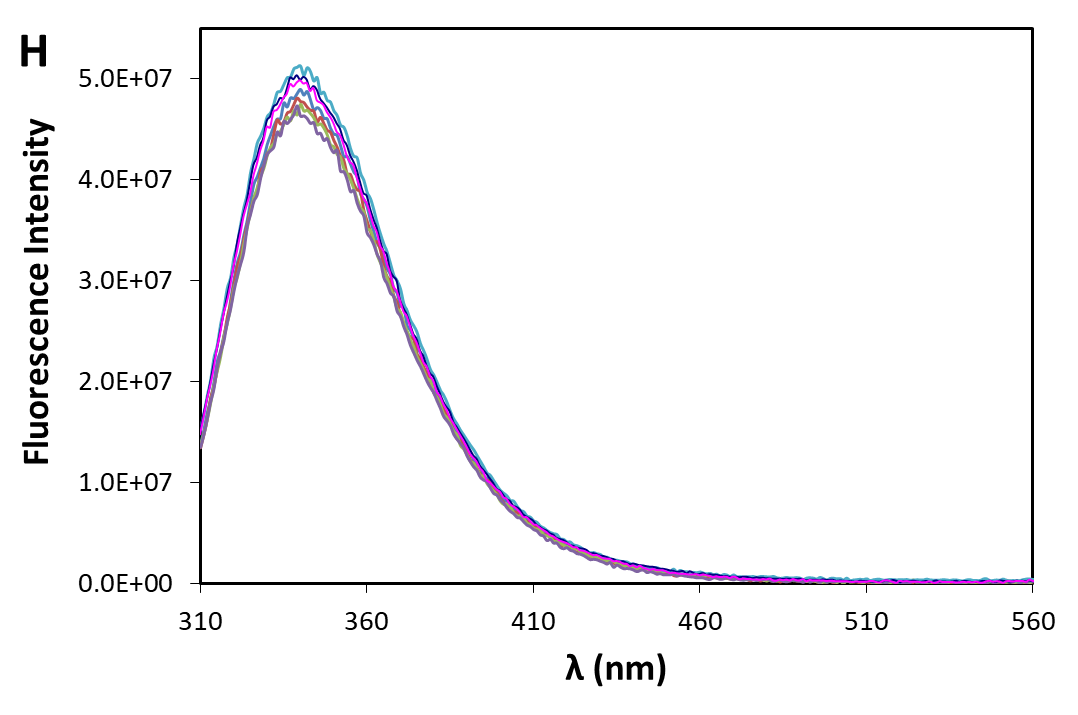 |

**Figure S19** –Fluorescence emission quenching experiments with BSA (*ca.* 1.5 µM) and increasing amounts of each complex. *Inset*: Stern-Volmer plot corresponding to each experiment. (**A**) [Cu(HL^1^)(AcO)] (**1**), (**B**) [Cu(L^2^)] (**2**), (**C**) [Cu(L^3^)] (**3**), (**D**) [Cu(HL^4^)(AcO)] (**4**), (**E**) [Cu(L^5^)] (**5**), (**F**) [Cu(L^6^)] (**6**), (**G**) [Cu(L^7^)] (**7**) and (**H**) [Cu(L^8^)] (**8**).

**Table S6**. Affinity constants (*K*), obtained from the fluorescence quenching experiments between bovine serum albumin (BSA) and each compound with computer software HypSpec2014.

| **K ± SD** | | | |
| --- | --- | --- | --- |
| **1** | (8.4 ± 6.6) × 10^7^ | L^1^ | (4.6 ± 0.2) × 10^5^ |
| **2** | (8.3 ± 0.1) × 10^4^ | L^2^ | (1.8 ± 0.1) × 10^5^ |
| **3** | (3.4 ± 0.7) × 10^5^ | L^3^ | (1.6 ± 0.1) × 10^6^ |
| **4** | (3.2 ± 1.0) × 10^6^ | L^4^ | (1.1 ± 0.1) × 10^6^ |
| **5** | (1.05 ± 0.04) × 10^5^ | L^5^ | (2.1 ± 1.9) × 10^6^ |
| **6** | (2.7 ± 0.8) × 10^5^ | L^6^ | (1.9 ± 0.2) × 10^5^ |
| **7** | (3.4 ± 0.2) × 10^4^ | L^7^ | (6.3 ± 7.3) × 10^6^ |
|  |  | L^8^ | (1.1 ± 0.4) × 10^6^ |

SD – Standard Deviation

Overall, the fluorescence titrations showed moderate to strong interaction between BSA and the synthesized complexes, suggesting the formation of a ground state complex in a static mechanism of quenching. Some of the determined affinity constants present high associated errors, which can be an indication of the failure of the chosen model. Considering the dimensions and adaptability of the BSA molecule, one hypothesis is for the binding of more than just one molecule of quencher in those situations.

## S4.2. Interaction with DNA

Nucleic acids are potential targets for metallodrugs and, consequently, evaluation of the interactions of the compounds with DNA and corresponding binding strength is relevant under the scope of this work. Thermal denaturation is a useful technique to measure the stability of the secondary structure of DNA (29). Under the chosen experimental conditions (HEPES, 0.01 M, pH 7.4), it is possible to follow the denaturation of *calf thymus* DNA (*ct*DNA) double strand. The melting curve presents a well-defined midpoint and the sigmoidal fit yields T_m_ = 60.3 ± 0.6 °C (**Fig. S20**). Selected compounds: L^1^, L^3^, L^5^, L^7^, and their corresponding Cu(II) complexes were tested and found to increase the melting temperature of DNA (13.0 °C < ΔT_m_ < 26.8 °C; **Fig. S21**) except for the compounds bearing the amino substituent. The melting curves (A_260nm_ *vs.* T) show similar profiles for the organic compounds and corresponding Cu(II) complexes, suggesting that the effect is mediated by the organic moiety. The amino derivatives L^7^ and **7** decrease the melting temperature of DNA by *ca.* 3 °C, lowering the energy required to break the double helix into single strands (**Table S7**). Intermolecular forces do not explain this behaviour as both L^3^ and L^7^ can establish hydrogen bonds with DNA.


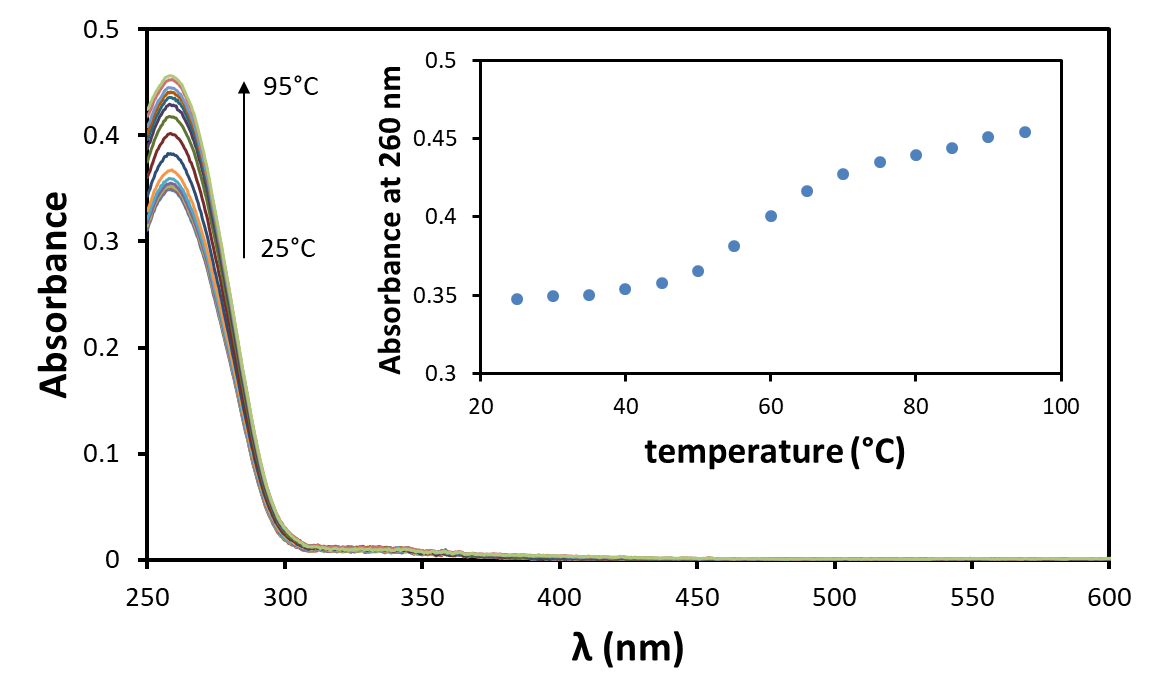


**Figure S20** – UV-Vis absorption spectra of *ct*DNA with increasing temperature. The inset shows the S-shaped curve variation at the maximum of absorbance (260 nm). The melting temperature (T_m_ = 60.2 ± 0.6°C) is obtained from the inflection point.

| 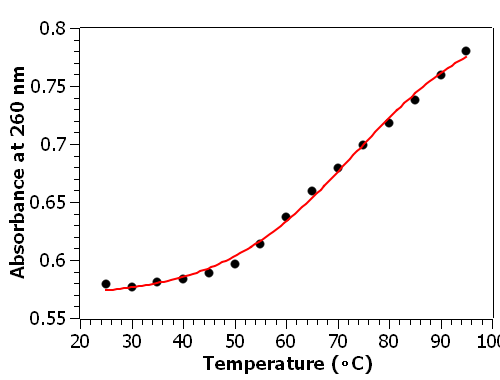 | 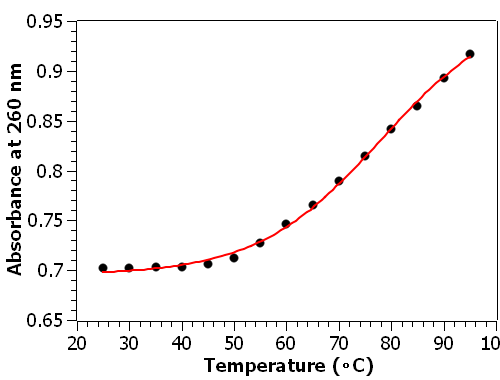 |
| --- | --- |

**Figure S21** – Absorbance changes at 260 nm for equimolar mixtures (25 µM) of *ct*DNA-**3** (left) and *ct*DNA-**5** (right) with increasing temperature. Black dots: experimental points; red lines: sigmoidal fits. The spectra were measured in 10 mM HEPES aqueous buffer (pH 7.4) with 1.5% DMSO (v/v). The melting temperature (T_m_) obtained from the inflection point of the sigmoidal fit gave T_m_ = 73.2 ± 1.7 °C and 77.9 ± 1.3 °C for *ct*DNA-**3** and *ct*DNA-**5**, respectively.

**Table S7** – Temperature differentials (ΔT_m_ in °C) for the DNA melting temperature in contact with equimolar amounts of selected compounds.

| Compound | ΔT_m_ (°C) |
| --- | --- |
| L^1^ | 2.2 |
| L^3^ | 16.2 |
| L^5^ | 6.7 |
| L^7^ | −3.9 |
| 1 | >25.0 |
| 3 | 13.0 |
| 5 | 17.7 |
| 7 | −2.5 |

The results obtained for the unsubstituted ligand (L^1^) show that the organic compound almost does not interact with DNA - small change in the T_m_ value and no fluorescence quenching – while its Cu-complex (**1**) increases the DNA melting temperature with a ΔT_m_ > 25.0 °C. Moreover, this complex reduces the emission of the EB-DNA system to *ca.* 40 % of its initial intensity with an interaction constant of (1.18 ± 0.03) × 10^4^ M^−1^ (**Fig. S22**, constant calculated with HypSpec2014 (28)).

| 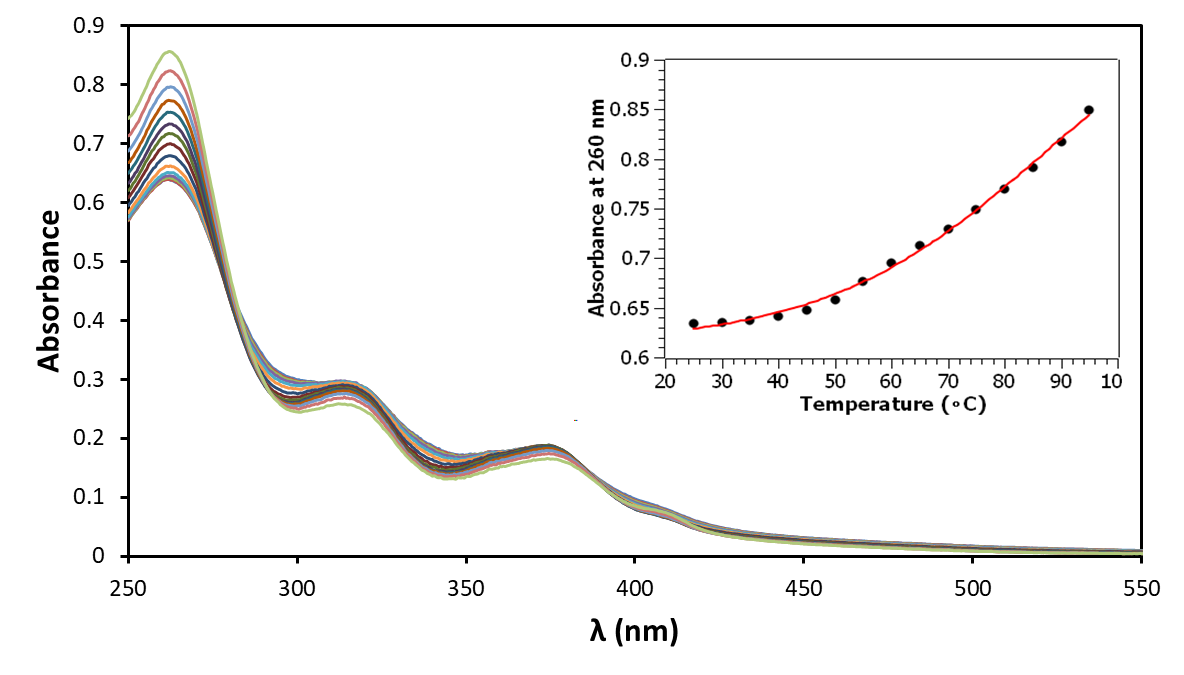 | 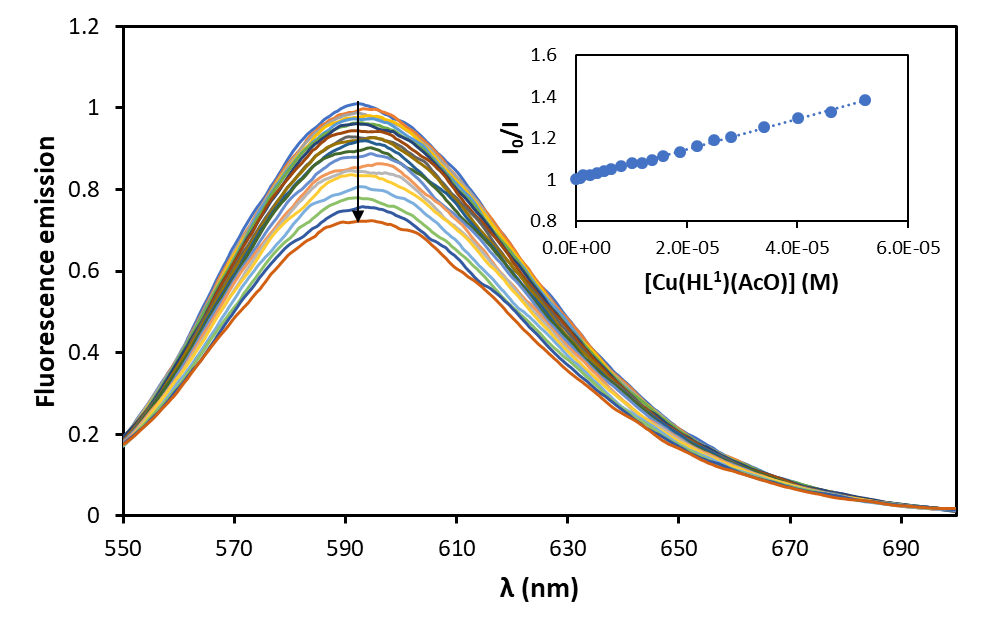 |
| --- | --- |

**Figure S22** – Left: UV/Vis spectra of a solution containing 19.2 µM of [Cu(HL^1^)(AcO)] (**1**) and 20.9 µM of DNA (in base pairs) in 10 mM HEPES (pH 7.4) with increasing temperature (25, 30, 35, 40, 45, 50, 55, 60, 65, 70, 75, 80, 85, 90, and 95 °C). *Inset*: Variation of the absorbance at 260 nm with the inflection point showing the melting temperature - T_m_. Right: Fluorescence emission spectra of the system DNA-EB ([DNA] = 20.8 µM and [EB] = 30 µM) titrated with [Cu(HL^1^)(AcO)] (**1**) (0 – 51.6 µM) in buffer: HEPES, 0.01 M, 0.1 M KCl, pH 7.4. *Inset*: Stern-Volmer plot (*I_0_*/*I* vs. concentration) at 590 nm.

Considering the results of the melting temperature assay, the DNA-EB fluorescence quenching was also used to test complex **7**. The Stern-Volmer analysis showed a downward curvature and the modified equation (S6), was used in this case (**Fig. S23A**). The accessible fraction *f_a_* was determined as 0.26 and the interaction constant as (1.44 ± 0.02) ×10^5^ M^−1^ (HypSpec2014 (28)). As EB has a second mode of interaction with DNA due to electrostatic interactions on the external part of the biomolecule backbone (30), it is possible that the complexes are only being able to reach for this fraction. L^3^ and L^7^ were also tested along with L^1^ and none of the compounds was able to displace EB from its interacting site (**Fig. S23B-D**). Overall, the results point to weak interactions of all compounds with DNA and, most likely, occurring at the external surface of the biomolecule.

| **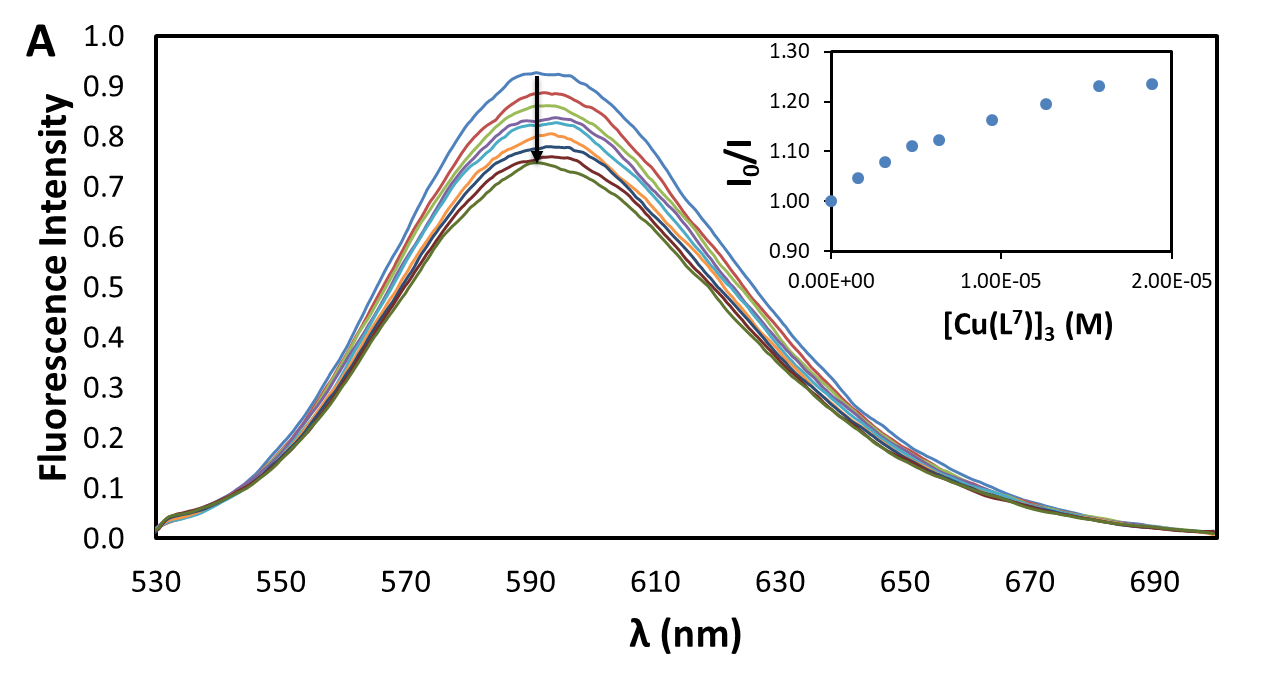** | **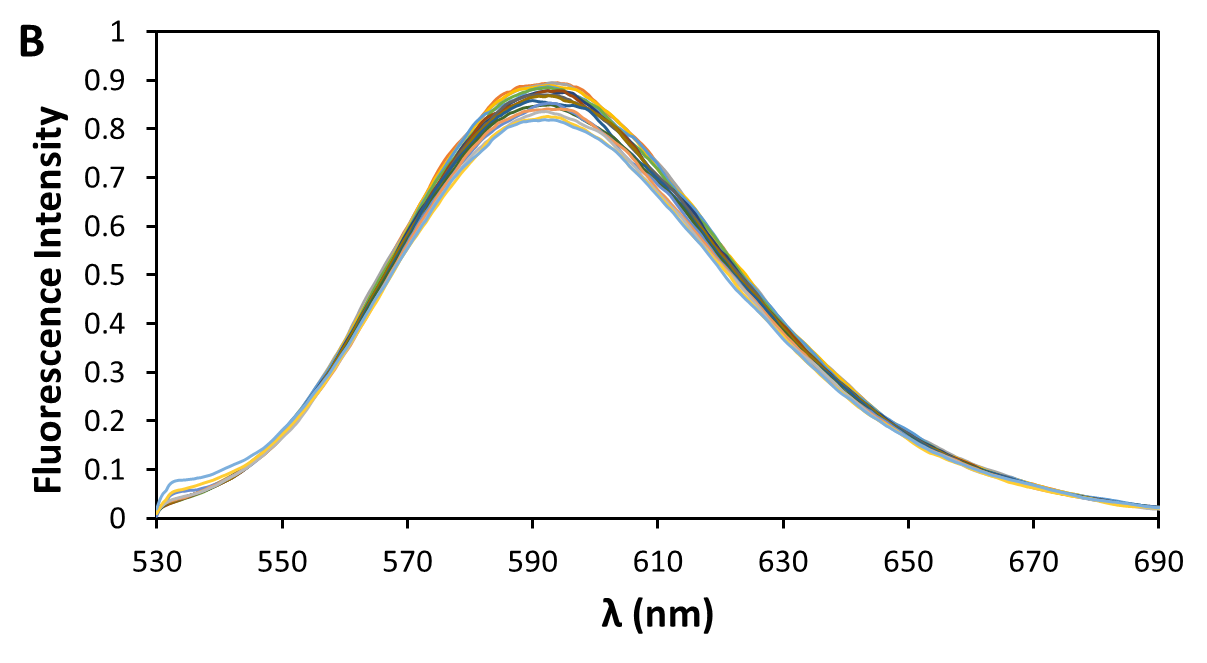** |
| --- | --- |
| **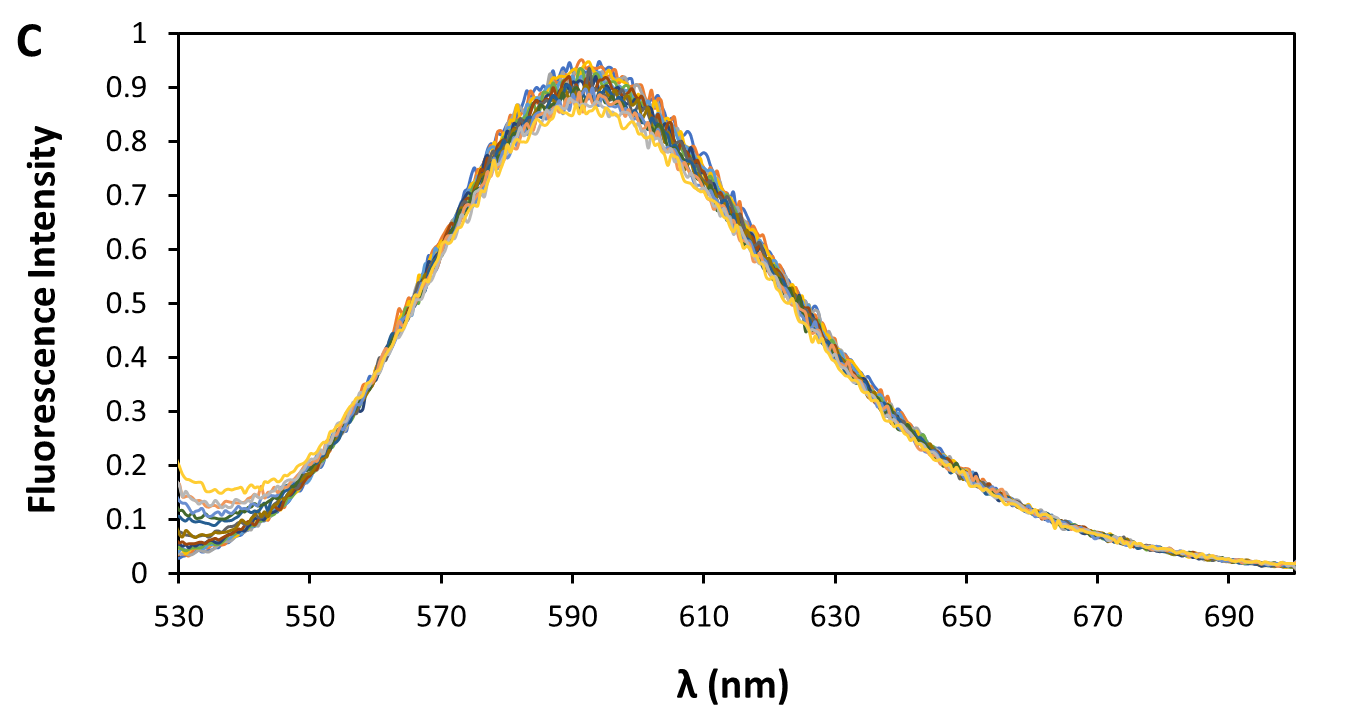** | **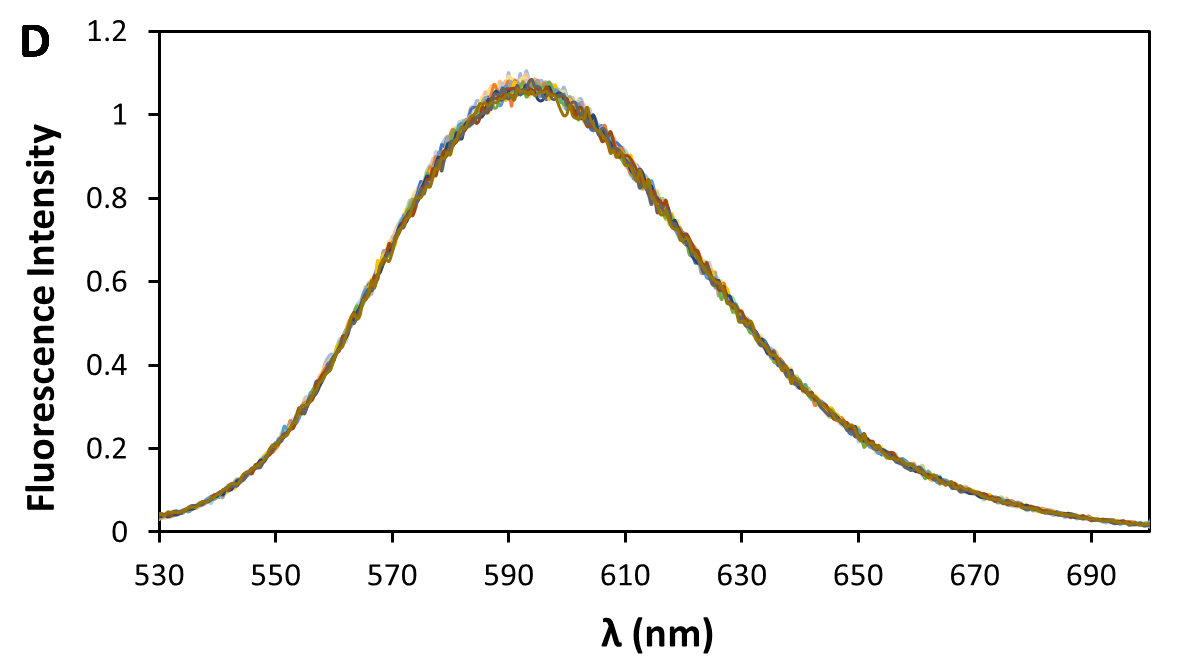** |

**Figure S23** –Fluorescence emission spectra of the system DNA-EB ([DNA] = 20.8 µM and [EB] = 30 µM) titrated with (**A**) [Cu(L^7^)]_3_ (**7**) (0 – 20 µM), (**B**) L^7^ (0 – 74.5 µM), (**C**) L^3^ (0 – 52 µM), and (**D**) L^1^ (0 – 54.2 µM) in buffer: HEPES, 0.01 M, 0.1 M KCl, pH 7.4. *Inset in (A)*: Stern-Volmer plot (*I_0_*/*I* vs. concentration) at 590 nm.

***S4.3. In vitro* DNA migration assay**

In addition to thermal denaturation, we performed a cell-free DNA binding assays followed by gel electrophoresis to further investigate the nature of the interaction between the complexes and DNA. The assay relies on the fact that the relative amount of the supercoiled form of plasmid DNA will change upon the occurrence of nicks since the relaxed form migrates slower and can be easily detected on the gel. On the other hand, an intercalating agent, which does not induce nicks or breaks, leads to faster migration, usually as a result of more compact plasmid, due to additional intra- or inter-stand DNA bonds (a typical example is cisplatin). The ligands had little to no effect on DNA migration except L^7^ (**Fig. S24A**). However, incubation of plasmid DNA with complexes **1** - **8** confirmed binding to DNA and changed the supercoiling status by increasing the nicked circular form (**Fig. S24**). The most dramatic response was obtained with **1**, with the highest nicking efficiency. To understand whether the induction of DNA nicks requires ROS, the samples were pre-incubated with a ROS scavenger, NaN_3_. The change in DNA migration was reversed, suggesting that free radical formation induced by the complexes was the main drive for the observed effect. Overall, the complexes appeared as promising complexes with little activity of their corresponding ligands.

**
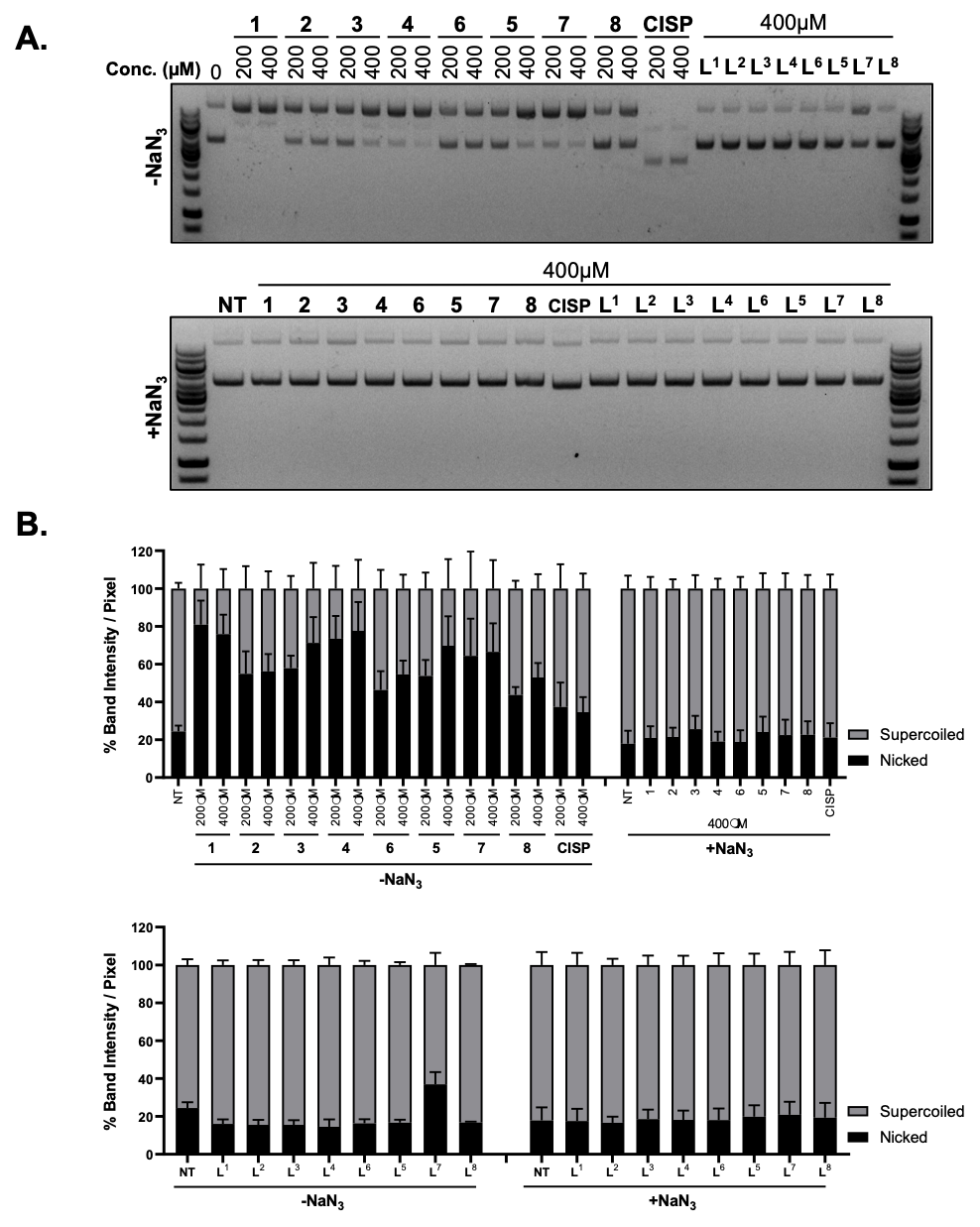
**

**Figure S24** – Gel electrophoresis of plasmid DNA treated with copper complexes **1**-**8** and their corresponding ligands. Cisplatin (CISP) served as positive control. Plasmid DNA was incubated with different concentrations of compounds (200 and 400 μM) overnight at room temperature. NaN_3_ was used as a ROS scavenger. The intensity of the bands in each lane was quantified and is represented as Mean ± Standard deviation. A) Representative gel images (top gel: -NaN_3_ condition, bottom gel: +NaN_3_ condition). B) Quantitative analysis of band intensities (Top: Cu complexes, Bottom: ligands)

**S5 – Cell studies**


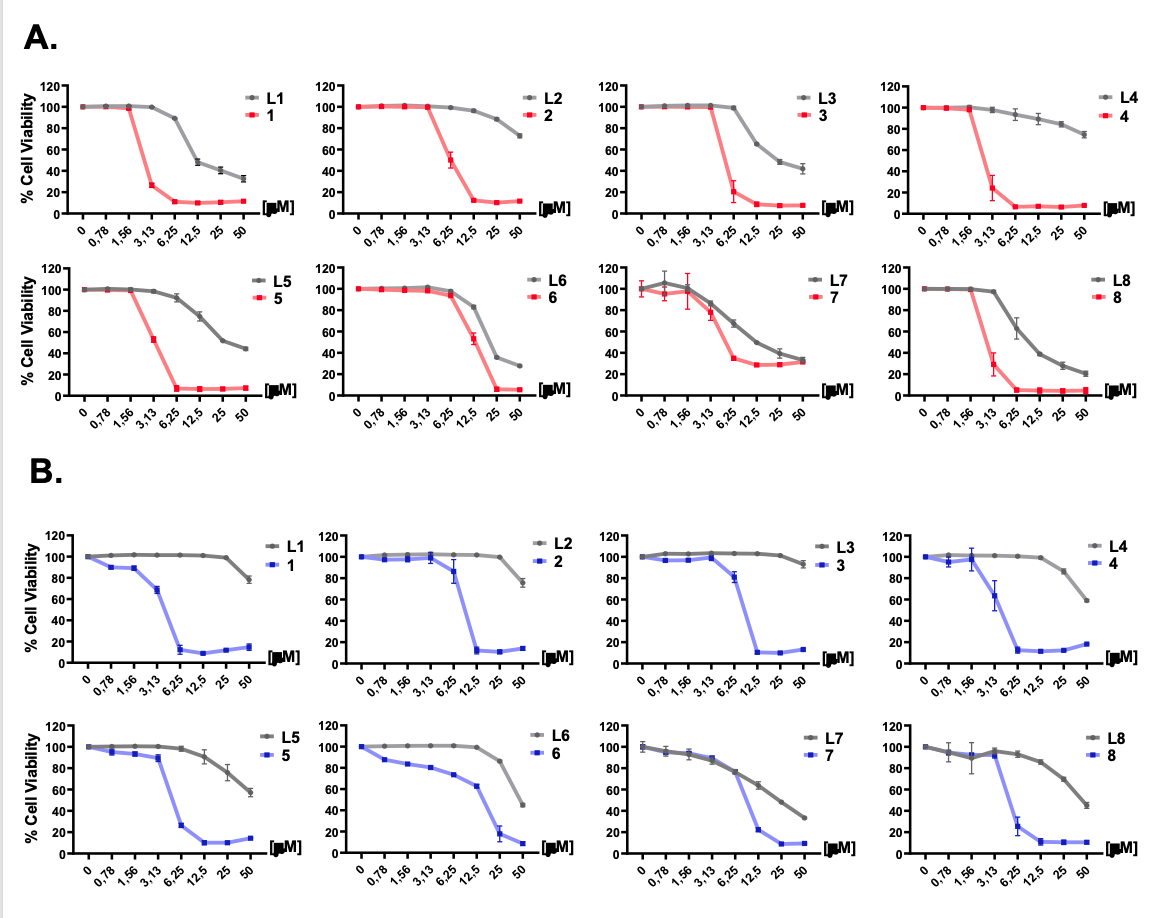


**Figure S25.** Cell viability results as determined by *sulforhodamine B (SRB) assay:* (**A**) A-375 and (**B**) A-549 cells were exposed to selected copper complexes (**1**, **2**, **3**, **4**, **5**, **6**, **7**, and **8**) and corresponding ligands for 72h. Error bars represent the standard deviation from the mean.

**Figure S26** *-* Induction of oxidative stress in response to treatment with the Cu complexes. Activation of reactive oxygen species upon treatment with cisplatin (CISP) or **1**, **3**, **5**, **8** on the A-549 cells. ROS activation was assessed via dihydroethidium staining.

**Figure S27-** Protective mechanism of NAC against Cu(II) complexes. Addition of NAC can protect cells from the cytotoxic effects of the Cu(II) complexes A-375 (**A**) and A-549 (**B**) cells were pre-treated with 10 mM of ROS scavenger N-acetylcysteine (NAC) for 2 h, then co-treated with freshly prepared Cu(II) complexes (**1**, **3**, **5** and **8**) for 72 h. Cell viability was measured via SRB assay (2 biological replicates, each done in duplicate). The error bars represent standard error of the mean. Student t-test was used for statistical analysis, *p<0.05, **p<0.01

**A**

**B**

**Figure S28** – Induction of apoptosis upon treatment with the Cu-complexes. Flow cytometric analyses of (**A**) Annexin V / 7-AAD positivity and **(B)** Caspase 3/7 activity on A-549 cells. Cells were exposed to **1**, **3**, **5**, **8** or cisplatin at half inhibitory concentrations for 48 h, and counted with Muse Cell Analyzer.

**Table S8.** MIC values determined for the ligands in the absence and presence of one equiv. Cu(II) on various bacteria.^a^

| **MIC** (μM) | ***S. aureus*** *ATCC 25928* | ***S. aureus*** *MRSA* | ***E. coli*** | ***K. pneumoniae*** |
| --- | --- | --- | --- | --- |
| **Ligand precursors** | > 100 | > 100 | > 100 | > 100 |
| **1** | 50 | 50 | > 100 | > 100 |
| **3, 5** | 100 | 100 | > 100 | > 100 |
| **6, 8** | > 100 | > 100 | > 100 | > 100 |
| **7** | 12.5 | 50 | > 100 | > 100 |
| **CuCl_2_** | > 100 | > 100 | > 100 | > 100 |

^a^ The applied amount of the solvent DMSO had no antibacterial effect.

**References**

1. *Bruker Apex3, Crystallography Software Suite, Bruker Axs Inc., Madison, Wi, USA*. USA(2016).

2. Bruker Axs: Saint+, Release 6.22. *Bruker AXS:SAINT+, release 622; Bruker Analytical Systems: Madison,WI* (2005).

3. Bruker Axs:Sadabs. *Bruker AXS:SADABS; Bruker Analytical Systems: Madison,WI* (2005).

4. Sheldrick GM. Shelxt - Integrated Space-Group and Crystal-Structure Determination. *Acta Crystallographica a-Foundation and Advances* (2015) 71:3-8. doi: 10.1107/s2053273314026370.

5. Sheldrick GM. Crystal Structure Refinement with Shelxl. *Acta Crystallographica Section C-Structural Chemistry* (2015) 71:3-8. doi: 10.1107/s2053229614024218.

6. Farrugia LJ. *Wingx* Suite for Small-Molecule Single-Crystal Crystallography. (1999) 32:837-8.

7. Spek AL. *Acta Crystallographica Section C* (2015) 71:9-18.

8. Macrae CF, Sovago I, Cottrell SJ, Galek PTA, McCabe P, Pidcock E, et al. Mercury 4.0: From Visualization to Analysis, Design and Prediction. *J. Appl. Cryst.* (2020) 53:226-35. doi: https://doi.org/10.1107/S1600576719014092.

9. Ribeiro N, Bulut I, Pósa V, Sergi B, Sciortino G, Costa Pessoa J, et al. Solution Chemical Properties and Anticancer Potential of 8-Hydroxyquinoline Hydrazones and Their Oxidovanadium(Iv) Complexes *J. Inorg. Biochem*. (2022) 235:111932.

10. Irving HM, Miles MG, Pettit LD. A Study of Some Problems in Determining the Stoicheiometric Proton Dissociation Constants of Complexes by Potentiometric Titrations Using a Glass Electrode. *Anal. Chim. Acta.* (1967) 38:475-88. doi: https://doi.org/10.1016/S0003-2670(01)80616-4.

11. Enyedy ÉA, May NV, Pape VFS, Heffeter P, Szakács G, Keppler BK, et al. Complex Formation and Cytotoxicity of Triapine Derivatives: A Comparative Solution Study on the Effect of the Chalcogen Atom and Nh-Methylation. *Dalton Trans.* (2020) 49(46):16887-902. doi: 10.1039/D0DT03465G.

12. Zékány L, Nagipál I. Psequad, a Comprehensive Program for the Evaluation of Potentiometric and/or Spectrophotometric Equilibrium Data Using Analytical Derivatives. *Computational Methods for the Determination of Formation Constants*. Springer US, Boston, MA (1985). p. 291-353.

13. Frisch MJ, Trucks GW, Schlegel HB, Scuseria GE, Robb MA, Cheeseman JR, et al. *Gaussian 16 Rev. B.01*. Wallingford, CT(2016).

14. Stefan G, Jens A, Stephan E, Helge K. A Consistent and Accurate Ab Initio Parametrization of Density Functional Dispersion Correction (Dft-D) for the 94 Elements H-Pu. *J. Chem. Phys.* (2010) 132(15):154104. doi: 10.1063/1.3382344.

15. Ehlers AW, Böhme M, Dapprich S, Gobbi A, Höllwarth A, Jonas V, et al. A Set of F-Polarization Functions for Pseudo-Potential Basis Sets of the Transition Metals Sc, Cu, Y, Ag and La, Au. *Chem. Phys. Lett.* (1993) 208(1):111-4. doi: https://doi.org/10.1016/0009-2614(93)80086-5.

16. Marenich AV, Cramer CJ, Truhlar DG. Universal Solvation Model Based on Solute Electron Density and on a Continuum Model of the Solvent Defined by the Bulk Dielectric Constant and Atomic Surface Tensions. *J. Phy. Chem. B* (2009) 113(18):6378-96. doi: 10.1021/jp810292n.

17. Tolbatov I, Marrone A. Computational Strategies to Model the Interaction and the Reactivity of Biologically-Relevant Transition Metal Complexes. *Inorg. Chim. Acta* (2022) 530:120686. doi: https://doi.org/10.1016/j.ica.2021.120686.

18. Bryantsev VS, Diallo MS, Goddard Iii WA. Calculation of Solvation Free Energies of Charged Solutes Using Mixed Cluster/Continuum Models. *J. Phys. Chem. B* (2008) 112(32):9709-19. doi: 10.1021/jp802665d.

19. Coutinho A, Prieto M. Ribonuclease T1 and Alcohol Dehydrogenase Fluorescence Quenching by Acrylamide: A Laboratory Experiment for Undergraduate Students. *J. Chem. Edu.* (1993) 70(5):425-8. doi: 10.1021/ed070p425.

20. Marques JT, de Almeida RFM. Application of Ratiometric Measurements and Microplate Fluorimetry to Protein Denaturation: An Experiment for Analytical and Biochemistry Students. *J. Chem. Edu.* (2013) 90(11):1522-7. doi: 10.1021/ed300599d.

21. Sergi B, Bulut I, Xia Y, Waller ZAE, Yildizhan Y, Acilan C, et al. Understanding the Potential in Vitro Modes of Action of Bis(Β-Diketonato) Oxovanadium(Iv) Complexes. *ChemMedChem* (2021) 16(15):2402-10. doi: https://doi.org/10.1002/cmdc.202100152.

22. Pessoa JC, Correia I. Misinterpretations in Evaluating Interactions of Vanadium Complexes with Proteins and Other Biological Targets. *Inorganics* (2021) 9(2). doi: 10.3390/inorganics9020017.

23. Levina A, Crans DC, Lay PA. Speciation of Metal Drugs, Supplements and Toxins in Media and Bodily Fluids Controls in Vitro Activities. *Coord. Chem. Rev.s* (2017) 352:473-98. doi: 10.1016/j.ccr.2017.01.002.

24. Nunes P, Correia I, Marques F, Matos AP, dos Santos MMC, Azevedo CG, et al. Copper Complexes with 1,10-Phenanthroline Derivatives: Underlying Factors Affecting Their Cytotoxicity. *Inorg. Chem.* (2020) 59(13):9116-34. doi: 10.1021/acs.inorgchem.0c00925.

25. Carter DC, Ho JX. Structure of Serum Albumin. *Adv. Protein Chem.* (1994) 45:153-203. Epub 1994/01/01. doi: 10.1016/s0065-3233(08)60640-3.

26. Ribeiro N, Galvão AM, Gomes CSB, Ramos H, Pinheiro R, Saraiva L, et al. Naphthoylhydrazones: Coordination to Metal Ions and Biological Screening. *New J. Chem.* (2019) 43(45):17801-18. doi: 10.1039/C9NJ01816F.

27. Lakowicz WR. *Principles of Fluorescence Spectroscopy*. 3rd ed: Springer (2006).

28. Gans P, Sabatini A, Vacca A. Investigation of Equilibria in Solution. Determination of Equilibrium Constants with the Hyperquad Suite of Programs. *Talanta* (1996) 43(10):1739-53. Epub 1996/10/01. doi: 10.1016/0039-9140(96)01958-3.

29. Mergny JL, Lacroix L. Analysis of Thermal Melting Curves. *Oligonucleotides* (2003) 13(6):515-37. Epub 2004/03/18. doi: 10.1089/154545703322860825.

30. LePecq JB, Paoletti C. A Fluorescent Complex between Ethidium Bromide and Nucleic Acids. Physical-Chemical Characterization. *J. Mol. Biol.* (1967) 27(1):87-106. Epub 1967/07/14. doi: 10.1016/0022-2836(67)90353-1.
